# A systematic review of economic analyses of psychological interventions and therapies in health-related settings

**DOI:** 10.1186/s12913-022-08158-0

**Published:** 2022-09-07

**Authors:** Leeanne Nicklas, Mairi Albiston, Martin Dunbar, Alan Gillies, Jennifer Hislop, Helen Moffat, Judy Thomson

**Affiliations:** 1 NHS Education for Scotland, 2 Central Quay, Glasgow, Scotland, UK; 2 Stobhill Hospital, NHS Greater Glasgow and Clyde, Glasgow, Scotland, UK; 3 Healthcare Improvement Scotland, Glasgow, Scotland, UK; 4 NHS Grampian, Aberdeen Royal Infirmary, Aberdeen, Scotland, UK

**Keywords:** Cost-effectiveness, Psychological interventions, Cognitive behavioural therapy, Long term conditions, Chronic pain, Cancer, Diabetes, Weight management

## Abstract

**Background:**

This review aims to synthesise evidence on the economic impact of psychological interventions and therapies when applied to a broad range of physical health conditions.

**Methods:**

The following bibliographic databases were searched for relevant articles: MEDLINE (Ovid), EMBASE (Ovid) and PsycINFO (Ebsco). As this review was intended to update an earlier review, the date range for the search was restricted to between January 2012 and September 2018. Reference lists from the review articles were also searched for relevant articles. Study quality was evaluated using the Scottish Intercollegiate Network Guidelines (SIGN) appraisal checklists for both economic studies and Randomised Controlled Trials (RCTs). When the economic analyses did not provide sufficient detail for quality evaluation, the original RCT papers were sought and these were also evaluated. Half of the papers were quality rated by a second author. Initial agreement was high and all disagreements were resolved by discussion.

**Results:**

This yielded 1408 unique articles, reduced to 134 following screening of the title and abstract. The full texts of the remaining articles were reviewed by at least one team member and all exclusions were discussed and agreed by the team. This left 46 original research articles, alongside five systematic reviews. Fifty-seven per cent of the articles were deemed to be of high quality, with the remainder of acceptable quality. Fifteen different medical conditions were covered, with chronic pain (10 articles) and cancer (9 articles) being the two most investigated health conditions. Three quarters of the papers reviewed showed evidence for the cost-effectiveness of psychological interventions in physical health, with the clearest evidence being in the field of chronic pain and cancer.

**Conclusions:**

This paper provides a comprehensive integration of the research on the cost-effectiveness of psychological therapies in physical health. Whilst the evidence for cost-effectiveness in chronic pain and cancer is encouraging, some health conditions require further study. Clearly, as the primary research is international, and was therefore conducted across varying health care systems, caution must be exercised when applying the results to counties outside of those covered. Despite this, the results are of potential relevance to service providers and funders.

**Supplementary Information:**

The online version contains supplementary material available at 10.1186/s12913-022-08158-0.

## Background

### Long term conditions

As people are living longer, many develop one or more long-term physical health problems as they age. These health problems include conditions such as diabetes, cardiovascular disease, asthma, blood borne viruses, neurological conditions, musculoskeletal conditions, as well as certain cancers. These conditions are often not able to be cured and must instead be managed. Prevalence is high, with the 2017 Scottish Health Survey [1] finding that 45% of adults reported at least one long term condition (LTC). It is estimated, therefore, that around 2 million people in Scotland are living with at least one long-term condition [2]. It is long established that long term conditions account for a significant portion of health care costs. For example, people with LTCs account for 50% of all GP appointments, 64% of all outpatient appointments, and 70% of all inpatient bed days [3].

Each specific LTC will have their own challenges and the extent to which people are able to live long and fulfilling lives varies enormously. One of the well-known difficulties is that having a LTC increases the chance of also having a co-morbid mental health problem [4]. These co-morbid mental health problems can add significantly to the burden of living with LTCs, as well as to the cost to health care systems.

### The burden of long-term conditions and co-morbid mental ill-health

Although the rates of co-morbid mental health problems are, to some degree, dependent on the specific health condition involved, these problems are very much higher across all long-term health conditions. A report for the Kings Fund [4] cites evidence showing that people with LTCs, such as cardiovascular disease, diabetes, chronic obstructive pulmonary disease and chronic musculoskeletal disorders, are two to three times more likely to experience mental health problems than are the general public without a LTC. An epidemiological study of 1.75 million primary care patients in Scotland published around the same time has provided precise statistics on the likelihood of having a co-morbid mental health problem depending on the number of co-morbid physical health problems that a person has [5]. This suggests that for those people with five or more physical morbidities, the chances that they will also have a co-morbid mental health problem are over six and half times more likely than for those people without any physical health problems. Even those with a single physical health condition have roughly double the risk of also having a mental health condition.

### Why physical and mental ill health co-vary

The reasons why physical and mental ill health show this pattern of covariance are complicated and it is likely that the mechanisms involved are multi-factorial, involving a combination of biological, psychological, environmental and behavioural factors [6]. Prince and colleagues [6] give a number of examples of how poor physical health increases the risk of developing poor mental health as well as a number of examples of poor mental health increasing the risk of the development of physical health problems. In addition to this is the growing recognition of the links between adverse childhood experiences and ill health [7]. Regardless of the mechanisms behind the co-variance of these conditions, it is widely accepted that when physical and mental ill health are both present they can interact to exacerbate each other. One example is given by a meta-analysis that found that, following a myocardial infarction, patients who also had co-morbid depression had an increased risk of further adverse cardiac events of up to 2.71 times greater than in those without depression [8]. Another example, from a study looking at illness more broadly, showed that primary care patients with any one of the following conditions; diabetes, ischemic heart disease, COPD and asthma, were more likely to be admitted to hospital as an emergency if they were also rated as being depressed [9]. Another example is a recent study that showed that the treatment response to expensive biological agents were significantly worse in a large sample of British patients with rheumatoid arthritis who also reported depressive symptoms or a history of depression [10].

The three studies reported here are just examples from an enormous literature which consistently finds that mental ill health impacts on the course and treatment response of physical ill-health. Given that mental health often makes physical health worse, it is therefore not difficult to see how it will therefore increase the costs of the treatment and care of people with long-term physical health problems. Naylor and colleagues [4] estimate that the effects of co-morbid mental health problems raise total health care costs by at least 45% for each person with a long-term physical health condition. In financial terms, Naylor and colleagues [4] suggested that between 12% and 18% of all NHS expenditure on long-term conditions can be accounted for by them being frequently accompanied by poor mental health and wellbeing.

### The effects of emotional distress on long-term conditions

Evidence has been accumulating for some time to suggest that stress, which may or may not result in a mental health problem, can affect a number of biological systems, particularly the cardiovascular, neurological and immunological systems, such that it increases the likelihood of illness developing [11]. Through these mechanisms, as well as through the effects on illness self-management behaviours, emotional distress can affect the risk and outcomes of physical disease, such as stroke and myocardial infarction [12], or selected cancers [13], in a dose dependent way. Whilst notions of distress and mental health conditions, such as anxiety and depression, clearly overlap, some researchers have examined the extent to which their effects on outcomes can be distinguished, including developing measures to capture the specific challenges of living with certain conditions. “Diabetes-specific emotional distress” is one such measure and it has been described as a wider affective experience, to do with “living with a progressive and chronic condition” [14]. It has been shown to have a deleterious effect on self-management and a more consistent negative effect on biomedical outcomes, such as HbA1c, than has been demonstrated for depression [15]. Consequently, some psychological interventions have been aimed, not just at those with a clearly defined mental health problem, but at whole populations of people with LTCs who are experiencing greater stress as a consequence of their condition.

### The role of other psychological factors, aside from mental health and stress, in physical ill health

There are a host of other psycho-social variables that have been investigated as influencing the risk factors for illness, thereby potentially contributing causally to physical illness, exacerbating the symptoms of illness or interfering with the outcome of treatment. Many of these variables are not directly related to mental health or psychological distress. They range from patient attributions as to the cause of their illness, consistent, unhelpful, behavioural responses to symptoms, and include processes thought to buffer the effects of stress, such as perceptions of social support available in the patient’s social network. There are so many of these variables that is impossible to list them here. Their importance is reflected in the fact that modification of some of these variables is incorporated into a variety of psychological treatment packages. These packages may, or may not, include interventions designed to modify stress or emotional difficulties. One example is the cognitive behavioural packages aimed at treating primary insomnia, which focus on modifying patterns of behaviour and beliefs about sleep and insomnia. This package was developed in the 1990’s and, although modified slightly over the years, is now widely regarded as the first line of treatment for primary insomnia [16]. Other long-term health conditions have also been treated by packages of psychological treatment where the focus is more on cognitions and patterns of unhelpful behaviour, rather than necessarily focusing on resolving psychopathology (see, Hedman-Lagerlöf et al., [17*], for example).

### Applied psychology in the management of LTCs

Psychologists can be found working in physical health settings in increasing numbers. Figures provided by the Information Services Division of NHS Scotland [18] show a 45% growth in the numbers of clinical staff working in psychological services in physical healthcare settings between March 2011 and June 2019. As well as the growth in the number of psychologists, there has also been a growth in the number of others who are delivering psychological therapies [18], either as formal psychological therapists, such as cognitive behavioural therapists, psychotherapists or counsellors or, but less clearly documented, as members of other professions who have been trained to deliver psychological interventions, under the supervision of psychologists.

More broadly, psychologists have been involved in the design and delivery of significant training and coaching in order that the care delivered across the health and social care workforce is psychologically informed. This has involved ensuring that the healthcare workforce is skilled in recognising (as well as eliciting) the psychological needs of the patient/client group, as well as understanding how to access, or signpost, to resources or support services. Further training has been conducted so that many of the workforce are equipped to deliver skilled psychological care. This increased knowledge and additional competencies are aimed at improving relationship and communication skills, as well as enabling the delivery of psycho-educational approaches, alongside training in the use of specific psychological techniques in order to address specific difficulties. Additionally, some members of the workforce have been equipped with skills in enhanced psychological practice so that they can deliver psychological interventions, which are often guided by protocol. These psychological interventions can be targeted at all patients as part of a care pathway, or only offered to those who meet a certain criteria, such as in a stepped care treatment model (see, for example, Chambers et al., [19]). The psychological workforce, within physical healthcare settings, are trained to post-graduate level in order to deliver psychological therapies, either to treat the co-morbid mental health problems described earlier or to deliver specific types of therapy aimed at improving the broader problems of adjustment, disability and quality of life.

### Evidence for clinical effectiveness, but not cost-effectiveness

The effectiveness of psychological therapies and interventions in physical healthcare have been investigated extensively by numerous randomised controlled trials and, subsequently, summarised in systematic reviews and meta-analyses. This is an extremely large literature, but helpfully both NHS Scotland and NHS England have developed programmes to summarise these literatures and to make recommendations. Obviously, this information is of huge importance to those who commission and deliver such services. The Scottish Intercollegiate Guidelines Network and NHS Education for Scotland (NES) has led in this regard in Scotland. NES has compiled and published tables of evidence for psychosocial interventions for people with persistent physical symptoms (The Matrix) [20]. This details the evidence that supports the use of a variety of psychosocial treatments in a range of physical illnesses, including, asthma, cancer, cardiovascular disease, chronic fatigue syndrome, chronic kidney disease, chronic obstructive pulmonary disease, chronic pain, diabetes, irritable bowel syndrome, multiple sclerosis, osteoarthritis, rheumatoid arthritis, and obesity. In NHS England, the National Collaborating Centre for Mental Health has summarised the evidence provided by the National Institute for Health and Care Excellence (NICE) and has produced a specific pathway on evidence-based psychological therapies that are recommended for people with long term conditions and medically unexplained syndromes [21]. These documents make it clear that there is substantial evidence that psychological therapies in long term conditions are clinically useful.

Economic evaluations are typically conducted alongside clinical trials, using either the primary trial outcome or a secondary outcome (particularly if health state utilities are being measured as neither this outcome measure, nor the quality-adjusted life-years that can be derived from it are typically used to power clinical trials). Alternatively, data from a range of different published trials and/or other study designs can be used to populate an economic evaluation of the cost-effectiveness of interventions. The feasibility of this is dependent on the number of published studies for a particular intervention, and can be difficult if an intervention is new, which is often the case for national funding decisions about treatments. However, a literature-based evaluation can be warranted if the number of available studies is expected to be considerable, for example where a systematic review or wider evidence synthesis of the existing clinical literature is already being undertaken. Cost utility analysis (CUA) describes that the health benefit measure used to value the cost of interventions in an economic evaluation is quantified in terms of quality-adjusted life-year (QALY) gains (i.e. cost per QALY gained). This type of analysis is preferred by organisations making national NHS funding decisions about the value of interventions. Cost-effectiveness analysis is also commonly used, whereby health benefits have been quantified in their natural units e.g. per point increase on a condition-specific measurement scale. A glossary for these terms is included at the end of the paper.

### The development of digital interventions and therapies

The recent global coronavirus pandemic has led to a rapid and large shift in the ways in which psychological therapy is delivered. Due to the social distancing restrictions that the virus has imposed, many therapies are currently being delivered remotely, either via the telephone or over the internet. Studies that have evaluated the effectiveness of therapies delivered through these remote modalities have been accumulating gradually over the past twenty years [22], but the coronavirus pandemic has led to a swift acceleration in the efforts to synthesise this emerging literature (see, for example, Eccleston, et al., [23]). Any report on the cost-effectiveness of psychological interventions and therapies must recognise the potential that remote delivery has to reduce costs by, for example, eliminating or reducing the need for expensive clinical environments. These cost savings, however, might be counterbalanced by the need to provide specific software and IT infrastructure.

### Evidence thus far for the cost-effectiveness of psychological therapies in physical health

The evidence for the cost effectiveness of psychological therapies in physical health is dispersed across the different bodies of literature dealing with the specific conditions, and the different therapeutic approaches, that have been investigated thus far. To date, there have been very few attempts to take a broad view across all these different literatures. One paper that did attempt this was the narrative review previously published by the Psychology and Physical Health team at NHS Education Scotland [24]. Instead of taking a condition specific approach, as we have done on this occasion, different approaches to providing psychological care were examined, including integrating psychology into programmes that seek to aid in the management of chronic disease. This document reported studies suggesting that such approaches saved money when treating co-morbid depression in diabetes, reduced hospital admissions in angina patients, and that CBT when given to patients with somatoform condition has the potential to substantially reduce sickness absence and its associated costs.

Helpfully, there have also been some systematic reviews, although these are few in number. Examples include, systematic reviews of studies looking at the cost-effectiveness of psychological treatments in cancer [25] which concluded that, whilst the field was still young, there was emerging evidence of cost-effectiveness. Another example is provided by Jeeva and colleagues [26] who examined the cost effectiveness of psychological interventions in diabetes care. Interestingly, a number of these interventions were not specially about a single form of therapy, but rather looked at psychological approaches being integrated into programmes of collaborative care (a system of multidisciplinary team-based care which involves a care manager and a patient management plan). They concluded that such approaches were cost-effective when compared to usual care. Another example is the review of the cost-effectiveness of interventions in insomnia that was conducted by Wickwire and colleagues [27]. This examined three studies with findings suggesting a strong probability that psychological interventions were cost-effective. However, these reviews are unlikely to cover all of the areas where the cost-effectiveness of psychological approaches have been conducted. The aim of this review is to systematically survey and summarise this literature.

## Methods

### Protocol and registration

The protocol for this review was registered with the PROSPERO prospective register of systematic reviews (CRD42019136922).

### Eligibility criteria

Studies that met the following criteria were eligible for inclusion:

- Interventions involving a psychological therapy or approach (i.e. informed by psychological theory) and involving a staff member (including in conjunction with other interventions such as education/physical activity)
- Participants with a physical health condition (e.g. diabetes) or receiving services in a physical health care setting (e.g. primary care clinic)
- A developed country setting
- Based on a randomised trial
- Inclusion of data on health care utilisation and/or cost-effectiveness (could include utility values used to derive QALYs)
- Published in English

Studies were excluded if they related specifically to substance abuse services, mental health treatment for patients with either dementia or learning disabilities, education-only interventions or interventions that did not involve a health professional (for example those interventions that were solely digital), interventions involving a primarily paediatric population (i.e. if 80% or more participants were aged under 16) and studies that only looked at the cost of interventions (unless a cost-minimisation analysis had been performed and an assumption of equivalence/non-inferiority of the interventions had been stated). We also included systematic reviews and relied on these to summarise the literature prior to 2012 as well as using them for citation searching for eligible RCTs.

### Information sources and search strategy

The following bibliographic databases were searched: MEDLINE (Ovid), EMBASE (Ovid) and PsycINFO (Ebsco). All searches were completed in September 2018. Database search results were limited to publications in English from 2012 onwards.

The current review was prompted by a previous unpublished NHS Education for Scotland report, *Psychological interventions in physical health care: the need and the economic case* [24], which had drawn on cost-effectiveness evidence from a 2012 Kings Fund report [4].

Reference lists from relevant systematic reviews and literature reviews were also searched to identify studies that met the criteria for this review, and to provide background information on the state of the evidence base prior to 2012. Forward citation tracking was performed on relevant review papers to identify additional studies. The full search strategy for each database is available in an appendix.

### Study selection

All results were downloaded to a reference management software package (RefWorks) and duplicates were removed, leaving 1408 unique references.

Initial screening on title/abstract was conducted by one team member (AG) who proposed a list of exclusions. All initial screening decisions were double checked. The team reviewed the inclusions and two authors (LN and MA) reviewed the proposed exclusions. Any disagreement over an exclusion was discussed by the team as a whole. During this stage 1274 references which did not meet the inclusion criteria were excluded.

The remaining 134 articles were screened by the team as a whole. Individual team members examined the full texts of the articles in their topic areas and fed back their recommendations for inclusion or exclusion to the team. Any disagreements were discussed and decided by the whole team. The main reasons for exclusion at this stage were that studies: had a non-adult sample, were non-randomised, did not involve a psychological intervention, were reported in a conference abstract with no further information available or provided insufficient cost data.

In addition, a decision was taken to exclude all studies focused on Chronic Fatigue Syndrome/Myalgic Encephalomyelitis. This decision was made in light of the current review (NICE 2020) of the relevant NICE guideline and because of concerns that have been expressed over one of the major studies in this area [28].

**Fig. 1:**
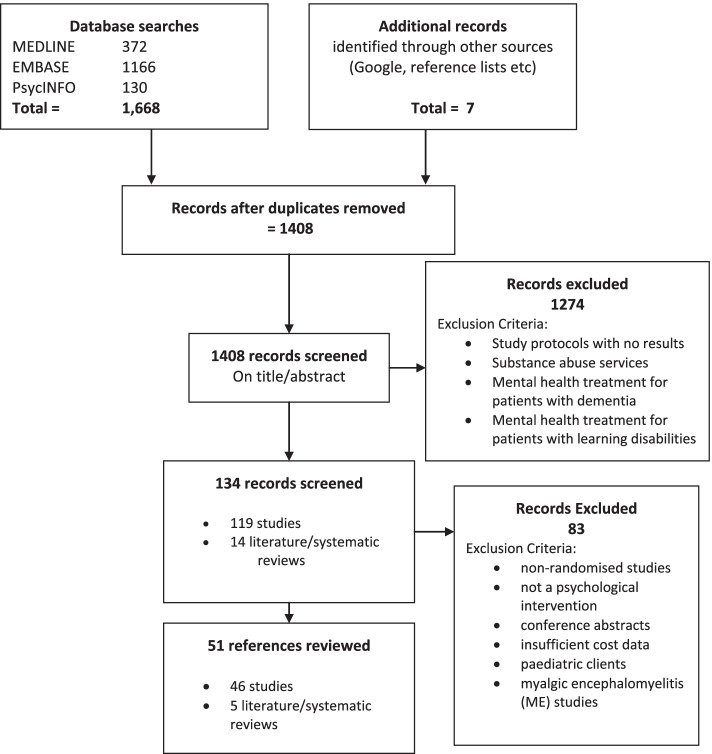
PRISMA flow diagram

This resulted in the exclusion of a further 82 papers. Forty six studies and 5 review articles were included in the final synthesis. This is summarised in a PRISMA diagram (Fig. 1).

### Data collection and measures used

The task of data extraction was split between all members of the team using a pro forma developed for the review. Data extraction was also double checked by a second author (JH).

The following data were extracted: study country and setting; the health condition(s) of interest; the intervention and control treatments; frequency of follow up time points; the number of study participants and their characteristics; the perspective taken for any cost modelling; the time horizon and discount rate(s) used for costs and benefits; the resources (itemised where possible) used; currency, price year and unit costs applied.

Outcome data were extracted on incremental cost-effectiveness ratios (ICERs), where these had been reported, and health related benefits seen (e.g. QALYs and/or the utility scores used to derive them).

If a study has provided multiple ICERs because it explored different average costs and/or outcomes separately for the ITT analysis, per protocol analysis and/or completers analysis, the ITT analysis ICERs were chosen where possible.

If a study provided cost-utility ICERs and cost-effectiveness ICERs, then we primarily used the cost-utility analysis ICERs because it allowed us to explore comparisons across studies. Where no cost-utility analysis was available but the study provided multiple ICERs for cost-effectiveness analyses, then we used the ICER relating to the primary outcome where possible.

### Quality appraisal/risk of bias

The quality of each study was assessed using the Scottish Intercollegiate Guidelines Network methodology [29], which rates controlled trials as High, Acceptable or Low quality and economic studies as High, Acceptable or Unacceptable. For each of our included studies, both the SIGN Economic Evaluations checklist and the SIGN Controlled Trials checklist were used, giving a quality rating for the economic evaluation and a quality rating for the randomised controlled trial on which it was based. Where data were taken from an economic evaluation that had used a separately published RCT, the main RCT paper was also sought for additional details to help with the quality assessment.

As double-blinding of participants to treatment allocation is usually regarded as impractical in RCTs of psychological interventions [30], only the blinding of the outcome assessors was considered when using the SIGN RCT checklist to rate the quality of the papers.

Quality ratings were conducted by clinicians with expertise in the field, of which half were double rated and any differences were discussed to reach agreement. All of the economic evaluation ratings were checked by another member of the team (JH). For the Economic Evaluations checklist, there was initial agreement on 20 of the 24 papers double-rated (83%) and for the RCT checklist there was initial agreement on 14 of the 24 papers (58%). Agreement was reached after discussion by the two raters on all papers except one, which was discussed and agreed by the team as a whole.

### Synthesis of results

No meta-analysis was possible due to the heterogeneity of interventions and study outcomes. A narrative synthesis of the available research was therefore performed.

Across the previous systematic reviews there were 73 included studies. Of these, four were also captured as primary studies in this review. A summary of these reviews is presented in Table 1. The proportion of included studies in each review that were also identified as primary studies for this review ranged from 0% [26, 31] to 20% [27]. The low proportion of overlap is likely due to the date cut off in our inclusion criteria compared with the search strategy dates for these reviews. The conclusions of the reviews were generally positive, and given the low proportion of overlap, likely adds weight to the evidence base identified from the primary studies included in this review for each condition, although in some cases the interventions included in these reviews may not meet the inclusion criteria for this review (for example, the study by Wickwire et al., 2016 [27] also includes pharmacological therapies.

**Table 1:** Summary of systematic reviews

Study	Area (focus)	Title/Purpose of Review	Databases Searched (Review Search Dates)	Number of included studies	Review Conclusions	Quality Rating (SIGN sys. Rev. checklist)
Wickwire et al., 2016 [27]	Insomnia	Reviews the economic consequences of insomnia and the cost effectiveness of insomnia treatment	Not stated	Ten published studies, of which 2 (Watanabe 2014 and Bonin 2014) are included as primary studies in our review.	Both pharmacologic and behavioural treatments yield substantial savings in terms of reduced health care utilisation costs and improve health-related quality of life within accepted ranges of cost-effectiveness (even when excluding reductions in indirect costs). Costs were typically recouped within six and 12-months. Study periods.	Low quality
Dieng et al., 2016 [25]	Cancer	Assess the cost-effectiveness of psychosocial interventions for improving psychological adjustment among people with cancer	Medline, Medline In-Process, Embase, PsycINFO, Cumulative Index to Nursing and Allied Health Literature, Econlit, Cost-Effectiveness Analysis Registry (CEA Tufts) and the National Health Service Economic Evaluation Database (1980 to May 2015)	Eight studies, of which one (Arving 2014) [32] is included as a primary study in our review.	Several psychosocial interventions, particularly those based on cognitive-behavioural therapy, have been demonstrated to represent good value for money in cancer care.	High Quality
Jeeva et al., 2013 [26]	Diabetes	Identifies current economic evidence of psychological treatments for depression among people with diabetes	Medline, Embase, PsycINFO, CINAHL, and NHS Economic Evaluation Database (NHS EED) databases (January 2000 to May 2012)	Four economic evaluations were identified. There is no overlap with primary studies in our review.	Studies indicated the potential of interventions to be cost-effective compared with usual care. Two studies reported costs per QALY gained of USD 267 to USD 4317, whilst two studies reported the intervention dominated usual care.	Acceptable
McCombie et al., 2013 [31]	IBD	Systematically reviewed all randomized controlled trials that have been performed in psychotherapy for inflammatory bowel disease patients (cost effectiveness section)^a^	PsycINFO, MEDLINE, EMBASE Cochrane Library. Searches were performed on the databases on 1 and 8 March, 2012, with limits to the years of 2010–2012.	In total, eighteen studies (nineteen papers) were included in this review. There is no overlap with primary studies in our review.	Psychotherapy for IBD has minimal effect on measures of anxiety, depression, QOL and disease progression. It shows promise in reducing pain, reducing fatigue, reducing relapse and hospitalisation, improving medication adherence and may be cost-effective. We also recommend that computerised CBT is evaluated given its high acceptability and low cost.	Acceptable
Andronis et al., 2017 [33]	Pain	Systematic review of the cost-effectiveness of non-invasive and non-pharmacological treatment options for lower back pain (LBP).	EMBASE, MEDLINE, PsycINFO, Cochrane Library, CINAHL and NHS Economic Evaluation Database (January 2000 to July 2015)	Thirty-three studies are included, of which one (Norton 2015) is included as a primary study in our review.	Combined physical and psychological treatments, medical yoga, information and education programmes, spinal manipulation and acupuncture are likely to be cost-effective	Acceptable

^a^One of the studies referenced in the cost-effectiveness section is not listed as an included study

## Results

**Fig. 2:**
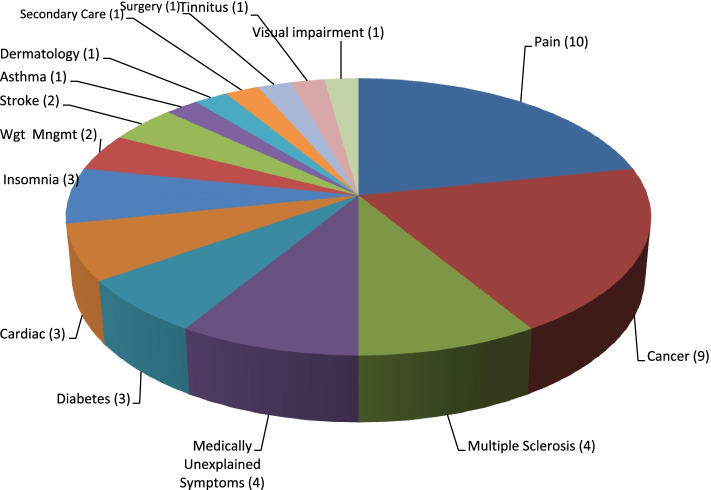
Number of studies by medical condition

Forty six studies were identified which met the inclusion criteria for the review. All were reviewed using SIGN checklists in relation to the original RCT and the cost effectiveness study and were deemed by the review team to be of acceptable or high standard (see Table 2). Studies were grouped according to health condition of the participants, with the largest number of studies being in the area of chronic pain and cancer (Fig. 2). The studies are detailed in the tables and narrative descriptions below.

**Table 2:** SIGN quality ratings of included studies

Economic study (RCT paper if separate)	SIGN RCT Checklist	SIGN Economic Checklist	Notes/issues
Arving et al., 2014 [32*] (RCT – Arving et al., 2007) [34]			
Bennell et al., 2016 [35*]			
Bogosian et al., 2015 [36*]			Small sample size of 40 may limit confidence.
Bonin et al., 2014 [37*] (RCT – Swift et al., 2012) [38]			
Camacho et al., 2016 [39*] (RCT - Coventry et al., 2015 [40])			
Chatterton et al., 2016 [41*] (RCT – Chambers et al., 2014) [42]			Comparison of 2 interventions, no control/ TAU
Chernyak et al., 2014 [43*] (RCT – Sattel et al., 2012) [44]			
De Boer et al., 2014 [45*]			Small sample size with signficant drop out rates, therefore underpowered to detect no difference, which was the study’s hypothesis
Goossens et al., 2015 [46] (RCT – Leeuw et al., 2008) [47]			Small sample size and therefore likely underpowered to detect differences between two active treatments
Hedman-Lagerof et al., 2019 [17*] (RCT - Hedman-Lagerlof et al., 2018) [48]			
Herman et al., 2017 [49] (RCT – Cherkin et al., 2016) [50]			Note that MBSR experimental group received an additional six hours of treatment (one day retreat) compared to the active control
Hersey et al., 2012 [51*]			
Humphreys et al., 2013 [52*] (RCT - Lincoln et al., 2011) [53]			
Humphreys et al., 2015 [54*] (RCT – Thomas et al., 2013 [55*, 56])			
Ismail et al., 2018 [57*]			Training did not change nurses skills beyond the proficiency of those offering standard care on competency measures so limited differences between control and intervention.
Jansen et al., 2017 [58*] (RCT – Krebber et al., 2016) [59]			
Johanssen et al., 2017 [60*] (RCT- Johanssen et al., 2016) [61]			Economic analysis based on assumption of 5-20 year survival after treatment
Kemani et al., 2015 [62*]			Small sample size may limit confidence
Ladapo et al., 2012 [63*] (RCT - Davidson et al., 2010 [64])			
Larsen et al., 2016 [65*] (RCT – Larsen et al., 2014) [66]			
Lengacher et al., 2015 [67*] (RCT – Lengacher et al., 2009) [68]			
Luciano et al., 2013 [69*] (RCT – Luciano et al., 2011) [70]			
Luciano et al., 2014 [71*]			
Luciano et al., 2017 [72*]			
Maes et al., 2014 [73*] (RCT – Cima et al., 2012) [74]			
Mejia et al., 2014 [75*] (RCT - Cockayne et al., 2014) [76]			
Mewes et al., 2015 [77*] (RCT – Duijts et al., 2012) [78]			
Mosweu et al., 2017 [79*] (RCT - Moss-Morris et al., 2012) [80]			Small sample size may limit confidence.
Nobis et al., 2018 [81*] (RCT – Nobis et al., 2015) [82]			
Norton et al., 2015 [83*] (RCT – Lamb et al., 2010 [84])			
Parry et al., 2012 [85*]			
Perri et al., 2014 [86*]			High quality study. Only rural population analysed.
Prioli et al., 2017 [87*] (RCT – Monti et al., 2013) [88]			
Rolving et al., 2016 [89*] (RCT – Rolving et al., 2015) [90]			
Schroder et al., 2017 [91*] (RCT - Schroder et al., 2012) [92]			
Thiart et al., 2016 [93*]			
Thomas et al., 2013 [55*, 56]			
Tyrer et al., 2014 [94*]			
Tyrer et al., 2017 [95*]			Small N of 34 in each arm. Type of training, supervision, and protocol adherence monitoring not outlined in the paper.
Van der Aa et al., 2017 [96*]			
Van der Spek et al., 2018 [97*] (RCT - van der Spek et al., 2017) [98]			
Van Eeden et al., 2015 [99*] (RCT protocol – Kootker et al., 2012) [100]			
Van Ravesteijn et al., 2013a [101*] (RCT - Van Ravesteijn et al., 2013b) [102]			
Visser et al., 2015 [103*] (RCT – Zonneveld et al., 2012) [104]			
Watanabe et al., 2015 [15] (RCT – Watanabe et al., 2011) [105]			
Zhang and Fu et al., 2016 [106*]			As well as 3 randomised groups, study included eligible patients declined intervention but agreed to give feedback

Twelve of the 46 studies which met the criteria for this review were undertaken in the UK, with the twenty-one from other European countries, seven from the US, one from Japan and two from Australia. In terms of the therapeutic modality of the psychological interventions, 30 out of 46 were cognitive behavioural therapy based interventions, with the others comprising a range of approaches including mindfulness, behavioural therapy, motivational interviewing and psychodynamic therapy. Twenty one out of 46 were delivered in a group format. In 9 studies an existing member of the multi-disciplinary team (a nurse or in 2 cases a physiotherapist) was trained to deliver the psychological intervention whereas 22 of the 46 studies, the intervention was delivered by a psychologist or psychotherapist. In the remainder of the studies, authors do not always explicitly state who delivered the intervention, but this was often an individual trained for the purpose of the research study. Four studies applied a stepped care model of care and two studies applied a collaborative model, whereas the majority offered a standard intervention to all participants who met the inclusion criteria.

Technology enabled delivery was a feature of 12 of the 46 studies cited in this review across a wide range of health conditions, including remote delivery via video or telephone, internet enabled packages which were either guided clinicians, interactive or supported by email feedback.

Amongst the traditional interventions delivered by video, Bogosian et al. [36*] report that mindfulness-based CBT delivered via Skype, was clinically effective in reducing distress in patients with MS and cost effective, although the number of participants was small. Within the area of chronic pain, Hedman-Lagerof et al. [17*] report that internet delivered exposure therapy for participants with fibromyalgia was highly effective and associated with significant cost savings. The results of de Boer et al. [45*] comparison of internet-based CBT with face to face CBT for nonspecific chronic pain, were less clear in terms of cost effectiveness.

Five studies included telephone delivered psychological therapy. Chatterton et al. [41*] compared 5 sessions of telephone-based CBT with a single telephone support session and found the CBT intervention to be cost effective for highly distressed cancer patients and carers. A telephone based motivational interview intervention for patients with psoriasis was found to be more cost effective than a physical therapy [65*]. In two other studies interventions deemed to be cost-effective were delivered to some participants by telephone, while other participants in the same condition were offered the same intervention face to face [32, 106]. Mosweu et al’s [79*] study of nurse- led CBT for distressed patients with MS reports a mixture of telephone and face to face delivery. The authors do not indicate if these different modes of delivery have a differential impact on cost effectiveness.

A web based treatment package for depression guided by coaches for patients with diabetes, was a found to cost effective compared to web based psycho-education, in a study by Nobis et al. [81*]. A web based self-management programme with telephone support for MS was also likely to be cost-effectiveness but the sample size was small [80]. In another web based intervention for weight management [51*], retention rates were also cited as a concern. In this study the most clinically effective change was reported in the group in which the interactive web-based programme was supplemented with phone or email support, however, all conditions were found to be cost effective when compared with projected medical costs. Thiart et al. [93*] also combined an internet-based programme, in this case CBT for insomnia, with email feedback, which was again found to be a cost-effective intervention.

Of the included studies 21 had used the EQ-5D and 13 had used either the SF-6D, SF-12 or SF-36, including one study which had used both [55*]. One study [62*] had collected SF-36 data but not used this to derive QALYs or conduct a cost-utility analysis. Another study provided EQ-5D data from a separate paper [84] to that included in the review [83*]. Seven studies had used other measures to estimate health state utilities, including two using AQol [35, 41], one using the HUI Mark III [73*], another using the 15D instrument [65*]. Three studies had used mapping from condition-specific instruments or literature derived utility weights [32, 51, 107].

A wide range of condition-specific outcomes were used in the studies that had evaluated cost-effectiveness without using utility scores. Common measures related to depression and/or anxiety (e.g. the Hospital Anxiety and Depression Score, the Beck Depression Inventory, the number of depression-free days), pain (e.g. the Pain intensity Scale or another pain visual analogue scale, the Pain Disability Index, CPAQ for pain acceptance), fatigue (e.g. the fatigue severity scale, fatigue assessment instrument or another fatigue VAS).

The setting for 21 studies was hospital-based, although in many cases participation will have been sought at outpatient clinics rather than wards. Seven studies recruited through primary care and a further 5 studies recruited from across the health and/or social care system.

The currency used reflected the setting of each study (i.e. it was the currency used in the country where the study took place). In six studies, results were provided in a currency different from that used in the study country, either in addition to the home currency or instead of it [17, 54, 62, 73, 83, 107]. The study price year was also linked to the year of the study publication, although on average it took 4 years from the price year to the year of publication.

Sample size ranged from 34 to 1755, with a median sample size of 157 (IQR: 104 to 260). Median time horizon was 52 weeks (IQR: 26 to 71.5 weeks), reflecting the durations of clinical trial follow up in most cases.

Perspectives are difficult to summarise due to reporting and the variety of different country settings in the review (whereby different methods of funding healthcare may exist). Healthcare (health service or health and social care) perspectives only occurred in 19 studies, and the thresholds used to define cost-effectiveness depended on currency but excluding a zero WTP, ranged from US $50,000 to US $100,000 (the same range as for Euros), GBP£15,000 to GBP £60,000 and AUS $50,000.

Many studies reported that they had used broader perspectives, usually in addition to a healthcare or health and social care perspective rather than alone. This was usually to account for wider costs and benefits associated with employment in terms of absenteeism or lost productivity, given the nature of the interventions. However, the situation is complicated in terms of summarising here as although many studies reported that they had taken a broader (e.g. societal) perspective, this was not always accompanied by reporting of separate ICERs or willingness to pay thresholds for the broader perspective. To some extent, this is easier to identify for studies that had used a cost-utility analysis where approximate threshold values for society’s willingness-to-pay for a QALY gain (itself a health benefit gain only), are widely known compared to other outcomes.

Most studies had undertaken some form of sensitivity analysis, and most commonly reported, in 32 studies, was bootstrapping (typically 1000 iterations). Where other sensitivity analyses had also been conducted these explored various scenarios (e.g. a longer time horizon) or specific values (e.g. intervention costs), changed the imputation method for missing data or included trial completers only or per protocol analysis participants.

## Chronic pain

### Previous systematic review of economic evaluations of psychological interventions in chronic pain

Only one previous systematic review was identified. This review was conducted by Andronis and colleagues [33] and was rated as being of acceptable quality. The review identified 12 studies and was restricted to studies where the participants had low back pain. However, the authors also included some studies where the participants had acute, rather than chronic, low back pain. It is therefore difficult to be confident that their conclusions apply directly to this review and caution should be used. They also considered other, non-psychological, interventions, such as acupuncture and physical therapy, although the results for the different therapies were reviewed separately. The authors concluded that combined physical and psychological treatments, medical yoga, information and education programmes, spinal manipulation and acupuncture are likely to be cost-effective options for LBP.

### Overview of chronic pain studies in current review

Nine studies were identified that had been published since 2012 and had examined the cost-effectiveness of psychological therapies in chronic pain (see Tables 3 and 4). Half of these studies employed samples of patients with Fibromyalgia, with the remaining four studies split equally between those that examined samples with chronic low back pain and those with samples who had undifferentiated, or non-specific, chronic pain. All of the studies were conducted in European countries (Spain, Sweden and the Netherlands) except for the study by Norton and colleagues who estimated costs in the United States using a Markov model, albeit with UK utility data.

**Table 3:** Description of studies: pain

Authors, Year	Condition	CEA	CUA	Setting	N (participants)	Baseline Characteristics	Intervention/ comparator(s)	Effectiveness measure(s); cost measures (price year)	Perspective	Time horizon
De Boer et al. 2014 [45*]	Pain	✓		Netherlands	N Participants:	72	Internet delivered, CBT-based, pain management course, with email feedback from psychologist (*N* = 22) Face-to-face CBT-based pain management course (*N* = 28) Both courses had 8 sessions (7 × 2 hr. sessions plus 1 × 2 hr. booster session 2mths later). Facilitated by ‘Trained Psychologist’	Pain Catastrophising Scale	Societal	15wks
				Pain Centre at Hospital, (Groningen)	Age in years (mean):	Internet: 50.6 (10.7); Face-to-face: 53.2 (11.7)		Euros (2013)		
					Sex (% female):	Internet: 68.2; Face-to-face: 60.7				
					Ethnicity (% white):	Not given				
Goossens et al. 2015 [46]	Pain	✓	✓	Netherlands	N Participants:	85	Graded Exposure (GE, *N* = 42) versus Graded Activity (GA, *N* = 43). GE involved developing a personalised hierarchy of feared movements and working through these (exposure) in a systematic fashion (16 × 1 hr. sessions). GA involved Education and treatment rationale plus 25x1hr sessions of gradually increasing activity. Both Interventions delivered by a team consisting of psychologist, physiotherapists and OT.	Quebec Back Pain disability Scale (used in CEA) QALYs (SF-36 used in CUA)	Health care, social and personal expenses, and lost productivity	12mths
				Outpatient rehabilitation centres	Age in years (mean, SD):	Graded Activity: 45.45 (8.42); Graded Exposure: 47.13 (9.58); Overall: 46.3 (8.98)		Euros (2014)		
					Sex (% female):	Graded Activity: 50; Graded Exposure: 50				
					Ethnicity (% white):	Not given				
Hedman-Lagerlof et al. 2019 [17*]	Fibromyalgia (FM)	✓	✓	Sweden (Stockholm); internet recruitment	N Participants:	140	i-EXP (internet delivered exposure therapy for pain; N = 70) versus WLC (waiting list control; *N* = 70). i-EXP group received 10 week programme of internet delivered education and exposure to Fibromyalgia and pain related stimuli. Psychological therapists qualified to at least Masters level.	Fibromyalgia Impact Questionnaire (FIQ used in CEA) QALYs (EQ-5D, used in CUA)	Costs include Direct medical costs and non medical costs as well as lost capacity	12mths
					Age in years (mean, SD):	i-EXP: 51.8 (10.7); WLC: 49.3 (10.0)		SKK (2016) converted to USD		
					Sex (% female):	i-EXP: 97; WLC: 99.				
					Ethnicity (% white):	Not given				
Kemani et al. 2015 [62*]	Pain	✓		Sweden (Stockholm); internet recruitment	N Participants:	60	ACT intervention (*N* = 30) delivered by Clinical Psychologists and an ACT trained physician. AR intervention (*N* = 30) delivered by Clinical Psychologists. ACT and AR both were 12 × 1.5 hr. weekly sessions	Pain Disability Index (PDI used in CEA)	Direct and indirect medical costs as well as some social costs	6mths
				Consecutive referrals from primary and secondary care	Age in years (mean, SD):	ACT = 38.7 (11.1); AR = 42.0 (11.6)		SKK converted to USD (2013)		
					Sex (% female):	ACT = 80; AR = 66.7				
					Ethnicity (% white):	Not given				
Luciano et al. 2014 [71*]	Fibromyalgia (FM)		✓	Spain (Zaragoza);	N Participants:	169	Three groups: A = CBT (*N* = 57) B = RPT (medication, *N* = 56) C = TAU (N = 56) CBT delivered by trained clinicians. The CBT intervention was delivered in groups over 9 sessions.	QALYs (EQ-5D-3L),	Direct and Indirect costs.	6mths
				Multicentre recruitment but not delivery; 41 general practices	Age in years (mean, SD):	CBT = 46.35 (6.71); RPT = 47.12 (6.25); TAU = 47.04 (6.53)		Euros (2011)		
					Sex (% female):	CBT = 94.7; RPT = 92.9; TAU = 96.4				
					Ethnicity (% white):	Not given				
Luciano et al. 2017 [72*]	Fibromyalgia (FM)		✓	Spain (Zaragoza);	N Participants:	156	Three groups: A = ACT (*N* = 51) B = RPT (Medication, *N* = 52) C = WLC *N* = 47). ACT group delivered by qualified, trained and experienced clinical psychologist. Participants received eight sessions of 2.5 hours group sessions (manualised and fidelity checked).	QALYs (EQ-5D-3L);	Both Direct and Indirect costs.	6 mths
				General practices (multicentre recruitment but not delivery)	Age in years (mean, SD):	ACT mean age = 48.88 (5.94), RPT = 47.77 (5.87), WL = 48.28 (5.71).		Euros (2014 price year)		
					Sex (% female):	ACT = 96.1 RPT = 98.1 WL = 94.3				
					Ethnicity (% white):	Not given				
Luciano et al. 2013 [69*]	Fibromyalgia (FM)		✓	Spain (Zaragoza);	N Participants:	216	Intervention group (*N* = 108) received 5 × 2 hr. group sessions of education and 4 × 2 hr. group sessions on autogenic training (relaxation). Staff mainly clinical psychologists plus one rheumatologist. Waiting list control (N = 108) consisted of medication as usual and also received counselling on importance of exercise.	QALYs (EQ-5D-3L);	Both Direct and Indirect costs.	12 mths
				General practices (multicentre recruitment but not delivery)	Age in years (mean, SD):	Intervention: 55.17 (8.58); Control: 55.42 (8.63)		Euros (2008 price year)		
					Sex (% female):	Intervention group: 97.2%; Control: 98.1%				
					Ethnicity (% white):	Not given				
Norton 2015 [83*]	Pain		✓	UK data applied to US database	N Participants:	701	All study participants received Active Management (15 min with nurse) and The Back Book. The control group (*N* = 233) received nothing further. The intervention group (*N* = 468) also received 6 CBT group sessions, 90 mins long, delivered over 6 weeks. Delivered by a mixture of professionals, including psychologists Intervention group	QALYs (EQ-5D-3L from Lamb 2010 data) [84];	Health care costs only	10 yrs. (modelled)
				(Lamb et al. 2010 [84] data) applied to US insurance claims data	Age in years (mean, SD):	54(15) [NB: From UK Lamb et al. 2010 [84] study which were applied to the US data]		GBP (2008 price year)/ USD (price year not reported)		
					Sex (% female):	60				
					Ethnicity (% white):	88				
Herman 2017 [49]	Pain		✓	USA (Washington State); recruitment from “integrative healthcare system”	N Participants:	342	MBSR (*N* = 116) vs CBT (*N* = 113) vs Usual Care (N = 113). CBT intervention delivered by psychologists over eight, weekly, 2 hr. sessions. MBSR intervention delivered by trained MBSR instructors over eight, weekly, 2 hr. sessions. MBSR group also received a 6 hr. retreat in addition to the group sessions.	QALYs (SF-12)	Payer and societal perspective	1 yr
					Age in years (mean, range):	49 (20-70)		USD (2013)		
					Sex (% female):	66				
					Ethnicity (% white):	82.50				
Bennell et al., 2016 [35*]	Pain (osteoarthritis)		✓	Australia; Community	N Participants:	222	Cognitive and behavioural pain coping skills training (PCST (*N* = 74), versus exercise (*N* = 75), versus PCST and exercise combined (*N* = 73); All had 10 individual sessions with a physical therapist over 12 weeks; Therapists had ‘extensive’ PCST training from psychologists	QALYs (AQOL-6D)	Societal	12mths
					Age in years (mean, SD):	Gp 1: 62.7 (7.9), Gp 2: 63.0 (7.9), Gp 3: 64.6 (8.3).		A$ (Australian) (2012)		
					Sex (% female):	Gp 1: 59, Gp 2: 61, Gp 3: 60.				
					Ethnicity (% white):	Not given

**Table 4:** Cost-effectiveness Outcomes: Pain

Authors, Year	Type of Analysis	Type of Costs	Costs	Type of effectiveness outcome	Effectiveness	ICER (definition);	ICER (results)	Sensitivity Analysis (definitions)	Sensitivity analysis (results)	Authors conclude (Reviewer comments where these differ from authors)
De Boer et al. 2014 [45*]	CEA/CUA (SF-36 - RAND)	Total costs	Intervention: €1745; Control: €1717; Difference: €28 (CI = − 1293 to 1338)	PCS at baseline, 7 weeks and 15 weeks	Intervention: 19.82 (13.88) at baseline, 12.55 (11.53) at 7 weeks and 11.00 (11.49) at 15 weeks; Control: 20.38 (11.38) at baseline, 17.13 (12.49) at 7 weeks and 16.10 (11.56) at 15 weeks; Difference of 5 points on the PCS were gained	ICER (per every additional PCS point improvement):	No ICER provided for seven week end of treatment period. PCS was five points lower (better) in internet group, giving an ICER based of 40 (CI = − 228 to 56) meaning that for every additional point improvement on the PCS, 40 Euros is saved. Internet treatment is dominant.	Bootstrapping (Y/N; iterations):	Bootstrapping (5000 replications)	Internet course was cost-effective compared to the group course. Conclusions: We conclude that the Internet-based cognitive-behavioural intervention was at least as effective as the face-to-face group intervention and, on some outcome measures appeared to be even more effective (unclear - poorly reported particularly sensitivity analysis details).
		Medication costs	Intervention: €175; Control: €208; Difference: €33. Not significant (95% CI:-185 to 114);	F test (Group by time interaction) at 7 weeks and 15 weeks	2.891 (*p* = 0.096) at 7 weeks, 5.546 (*p* = 0.023) at 15 weeks					
		HCP contacts/Admissions	Intervention: €649; Control: €707; Difference: €58. Not significant (95%CI: − 600 to 386)							
		Productivity losses	Intervention: €922; Control: €802; Difference: €120. Not significant (95%CI: − 1065 to 1324)							
Goossens et al. 2015 [46]	CUA	Total costs (SD), included number of sessions multiplied by the costs of the treatment team, plus expenses.	Intervention (EXP): €10,843.50 (1747.89); Intervention 2 (GA): €13,477.71 (2450.28); Difference: GA was €2643 (CI = − 8535 to =3058) more expensive due to greater number of sessions including a psychologist	Mean (SD) utility from SF-36	Intervention: 0.66 (0.14); Control: 0.68 (0.14); Difference: −0.15 (95%CI: −0.08 to 0.05).	ICER (cost per QALY gained):	Intervention is dominant	Bootstrapping (Y/N; iterations):	Y; 5000 replications.	Seems to be cost-effective but clinical study underpowered
		Intervention costs	Intervention: €2166.84 Control: €1969.39	QALYs gained at 15 months	Intervention: 0.83 (0.13); Control: 0.82 (0.12); Difference: 0.01 (−0.6 to 0.07). Not significant.			WTP Threshold(s)	€0 to €80,000	
								Probability cost-effective at WTP threshold(s):	At €16,000 WTP for a QALY, the probability of EXP treatment being cost-effective is 81%. At €80,000, the probability diminishes slightly, to 76%.	
Kemani et al. 2015 [62*]	CEA (collects SF-6D but not used to derive QALYs)	Total gross costs at post treatment, 3 months and 6 months	Intervention (ACT): $6219 (5392) post-treatment, $6339 (5090) at 3 months and $7836 (5676) at 6 months; Control (AR): $7584(5318) post-treatment, $6734(4437) at 3 months and $5694 (4713) at 6 months; There were no statistically significant differences in any of the cost domains between groups at pre-treatment, posttreatment, or follow-up (*p* > 0.05). Post treatment and 3 month follow up ACT is significantly cheaper but not at 6 month follow-up	Pain disability at pre-treatment, mid treatment, post treatment, 3 month and 6 month follow ups:	Intervention: 39.1 (14) *N* = 29 at pre-treatment, 31.6 (15.6) N = 23 mid-treatment, 28.8 (16.1) *N* = 24 post treatment, 28.5 (16.6) N = 23 at 3 months and 31.2 (19.0) N = 19 at 6 months; Control: 40.7 (14.1) N = 30 pre-treatment, 42.5 (14.6) N = 22 mid-treatment, 40.3 (13.6) *N* = 19 post treatment, 35.0 (18.8) *N* = 18 at 3 months and 34.0 (16.2) N = 18 at 6 months; Linear growth model testing for differential linear change between treatments, produced a Beta of −8.30, SE = 2.94, *p* < 0.01. ACT superior to AR in terms of improvements in disability Cohen’s d = 0.61 *p* < 0.01,at post-treatment but between post treatment and 6 month follow-up AR was superior to ACT (Beta 4.29 SE = 1.67,d = 0.63 *p* = 0.01).	ICER (cost per PDI change):	ACT was dominant at post-treatment, post assessment and at 3 month FU. At 6 month FU they report no significant differences in costs or effectiveness between the two conditions.	Bootstrapping (Y/N; iterations):	Y; 5000 replications.	ACT was more cost-effective than AR at post and 3-month follow-up assessment, but not at 6-month follow-up
		Intervention costs per participant	Intervention costs per participant were estimated to $2177 for ACT. Intervention costs $2148 for AR.	Number of individuals demonstrating clinically significant change (defined as an improvement of 1 SD) at post-treatment, 3 months and 6 months	Intervention: 5/24, 4/23, 4/19; Control: 0/19, 5/18, 2/18					
Norton 2015 (but some data taken from Lamb 2010) [83, 84]	CUA (Markov Model with 1 and 10 year time horizons) EQ-5D	Total costs at 1 year, 10 years	Intervention (CBT): $4779 at 1 year, $39,390 at 10 years; Control (active management - AM): $5091 at 1 year, $45,125 at 10 years; Difference: -$312 at one year and -$5735 at 10 years	EQ-5D data	Intervention: 59% improved at 1 year; Control: 31% improved at 1 year.	ICER (cost per QALY gained):	$7197 per QALY gained at one year; $5855 per QALY gained at ten years which is considered cost-effective.	Parameters varied:	Various scenario analyses exploring impact of changing relapse rate, utility values, volume of health services received, insurance plan, worst case	Cognitive Behavioural Therapy is cost-effective LBP care from the US commercial payer perspective
		Medical costs at 1 year, 10 years	Intervention: $4779 at 1 year, $39,390 at 10 years; Control: $5091 at 1 year, $45,125 at 10 years					Bootstrapping (Y/N; iterations):	Unclear (poorly reported), either bootstrapping or probabilistic sensitivity analysis; 5000 iterations.	
		Intervention costs per participant	Intervention (CBT plus AM): £187 (SE = 0.266). Control: £14.05					WTP Threshold(s)	$50,000 and $100,000 have been cited as benchmarks in the United States	
Herman (2017) [49]	CUA (SF-12/ SF-6D)	Healthcare (payer costs including out-patient care, emergency care, inpatient care, medicines, and imaging).	CBT $2760; MBSR $1283; UC $2265; CBT vs UC + $495 (−$2741, +$3550); MBSR vs UC -$982 (−$4108, +$1301); MBSR vs CBT -$1477 (−$4956, +$1017).	QALY gains at 1 year follow up compared to baseline 1 year before intervention	CBT: 0.765; MBSR 0.753; UC 0.728 CBV vs UC: + 0.041 (+ 0.015, + 0.067); MBSR vs UC: + 0.034 (+ 0.008, + 0.060).	ICER (cost per QALY gained):	CBT vs UC: $3049. MBSR dominated UC (lower cost, higher number of QALYs gained).	Bootstrapping (Y/N; iterations):	Bootstrapped ICERs(1000 replications) produced a cost-effectiveness plane.	In this setting CBT and MBSR have high probabilities of being cost-effective, and MBSR may be cost saving, as compared to UC for adults with CLBP. These findings suggest that MBSR, and to a lesser extent CBT, may provide cost-effective treatment for CLBP for payers and society
		Of which back pain related costs (back pain related code or pain related medication)	UC $699; CBT $1683; MBSR $572; CBT vs UC + 984 (−$1075, +$3385), MBSR vs UC -$127 (−$2670, +$942), MBSR vs CBT -$1111 (−$3662, +$488).					WTP Threshold(s)	$50,000/QALY	
		Societal costs including productivity losses	UC $6304, CBT $6428, MBSR $5580. CBT vs UC + $125 (−$4103, +$4347), MBSR vs UC -$724 (−$4386, +$2778), MBSR vs CBT -$849 (=$5338, +$2662).					Probability cost-effective at WTP threshold(s):	MBSR has a 90% probability of being cost-effective and CBT has an 81% probability.	
		Costs of therapist hours plus add-on costs.	Intervention costs for CBT and for MBSR were $150 per participant and $0 for UC.							
Bennell et al., 2016 [35*]	CUA (AQoL)	Treatment costs per participant	PCST & exercise: AU$1065 Exercise: AU$439; PCST: AU$730.	Overall average knee pain intensity in the past week (0-100 scale);	At week 12, no significant between-group differences for reductions in pain;	ICER (cost per QALY gained):	Trial showed a cost savings from combined treatment but a smaller gain in QALYs. Mean net benefit of $2600 Australian was not statistically significant			Combined psychological and exercise intervention was significantly more efficacious for improving physical function, but not pain, than either treatment alone; cost effectiveness was not demonstrated (net benefit approach).
				Physical function subscale of the Western Ontario and McMaster Universities Osteoarthritis Index (WOMAC);	PCST & exercise vs exercise alone: 3.7 units [95% CI 0.4, 7.0] PCST & exercise vs PCST alone: Significantly 7.9 units [95% CI 4.7, 11.2]. Significantly greater improvements. These differences persisted at 32 weeks for both comparisons and at 52 weeks compared to PCST alone (but not compared to exercise alone).					
				% reporting overall improvements (pain/function) at 52 weeks	PCST & exercise:78%; Exercise: 54%; PCST: 56%.					
				AQol-6D	There was no significant difference in QALYs over 52 weeks					
Hedman-Lagerlof et al. 2019 [17*]	CEA/CUA	Mean (SD) gross total costs post treatment:	Intervention (iEXP): $8903 (8123); Control (WLC): $11,940 (11,833); Intervention had significantly greater decrease in costs than control of $5097 (95%CI: − 9337 to − 857).	FIQ scores from baseline to post treatment	Intervention: 55.02 (16.78) to 36.44 (25.56); Control: 57.86 (15.76) to 57.51 (21.62)	ICER (per additional responder as measured by reliable change in FIQ):	For societal perspective, intervention dominant using FIQ. Healthcare only perspective ICER per 1 additional responder (reliable change in FIQ) was $2211.	Bootstrapping (Y/N; iterations):	Y; 5000 replications.	Study indicates that this treatment may be highly cost-effective.
		Mean (SD) direct medical costs	Intervention: $2847 (3729); Control: $2685 (3335); Difference (bootstrapped model): − 1445 (95%CI: − 3289 to + 400) not significant.	% of patients classified as treatment responders	Intervention: 44%; Control: 11% classified as treatment responders. Difference found to be significant in bootstrapped regression model (estimate = 0.33 (95% CI = 0.19 to 0.47), z = 4.66 *p* < 0.001.					
		Mean (SD) indirect medical costs	Intervention: $5283 (7086); Control: $9178 (11,651); Difference (bootstrapped model): Significantly lower in intervention group 4380 (95% CI:-8036 to −724).	Change in utility scores (EQ-5D) from baseline to post treatment	Intervention: 0.48 (0.3) to 0.6 (0.3). Control: 0.41 (0.32) to 0.44 (0.32).	ICER (cost per QALY gained):	For a societal perspective the intervention was dominant per QALY gained. Healthcare only perspective the ICER per QALY gained was $726/0.07 = $9734.	Probability cost-effective at WTP threshold(s):	Societal perspective: At $0 WTP, iExp had 100% probability of being cost-effective for FIQ and QALY outcomes. Health care perspective: The iExp had an 80% probability of being cost-effective given a WTP value of $2600 for FIQ and $21,500 for QALYs.	
		Intervention mean (SD) costs	Intervention: $726 (462); Control: N/A	Group x time interactions:	All secondary outcomes showed statistically significant group x time interactions favouring the intervention group.					
Luciano et al. 2014 [71*]	CEA (EQ-5D VAS)/CUA (EQ-5D)	Total costs per patient at baseline and follow up	Intervention 1 (CBT): €3098.80 to €1847; Intervention 2 (RPT): €2606.10 to €3663.70; Control: €2543.5 to €3123.70	Utility score (EQ-5D) at baseline and follow up	Intervention 1: 0.40(0.26) to 0.61 (0.25); Intervention 2: 0.40 (0.27) to 0.53 (0.27); Control: 0.38 (0.27) to 0.54 (0.28)	ICER (cost per change in EQ-5D VAS):	For societal perspective: CBT: Dominant against both TAU and RPT. RPT vs TAU: €53 per EQ-5D (VAS) change against TAU (ITT analysis). For healthcare perspective: CBT: Dominant against both TAU and RPT. RPT vs TAU: €63 per EQ-5D (VAS) change against TAU (ITT analysis).	Parameters varied:	Completers, ITT and per-protocol analyses at the 6-month follow-up for both healthcare and societal perspectives comparing CBT with RPT and TAU.	CBT is cost effective
		Direct costs from baseline to follow up	Intervention 1: €2200 to €1370; Intervention 2: €1864.3 to €2860; Control: €1772.30 to €2370.	EQ-5D VAS score from baseline to follow up	Intervention 1: 45.18 (16.98) to 59.62 (15.78); Intervention 2: 46.79 (15.48) to 57.3 (14.11); Control: 43.36 (14.5) to 52.86 (14.25); In a between group analysis, only the EQ VAS score was significantly different across groups. This analysis was conducted on completers only.			Bootstrapping (Y/N; iterations):	Y; 1000 replications	
		Indirect costs from baseline to follow up	Intervention 1: €916.30 to €476.80; Intervention 2: €741.80 to €803.00; Control: €771.20 to €750.90.	QALY gain at follow up	Intervention 1: 0.25 (0.12); Intervention 2: 0.23 (0.12); Control: 0.24 (0.13). The bootstrap analysis suggested that the increment effects of CBT compared to TAU on QALYs was not significant (i.e. the CI crossed zero) in the ITT sample. And that was the same for CBT compared to RPT.	ICER (cost per change in EQ-5D VAS):	For societal perspective: CBT: Dominant against both TAU and RPT. RPT vs TAU: €79,071 per EQ-5D (VAS) change against TAU (ITT analysis). For healthcare perspective: CBT: Dominant against both TAU and RPT. RPT vs TAU: €98,434 per EQ-5D (VAS) change against TAU (ITT analysis).	WTP Threshold(s)	€0 to €100,000	
		Cost of CBT intervention	€271.1.					Probability cost-effective at WTP threshold(s):	For the societal perspective NMB and 95% CIs for the CBT intervention are greater than zero at all hypothetical levels of WTP included. At a WTP of €40,000, RPT has a probability of only approximately 30% to be more cost-effective than TAU. For the healthcare perspective NMB CBT was dominant. For RPT in the ITT analysis ICERs were set to approximately €100,000, which is well above established cost-effectiveness thresholds.	
Luciano et al. 2017 [72]	CUA (EQ-5D)	Total overall costs mean (SD) at follow up	Intervention 1 (GACT): €2267.3 (1783.6); Intervention 2: (RPT): €2654.6 (2086.8); Control (WL): €4163.6 (3361.2); WL group had significantly higher costs than the ACT and RPT groups, which did not differ significantly from each other and this is the same when all costs are combined. Bootstrapping suggested ACT compared to WL saves between €1800 and €2000.	Utility score (EQ-5D) at baseline and follow up	Intervention 1 (GACT): 0.58 (0.17) to 0.8 (0.11); Intervention 2 (RPT): 0.57 (0.16) to 0.75 (0.15); Control: 0.54 (0.15) to 0.57 (0.16). At follow up the between group differences were overall significant (*P* < .05). With the exception of the comparison of GACT versus RPT, the other between group differences were statistically significant.	ICER (cost per change in EQ-5D VAS):	ACT was marginally more expensive than RPT but marginally more effective. Both ACT and RPT were superior (dominant) to WL control in all the plots they performed so ICERs not reported.	Parameters varied:	Three different samples analysed - completers, Intention to Treat sample, and a Per Protocol Analysis	Acceptance and commitment therapy appears to be a cost-effective treatment compared with RPT in patients with FM (but notes small sample sizes).
		Total direct costs mean (SD) at 6 month follow up	Intervention 1: €824.2 (1062.7). Intervention 2: €1730.7 (1656.8). Control: €2462.5 (2822.0); ACT group had significantly lower direct costs that the two control groups.							
		Total indirect costs mean (SD) at 6 month follow up	Intervention 1: €1443.1 (1363.9); Intervention 2: €924.0 (1440.0); Control: €1701.1 (1629.2).			ICER (cost per QALY gained):	Conclusions the same as for EQ-5D (VAS) i.e. both ACT and RPT are dominant against WL but ACT also dominant against RPT.	Bootstrapping (Y/N; iterations):	Y; 1000 replications.	
		Medication costs mean (SD) at 6 month follow up	Intervention 1: 0 (0); Intervention 2: 658.7 (363.9); Control: 320.8 (361.8)							
		Intervention costs mean (SD) at 6 month follow up	Intervention 1: €263.0 (27.5); Intervention 2: N/A; Control: N/A							
Luciano et al. 2013 [69*]	CUA	Overall total costs (Intervention, direct medical costs and indirect costs):	Intervention €1838.78 (2060.19); Control: €2201.56 (2032.33)	Change in FIQ between baseline and 12 months	Intervention: 58.90 (12.09) at baseline and 48.04 (18.27) at 12 months; Control: 55.97 (14.01) at baseline and 54.09 (15.14) at 12 months; F (ANCOVA) = 16.05 P < 0.001.	ICER (cost per QALY gained):	Psychoeducation dominated usual care	Parameters varied:	Completers, ITT and per-protocol analyses	A nonpharmacological intervention based on group psychoeducation is cost-effective compared with usual care alone in the context of primary care (Unclear as difference in costs was not significant).
		Direct costs from baseline to 12 month follow up	Intervention: €1366.73 (1259.63); Control: €1791.79 (1410.77); Difference: -€215.49 (CI −615.13 to + 287.71). Not significant	QALYs gained	Difference between groups = 0.12 (CI 0.06 to 0.19); Statistically significant			Bootstrapping (Y/N; iterations):	Y; 1000 replications.	
		Indirect costs from baseline to 12 month follow up	Intervention: €472.05 (1383.29); Control: €409.76; Difference -€197.32 (CI − 785.12 to + 395.74). Not significant					Probability cost-effective at WTP threshold(s):	At €0 WTP, probabilities of 85 and 74% of the psychoeducation intervention being cost-effective from the health care and societal perspective, respectively. At €3000, probabilities of 98 and 95% of the psychoeducation being more cost effective than usual care from the health care and societal perspective, respectively.	
		Cost of intervention (covered costs of medication, medical investigations, Primary Care services used, Secondary Care services used, and the cost of the treatment programme itself)	Intervention: €187.86 (75.41). Control: N/A							

One study examined psychoeducation with relaxation, two studies examined the cost effectiveness of exposure therapy, two studies examined Acceptance and Commitment Therapy and three studies examined Cognitive Behavioural Therapy (CBT). Two studies delivered the intervention via the internet, whilst the remainder delivered the intervention in a face-to-face, group format.

#### Non-specific chronic pain

In research carried out in the Netherlands by de Boer and colleagues [45*], 75 participants were recruited to a study comparing the outcomes and cost effectiveness of internet based CBT to group based, face-to-face, CBT. The drop-out rate was much higher in the internet group, although a greater proportion of participants in the internet group completed all of the course modules, compared to the face-to-face participants. A number of clinical outcomes, including the study’s primary outcome of pain catastrophising (PCS), showed greater improvement in the internet group compared to the face-to-face group among those who completed the intervention. However, this difference wasn’t present in the intention to treat analysis. Cost-effectiveness outcomes were equivocal and dependent on the sample analysed. Total costs (healthcare and social perspective) were €28 more in the internet group when the whole sample was examined. However, when the ICER was calculated, some patients were excluded because of missing effectiveness data. In this slightly different sample costs in the internet group were €199 lower, such that, when the ICER was calculated it favoured the internet group and suggested that for each PCS point that improved in the internet group, $40 was saved in costs.

A Swedish study, carried out by Kemani and colleagues [62*] compared the cost effectiveness of an Acceptance and Commitment Therapy (ACT) treatment, to an intervention consisting of applied relaxation (AR). Both interventions were delivered in groups and the effects on pain disability and healthcare and social costs were assessed immediately following treatment and at six-month follow-up. The results showed that ACT was superior over AR in reducing pain disability and ACT was also associated with lower costs. The investigators computed ICERs and, using 5000 boot-strapped replications, they plotted the results on a cost-effectiveness plane. Their analysis found that, immediately following treatment, 99% of the simulated ICERS were in the Southeast quadrant that favoured ACT over AR. At 3 month follow-up 78% of the simulated ICERs favoured ACT, but at 6 months the ICER plots were more centred and did not favour either treatment approach.

#### Chronic low Back pain

Goossens and colleagues [46], in a randomised controlled trial conducted in the Netherlands, compared the cost effectiveness of exposure in vivo to that of graded activity in 85 patients with low back pain in a high quality, randomised controlled trial. Sixty two patients provided data for the economic analyses. The analyses found that, in terms of quality of life outcomes (QALY’s derived from the SF-36), the exposure group appeared to do better but, in a simple comparison, the two treatments were not statistically different. However, over the year long follow-up period, the exposure group incurred fewer healthcare and social costs when compared to the graded activity group. Further analyses, including 5000 bootstrapped replications, found that the exposure treatment was more effective and resulted in a mean total cost saving of €2634 over the follow-up period. They also plotted the replications on a cost effectiveness plane which found that 49% of these fell in the South East quadrant, suggesting that the exposure treatment was dominant over graded activity. Furthermore, they reported the results from calculating a cost effectiveness acceptability curve which suggested that with a €16,000 willingness to pay for an additional QALY, the probability of the exposure treatment being cost effective is 81%.

Norton and colleagues [83*] published a re-analysis of data from an RCT of the cost-effectiveness of CBT for chronic low back pain conducted previously in the UK [84]. They constructed a number of models where they applied the likelihood of improvement and the utilities that were demonstrated in the UK study but with the costs of equivalent service use, estimated from United States commercial claims. The models were estimated over a ten year period with a variety of assumptions, such as a gradual loss of CBT knowledge and skills in the treated group and varying rates of back pain recurrence. Their estimates, which they found to be robust to varying assumptions, suggested that group based CBT was associated with an incremental cost-utility of $7197 per QALY in the first year, and $5855 per QALY over ten years.

Herman and colleagues [49] compared the cost-effectiveness of treatment with Mindfulness-Based Stress Reduction (MBSR) and Cognitive Behavioural Therapy (CBT), with usual care (UC), in adults with chronic low back pain. MBSR and CBT were associated with greater improvements in back pain and functional limitations as 26 weeks follow-up. MBSR reduced total societal costs by $724 per participant across 1 year versus UC, and reduced healthcare costs to the payer by $982 per participant. These cost savings came with a gain in QALYs of 0.034—an increase in HRQoL of approximately 5 % for the year. CBT was not found to be cost saving compared to UC, but was relatively inexpensive ($125 per participant to society and $495 to the payer) with slightly larger QALY gains (0.041). These findings suggest that MBSR may be a cost-effective treatment option for patients with chronic low back pain.

#### Knee osteoarthritis

Bennell and colleagues [35*], examined the cost-effectiveness of physical therapist delivered, Pain Coping Skills Training (PCST) and exercise, as part of a randomised controlled trial in 222 patients with chronic knee pain. The trial had three arms; PCST and exercise combined, PCST alone and exercise alone. PCST was reported as consisting of instruction in cognitive behavioural coping skills and the physical therapists underwent training that was delivered by two psychologists. Of the two primary outcomes, pain (VAS) in the past week did not differ between groups, but function was significantly improved in the combined treatment, when compared to the individual treatments alone. The authors also report that many of their secondary outcomes showed improvements favouring the combined treatment group. The combined treatment did not show a statistically significant cost saving in comparison to the two individual treatments and the authors concluded that cost-effectiveness was therefore not demonstrated.

#### Fibromyalgia

Hedman-Lagerlöf and colleagues [17*], in a randomised controlled trial, examined the cost effectiveness of internet delivered exposure therapy (iEXP) in 140 patients with Fibromyalgia in Sweden. The intervention aimed to encourage participants to restrain from using avoidant coping strategies and to approach situations that were normally avoided, despite pain. The intervention also included psycho-education and mindfulness components and comparisons were made with a waiting list control. Participants were followed up for a year and their healthcare and social costs were compared. The authors concluded that the intervention was highly effective (44% of the iEXP group were classified as having responded compared to 11% of the wait list control group) and was associated with significant cost savings. The authors concluded that even on a willingness to pay threshold of $0, the intervention was cost effective.

Luciano and colleagues [69*] examined the cost effectiveness of adding psychoeducational treatment to the usual care received by patients with Fibromyalgia in general practice in Spain. A total of 216 patients were randomised, with half receiving the psychoeducational treatment. The treatment was delivered in groups and consisted of five sessions of education and four sessions of relaxation training and the sample was followed up for 1 year. The analyses found that the psychoeducation group showed significantly greater improvements on the Fibromyalgia Impact Questionnaire and in terms of Quality of Life Years (as measured by the EQ5D). Direct medical costs and social costs were lower in the intervention group over the follow-up period, but not significantly so. A cost-utility plane was computed using one thousand bootstrapped replications and this showed that most of the replication points fell into the Southeast quadrant, suggesting that the intervention was dominant in cost-effectiveness terms. Using a willingness to pay threshold of zero, the probability that psychoeducation was more cost effective than usual care was 85% in regard to health care costs, and 74% in terms of social costs. Using a willingness to pay threshold of €3000, the same probabilities were 98 and 95%.

Luciano and colleagues [71*] examined the cost effectiveness of cognitive behavioural therapy (CBT) in comparison to a group receiving the US FDA recommended drug therapy and to a group who received usual care, in 168 primary care patients with Fibromyalgia in Spain. Self-reported medical and lost productivity costs over a 6 month follow-up period were significantly lower in the CBT group, compared to the other two groups. The CBT group also reported a higher quality of life, as measured by the EQ-5D, but these differences were only statistically significant using the visual analogue scale in the second part of the EQ-5D. The authors conducted a variety of analyses in the sample of 152 participants who completed treatment and all outcome measures. These analyses included calculating ICERs using the EQ-5D QALY score as well as the VAS score and using both healthcare costs and social costs. The point effectiveness ICER, as well as 1000 bootstrapped replications, found that CBT was dominant over recommended drug therapy and treatment as usual in all analyses, and this remained the case when the full intention-to-treat sample was included. The authors also computed net benefit curves and cost effectiveness acceptability curves, which supported the conclusions of the main analyses. The authors also noted that the net benefit estimate was greater than zero even when a UK willingness to pay threshold of £30,000 was considered.

A study with a similar design was conducted by Luciano and colleagues [72*] which examined the cost effectiveness of group Acceptance and Commitment Therapy (GACT), compared to recommended drug therapy and to a waiting list control group, in a sample of 156 patients with Fibromyalgia, recruited from primary care in Spain. Costs were measured using self-report questionnaires and effectiveness was measured using QALYs calculated from the EQ-5D-3L. In terms of the QALY outcomes at 6 months follow-up, the results for the GACT group were superior, but the differences were only statistically significant when compared to the waiting list control. Healthcare costs were significantly lower in the GACT group compared to the other two groups. As regards social costs (or indirect costs, as the authors call them) at follow-up, the waiting list control had significantly higher costs than the other two active treatment groups, whose costs were not significantly different from each other. The authors also computed ICERs, along with 1000 bootstrapped replications, and these showed that GACT was dominant, in terms of both healthcare and social costs, over the other two approaches, and this remained the case when the intention to treat sample was analysed. The authors also point out that GACT should be viewed as cost-effective even when considering a UK willingness to pay threshold of £30,000 per QALY.

#### Summary

The above nine studies of people with chronic pain suggest strongly that a range of psychological therapies are cost effective. Indeed, many produced at least as good outcomes as control conditions, but with cost savings. Nearly all of these studies were conducted in European countries, and therefore there must be caveats around the direct applicability of their findings to the UK and Scottish context. Despite this, the evidence suggests that investment in psychological therapies for chronic pain patients is likely bring a positive return in terms of patient outcomes and costs.

## Cancer

### Previous systematic reviews of economic evaluations of psychological interventions in cancer

A systematic review of economic evaluations of psychosocial interventions in cancer published up to 2015 [25] identified five studies which meet the criteria for this review (the interventions in the remaining three studies did not provide a psychologically informed intervention). The most recent of these [32*] will be discussed in more detail below. The overall conclusion of the review was that interventions based on cognitive behaviour therapy in particular had been demonstrated to represent good value for money in cancer care.

Three of the identified studies provide economic evaluations of cognitive behavioural based interventions (CBT) for patients with a range of cancer types. Bares et al. [108] compared one to one CBT to usual care for melanoma patients from a health care perspective, concluding this was cost effective for reducing distress. In a population with mixed cancers, Sabariego et al’s [109] study took a societal perspective, concluding that compared to non-directive group psychotherapy, a CBT based group intervention was dominant for both fear of cancer progressing and mental well being at 12 months. As detailed below, Arving et al’s [34*] cost utility analysis concluded that CBT based psychosocial support (provided on a one to one basis by either a trained nurse or psychologist) dominated usual care in terms of quality adjusted life years for participants with breast cancer. A cost utility analysis in which participants had mixed cancers [110] concluded that compared to usual care, a nurse delivered telephone intervention which comprised education, problem solving and communication, was also dominant in terms of the incremental cost effectiveness ratio. A further cost effectiveness analysis of a “supportive-expressive psychosocial group” for women with metastatic breast cancer, compared to usual care [111], concluded that this intervention achieved improvements in mood and pain at costs deemed acceptable compared with usual care.

Dieng et al. [25] highlight the overall paucity of full economic evaluations of psychosocial interventions for cancer patients and the variable methodological quality of the studies reviewed in their paper. While they conclude that the emerging evidence suggests that psychological interventions for cancer patients can be cost effective, particularly those which are CBT based, they call for further cost utility studies evaluating a boarder range of psychosocial interventions. They also highlight the need for transparency and consistency in reporting methods and findings.

### Overview of cancer studies in current review

Nine studies evaluating psychological interventions for cancer patients published after 2012 were identified which met the criteria for this review, including both cost effectiveness and cost utility analyses, some taking a healthcare perspective and others taking a broader societal cost perspective (see Tables 5 and 6). None have been undertaken in the UK (five were undertaken in northern European countries and the others were in the USA or Australia).

**Table 5:** Description of Studies: Cancer

Authors, Year	Condition	CEA	CUA	Setting	N (participants)	Baseline Characteristics	Intervention/ comparator(s)	Effectiveness measure(s); cost measures (price year)	Perspective	Time horizon
Arving et al. 2014 [34*]	Cancer (breast cancer)		✓	Sweden;	N Participants:	168	Individual (face to face or telephone) CBT based psychosocial support to breast cancer patients provided by: (1) oncology nurses or (2)psychologist. Participants received 0-23 sessions depending on needs / Usual care including visits with medical staff and referrals to psychiatrist or social worker for discussion	QALY EORTC-QLC-C30 mapped to utility scores;	Health care system	2 yrs.
				Hospital	Age in years (mean):	56		Euros (2012)		
					Sex (% female):	100				
					Ethnicity (% white):	Not given				
Chatterton et al. 2016 [41*]	Cancer		✓	Australia	N Participants:	109 (plus 89 carers)	(1) psychologist led 5 session CBT (2) Nurse led single session self-management intervention (resource kit sent to both groups)	AQOL-8D;	Health care costs	12mths
				Callers who called cancer helpline (included caregivers and patients)	Age in years (mean):	Not given		AUD 2011/12		
					Sex (% female):	82.5 (87.8 for carers)				
					Ethnicity (% white):	Not given				
Jansen et al. 2016 [58*]	Cancer (head and neck and lung cancer)		✓	Netherlands	N Participants:	156	Stepped care consisting of: watchful waiting; guided self-help via internet or booklet; face to face problem solving therapy; specialised psychological intervention and/ or psychotropic meds. Where HADS score remined > 7, progressed to next step. Comparator was care as usual. 75 allocated to intervention (75 watchful waiting; 50 guided self-help; 11 problem solving; 6 psychotherapy / medication). 81 allocated to control group (of these 20 received psychosocial care)	HADS, EQ-5D;	healthcare, indirect costs and productivity losses	12mths
				Hospital (patients with HNC or LC and scored > 4 on distress thermometer)	Age in years (mean):	62.0		Euros (2011)		
					Sex (% female):	39.1				
					Ethnicity (% white):	Not given				
Johannsen et al. 2017 [60*]	Cancer (breast cancer)	✓		Denmark	N Participants:	129	Manualised 8wk MBCT; 2 hr. weekly sessions of mindfulness practice, group discussion and cognitive exercises vs. Wait list control who only had contact to complete questionnaires	Pain intensity (0-10 point scale with MCID of 2 points);	Healthcare	6mths
				Hospital	Age in years (mean, SD):	Intervention: 56.8 (9.9); Control: 56.7 (8.1)		Euros (2015)		
					Sex (% female):	100				
					Ethnicity (% white):	Not given				
Lengacher et al. 2015 [67*]	Cancer (breast cancer)		✓	USA;	N Participants:	104	MBSR (for Breast Cancer) (6wks) conducted by trained psychologist vs. Usual care, standard post treatment visits MBSR = 47; UC = 49	QALYs (SF-12);	Healthcare and patient	12 weeks
				Hospital	Age in years (mean):	55		USD (Price year not stated)		
					Sex (% female):	100				
					Ethnicity (% white):	78.80%				
Mewes et al. 2015 [77*]	Cancer (breast cancer)	✓	✓	Netherlands;	N Participants:	422	Physical Exercise (12wk home based programme) delivered by physiotherapist; CBT (6 weekly sessions of 90 mins); Comparator: Usual care waiting list control.	QALYs (SF-36 converted to utilities);	Healthcare	5 yrs. (extrapolated from follow up)
				Hospital	Age in years (mean):	48.2		Euros (price year not stated)		
					Sex (% female):	100				
					Ethnicity (% white):	Not given				
Prioli et al. 2017 [87*]	Cancer (breast cancer)		✓	USA;	N Participants:	191	Mindfulness based Art Therapy (8 × 2.5 hr. sessions) or Breast Cancer support group (8 2.5 hr. sessions) with didactic lectures on breast cancer support topics with lectures and discussion, peer support; MBAT = 98 BCSG = 93	QALYs (SF-36 converted to utilities);	Healthcare	9wks
				Hospital	Age in years (mean):	56		USD (2011)		
					Sex (% female):	100				
					Ethnicity (% white):	58				
van der Spek et al. 2018 [97*]	Cancer		✓	Netherlands	N Participants:	170	1. Meaning centred group psychotherapy, 8 × 2 hr. weekly sessions manualised programme led by psychotherapist 2. Supportive group psychotherapy week social support group supervised by psychotherapist, 8 × 2 2 hr. sessions weekly 3. Care as usual, referred to GP if psychological help needed MCPG-CS = 57; SGP = 56; CAU = 57	QALYs (EQ-5D);	Healthcare	6mths
				Patients being treated for cancer with curative intent, expressing need for psychological support, University Medical Centre.	Age in years (mean):	57		Euros (2014)		
					Sex (% female):	70%				
					Ethnicity (% white):	Not given				
Zhang & Fu 2016 [106*]	Cancer (prostate cancer)		✓	USA;	N Participants:	267 (and 69 non-participating patients)	(1) biofeedback plus support (problem solving to teach symptom management skills) (2) biofeedback plus telephone support (3) usual care; also included feedback from eligible non-participating patients BF + group = 88; BF + phone = 86; UC = 93; INP = 69	QALYs (EQ-5D);	Societal: both healthcare costs and costs to patient	6mths
				Stage 2 prostate cancer patients with incontinence symptoms, Hospital	Age in years (mean):	65		USD (price year not stated)		
					Sex (% female):	0				
					Ethnicity (% white):	65.8

**Table 6:** Cost-effectiveness Outcomes: Cancer

Authors, Year	Type of Analysis/	Type of Costs	Costs	Type of effectiveness outcome	Effectiveness	ICER (definition);	ICER (results)	Sensitivity Analysis (definitions)	Sensitivity analysis (results)	Authors conclude (Reviewer comments where these differ from authors)
Arving 2014 [34*]	CUA	Total health care costs	Intervention (INS): €18,670; Intervention (IPS): €20,419; Control: €25,800	QALYs mapped from EORTC-QLC-C30	INS: 1.52; IPS:1.59; Control: 1.43; Not significantly different	ICER (cost per QALY gained):	Both INS & IPS dominated usual care. The differences between the INS and SC were estimated as of €-7130 (95% CI €-4286 to €-11,532) and between IPS and SC €-5381 (95% CI €-2732 to €-9524), respectively.	Parameters varied	Subgroup analysis namely: low and high tolerance levels re: QALY gain, tumour size, lymph node metastases, outliers in the number of intervention sessions, outliers in hospital activity. None except no regional lymph node metastases changed the conclusion.	Cost effective because the health care costs were lower and QALYs were higher compared to usual care alone (dominant).
		Cost of the intervention	€148 per session.	Mean (SD) utility value change over 2 year timeframe	INS: 0.26 (0.20); IPS:0.17 (0.26); Control: 0.20 (0.24)					
		Intervention costs	€500 (or 3%of the total costs).					Bootstrapping Y/N (iterations):	Y; 1000 replications	
Chatterton et al. 2016 [41*]	CUA	Intervention costs (high distress; low distress):	Intervention (psychologist led): $202; Control (nurse led): $60 Intervention: $181; Control: $60 Between group differences were significant at the 0.05 level.	QALYs derived from AQoL-8D (range depending on distress level)	Intervention: 0.614 to 0.760; Control: 0.577 to 0.744; Not significantly different. 0.037 (95% CI: 0.045 to 0.118) high distress and 0.016 (95% CI:0.027 to 0.060) low distress	ICER (cost per QALY gained):	Intervention dominates for high distress patients. ICER: AUD$*20,937.50* for low distress patients.	Bootstrapping Y/N (iterations):	Y, 50,000 iterations.	The height of the curve would need to be above 97.5% to be confident that the PI is a good value compared with the NI.
										PI is likely to be cost-effective compared with the NI for highly distressed cancer patients...conclusions for low-distress patients/carers support the use of the nurse-led self-management intervention
		Total costs (high distress; low distress):	Intervention (psychologist led): $3773, Control (nurse led): $4095; Intervention: $2729, Control: $2394 Between group differences were not significant at the 0.05 level.					WTP Threshold(s)	AUD$50000 per QALY was taken as the benchmark for cost-effectiveness in Australia	
		Mean additional cost of the intervention	Between $121 to $142 (depending on distress level).					Probability cost effective at WTP threshold:	81% at WTP threshold of AUD$50000 for high distress patients. 73% for low distress patients at the same threshold.	
Jansen et al. 2017 [58*]	CUA	Costs (base case):	Intervention: €9761; Control: €13,711; Difference − 3950 (95%CI: − 8158 to −190 P < 0.05);	QALYs gained (base case):	Intervention: 0.884; Control: 0.768; Difference: 0.116 (95%CI: 0.005 to 0.227 *P* < 0.05)	ICER (cost per QALY gained):	Intevention dominant as had higher QALY’s and statisically signficant lower cumulative costs.	Bootstrapping Y/N (iterations):	Y (5000 replications)	Stepped care likely to be cost effective
		Costs without productivity losses:	Intervention: €6287; Control: €9175; Difference: −2888 (95% CI: − 5630 to − 424 *P* < 0.05)	QALYs gained (without productivity losses):	Intervention: 0.885; Control: 0.767; Difference: 0.118 (95%CI: 0.009 to 0.227 P < 0.05)			Probability cost effective at WTP threshold:	96% of iterations in south-east quadrant of cost-effectiveness plane.	
Johannsen et al. 2017 [60*]	CEA	Average costs T1 to T4	Intervention: €1706; Control:€2436; Mean difference: €729, *p* = 0.07	n/N (%) achieving clinically relevant (2 point reduction in 0-10 scale) change in self-reported pain intensity	Intervention: 19/36 (52.8%); Control: 14/48 (29.2%); OR: 2.71 (higher odds of achieving MCID), *p* = 0.03	ICER (per additional MCID reduction in self-reported pain scale):	Intervention dominates as has lower costs and higher odds of achieving MCID	Bootstrapping Y/N (iterations):	Y; (1000 iterations).	Cost-effective as 2.71 higher odds of achieving minimal clinically important difference and lower cost
		Intervention cost	Intervention: €240 per MBCT participant Control: N/A					WTP Threshold(s)	€0, €1000	
								Probability cost effective at WTP threshold:	At €0 per additional participant meeting MCID, MBCT was cost-effective with a probability of 85%. At a WTP of €1000 per additional participant with MCID, MBCT was cost-effective with a probability of 90%.	
Lengacher et al. 2015 [67*]	CUA (SF-12)	Costs per participant (costs per session)	Intervention $666 ($111); Control: Not reported	QALY gain at 12 weeks	Intervention: 0.033; Control: 0.021; Incremental gain 0.03 (95%, confidence interval [CI] = 0.02-0.04).	ICER (cost per QALY gained	$22,200 (healthcare perspective); $19,733 (patient/out of pocket perspective)	Parameters varied	ICERs were calculated with the upper and lower bounds of the 95% CI for both costs and MBSR(BC) effects. Assumed effect is sustained over longer time horizon and explored impact on ICERs	Appears to provide for significantly improved HRQOL at a comparatively low cost (fairly reasonable conclusion although could have provided a better sensitivity analysis to confirm).
		Mean (SD) patient opportunity costs	$592 ($494)							
Mewes et al. 2015 [77*]	CEA and CUA (SF-36) using Markov model with hypothetical cohort of 1000 and time horizon of 5 years.	Total costs over 5 year period	Intervention 1 (CBT): €2983; Intervention 2 (PE): €2983; Control: €2798	Reduction in endocrine symptoms using FACT-ES:	Clinically relevant reduction in endocrine symptoms using (FACT-ES). The number needed to treat (NNT) was lower for CBT (5.53) than for PE (6.68).	Cost (per clinically relevant change in FACT-ES):	CBT: €1051, PE: €1315.	Parameters varied	PSA were propagated through the model using 5000 Monte Carlo simulations	In relative terms, CBT is likely the most cost-effective strategy compared to PE and control but results sensitive to uncertainties so overall cost-effectiveness uncertain.
		Intervention costs (including labour, training, admin, materials):	CBT: €190, PE:€197	Hot Flush Rating Scale (HFRS):	NNT to achieve a relevant improvement on Hot Flush Rating Scale (HFRS) was 5.61 for CBT, while PE was outperformed by the control.	Cost (per clinically relevant change in HFRS):	CBT: €1067, respectively PE: No clinically relevant difference seen between PE and the control.	WTP Threshold(s)	€20 k to €80 k, with €30 k per QALY commonly accepted as the prevailing ceiling ratio	
				Total QALY gain	CBT: 4.400; PE: 4.399; Control: 4.392	ICER (cost per QALY gained):	CBT: Incremental cost/QALY €22,502; PE: Incremental cost /QALY €28,087;	Probability cost effective at WTP threshold:	PE has the highest probability of being cost effective up to WTP values of €26,000/QALY above which CBT has the highest probability of being cost-effective, with a probability of 49% at a ceiling ratio of €30,000/QALY, up to 56% at €80,000/QALY	
Prioli et al. 2017 [87*]	CUA (SF-6D)	Cost per participant:	Intervention (MBAT): $992.49; Control (BCSG): $562.71 Difference between groups $429.79	Mean utility scores from baseline to 9 weeks	Intervention: + 0.05; Control: + 0.05	ICER (cost per QALY gained):	MBAT: $196,236 compared with baseline. BCSG: $128, 404 compared with baseline;	Parameters varied	Included cost components were varied. Yielded MBAT costs ranging from $241 to $792 (varying session leaders and art supply costs Other sensitivity analyses suggested that if if the session leader cost is less than $550, MBAT can be less costly than a BCSG.	An MBAT intervention is more costly than usual support group care and has a similar effect on utility as a BCSG (i.e. not likely to be cost effective).
		Intervention cost included screening, labour, materials, staff travel costs & those of participants (varied in sensitivity analysis):		QALY gain	Intervention: 0.00433; Control: 0.00433					
van der Spek et al. 2018 [97*]	CUA (EQ-5D)	Mean (SE) costs:	Intervention 1 (MCGP-CS): €4492 (778); Intervention 2 (SGP): €4545 (580); Control: €5304 (722); Incremental costs of MCGP-CS vs control: € − 812 (95% CI, − 2830 to 1350). Incremental costs of SGP vs control: € − 759 (− 2625 to 972).	Mean (SE) change in utility score:	MCGP-CS: 0.540 (0.016). SGP: 0.511 (0.014); Control: 0.507 (0.014); Difference between MCGP-CS vs control: 0.033 (95%CI:− 0.007 to 0.074); Difference between SGP vs control: 0.004 (95%CI:− 0.036 to 0.044).	ICER (cost per QALY gained):	MCGP was dominant (lower costs and more QALYs gained).	Parameters varied	Complete case analysis and costs/effects at different time point.	MCGP-CS is highly likely a cost-effective intervention (likely but there is considerable uncertainty and the sensitivity analysis could have explored this in more detail).
		Intervention costs per patient:	MCGP-CS: €288; SGP: €286; Control: N/A					WTP Threshold(s)	€0 to €30,000.	
								Probability cost effective at WTP threshold:	At €0, MCGP-CS has a 78% probability of being cost-effective compared to CAU, increasing to 85% at €10,000 and to 92% at €30,000. At €0 SGP has an 80% probability of being cost-effective compared to CAU, this does not increase if society is willing to pay more. Compared to SGP, MCGP-CS has a 52% probability of being cost-effective at €0, increasing to 63% at €10,000 and to 77% at €30,000.	
Zhang & Fu 2016 [106*]	CUA (EQ-5D)	Total cost (societal):	Intervention 1 (BP + Group) vs INP (non-participating group): $923.90; Intervention 2 (BP+ phone) vs INP $661.90; No statistically significant differences between groups on productivity cost	Incremental change in EQ-5D score	BF + group vs INP = 0.054 p < 0.05; BF + group vs control (usual care) 0.008 (95% CI:0.041, 0.058) *p* = 0.74 BF + phone = 0.057. p < 0.05 BF + phone versus control (usual care) 0.016 (95% CI: 0.033, 0.065) *p* = 0.53; Results are significant compared to non-participating group but not usual care.	ICER (cost per QALY gained):	Provider and patient ICERs were $16,759 and $12,561/QALY for support and telephone groups respectively. (Societal) ICERs compared with non-participating group was $17,276 for BF + group and $11,612 for BF + phone. No further analysis against usual care as results were not significantly different for this group.	WTP Threshold(s)	$50,000/QALY, the consensus threshold to determine cost-effectiveness for society.	The interventions of BF+ group or BF + phone were cost-effective compared with those of patients who were eligible but declined (INP group) participation (really depends on INP group motivations).
		Total cost (provider):	BP + Group vs INP: $410.40; BP+ phone vs INP $563.20; No statistically significant differences between groups on healthcare utilization cost							
		Total cost (patient)	BP + Group vs INP: $494.60,; BP+ phone vs INP $153.10; No statistically significant differences between groups on patient out-of-pocket expense							
		Incremental intervention cost per patient (provider perspective) compared with control (non-participating) group	BF+ group = $252; BF+ phone = $484							
		Incremental intervention cost (patient) compared with control (non-participating) group	BF + group = $564; BF + phone = $203							

## Breast cancer

Four of the studies are economic evaluations of psychological interventions for patients with breast cancer. Arving et al. [32*] report a cost utility analysis of individual CBT based psychosocial support for breast cancer patients provided by either a specially trained nurse or a psychologist, compared with standard care. This Swedish study took a health care system perspective and concluded that both psychological interventions dominated usual care, with lower health care costs and higher QALY’s (1.43 QALY for standard care compared to 1.52 QALY for nurse delivered psychosocial support and 1.59 QALY for psychologist delivered). The main driver of higher costs was in-hospital care.

Two economic evaluations focussed on mindfulness based group interventions for patients with breast cancer. Lengacher et al. [67*] compared a 6 –week mindfulness based stress reduction (MBSR) programme to usual care with respect to post cancer treatment symptoms and health related quality of life from a healthcare and patient perspective in a US based study. While the QALY increment of 0.03 achieved using MBSR was relatively costly if the benefits are assumed to last only the 12 week assessment of the study, if participants are likely to survive for 5-20 years and sustain the benefits of MBSR, the relative costs per QALY decline markedly over time. The authors conclude the intervention provides significantly improved health related quality of life at comparatively low cost. In a second American study which also takes a healthcare perspective, Prioli et al. [87*] evaluated the direct costs and effectiveness of mindfulness based art therapy (MBAT) compared with the effectiveness of a breast cancer support group. The MBAT intervention cost $429 more per participant than the usual support group care (both delivered over 8 weeks) and had a similar effect on utility based on a standardised quality of life questionnaire (SF-36), so was not likely to be cost-effective. In the parent RCT it was found that MBAT participants who had high stress levels at baseline experienced greater reduction in stress than the breast cancer support group participants at 9 weeks. The authors suggest that further sub-analysis according to baseline stress levels might be useful, as well as longer term data.

Two breast cancer based evaluations focussed on specific physical symptoms. Johannsen et al. [60*] concluded that an 8 week mindfulness based cognitive therapy group is a cost effective intervention for reducing pain intensity in women treated for breast cancer with persistent pain, although this Danish study did not include utility measures or indirect costs. The intervention cost per participant was €240 while the average total cost for the duration of the study was €1706 for the MBCT group compared with €2436 in the control group. Mewes et al. [77*] performed a cost effectiveness analysis from a health care system perspective of CBT and physical exercise for alleviating treatment induced menopausal symptoms in breast cancer patients, compared to a waiting list control group in a Dutch study, using a Markov model. They concluded that 6 weeks of group CBT is likely to be the most cost effective strategy for alleviating such symptoms, followed by a 12 week home based physical exercise programme, although the results were sensitive to uncertainties so the overall cost effectiveness was not certain. Incremental cost utility ratios were €22,502/ QALY for CBT and €28,078/QALY for physical exercise. Outcomes were influenced by the duration of the treatment effect, with shorter effect duration resulting in lower cost effectiveness. Compliance in the parent RCT was also relatively low. The authors suggest that a more targeted approach taking into account level of need or patient preferences, may increase compliance, improve outcomes and increase cost effectiveness.

Other economic evaluations with patients with different cancers have evaluated interventions more closely matched to distress. Chatterton et al. [41*] conducted their economic evaluation from a healthcare perspective, alongside a randomised trial of highly distressed cancer patients and carers calling help lines in Australia. The intervention was five sessions of individual telephone based CBT delivered by a psychologist, with a comparison group receiving a single telephone support session with a nurse counsellor. No significant differences were found in overall total costs or QALYs between intervention groups. However, using bootstrapped data, the psychological intervention was probably more cost effective than the nurse led intervention for high distress participants. For carers and patients at with high distress at baseline, the CBT intervention delivered slightly more QALYs (mean difference of 0.037) at a lower total cost. The authors were cautious in interpreting their findings, noting that the study was underpowered and differences did not reach statistical significance, however, conclude that more intensive psychological interventions for patients with greater levels of distress appears warranted.

Another Dutch cost utility evaluation targeting participants with mixed cancers, all of whom expressed a need for psychosocial support and at least one psychosocial complaint (e.g. depressed mood, anxiety) is reported by van der Spek et al. [97*]. Participants had been treated for cancer with curative intent within the last 5 years, and completed their main treatment. This evaluation compared an 8 session meaning centred group psychotherapy intervention for cancer survivors (MCPG-CS) with an 8 week social support group and care as usual, from a healthcare perspective. Mean total costs ranged from € 4492 (MCPG-CS) to €5304 (care as usual) while mean QALYs ranged from 0.540 (MCGP-CS) to 0.507 (care as usual). Meaning centred group psychotherapy was highly likely to be cost effective compared with both control groups; it was more effective and less costly compared with care as usual, and probably more effective but not less costly than the social support intervention, although differences did not reach statistical significance. The authors note that these findings contrast with those of Lemieux et al. [111], one of the studies included in the systematic review above, who did not find evidence of lower costs in the intervention group. The group psychotherapy intervention was similar in both studies, however, the earlier study was narrower in the scope of medical costs included and targeted advanced cancer patients, rather than those who have completed treatments intended to cure their cancer but reported psychological difficulties, as targeted by van der Spek at al [97*].

Jansen et al. [58*] evaluated the cost utility of stepped care targeting psychological distress in patients with head and neck cancer or lung cancer, an approach which has achieved good clinical outcomes with this population. In the stepped care programme the least resource intensive intervention is delivered to patient first, followed where necessary, by more resource intensive interventions. In this Danish study, the four steps were watchful waiting for 2 weeks; guided self-help; face to face problem solving therapy and CBT and / or psychotropic medication. The comparator was care as usual and the perspective was societal (including healthcare, indirect costs and productivity losses). Stepped care was found highly likely to be cost effective compared with care as usual; with the mean number of QALYs was 0.116 higher and the mean cumulative costs €3950 lower in the intervention group compared with the control group. The findings echo those of Chatterton et al. [41*], in which participants with increased levels of distress were also targeted. The larger cost–benefit difference reported here may reflect the design of the stepped care intervention where a minority of participants go on to receive the more resource intensive interventions. The two studies also differ as Chatterton et al. [41*] took a narrower healthcare perspective.

Zhang and Fu [106*] targeted prostate cancer patients with persistent urinary incontinence in the cost utility evaluation, however, alongside the three study groups from the original US-based RCT (biofeedback plus problem solving therapy delivered in a group or by phone, and care as usual), these authors also included eligible patients who declined the intervention study but agreed to provide feedback in their analysis. The authors argue that non-participants experience a greater economic and healthcare burden, choosing not to take part in a behavioural study out of economic concerns, and they may endure higher costs and lower quality of life in the long term than those who do choose to take part. The study interventions were found to provide meaningful outcome improvement at low cost, and to be to be cost effective in consideration of eligible patients who declined the interventions (but not the usual care group). The final ICERs per QALY were $17,276 for biofeedback plus group intervention and $11,612 for biofeedback plus phone intervention when compared with the intervention non-participating group. The authors acknowledge that the sample size is small, yielding limited statistical power to discern differences in cost effectiveness between the study groups. However, they also argue that the inclusion of indirect as well as direct costs provides information about the intervention’s benefits in a real world context that also encompasses non-participating eligible patients.

### Summary

Most of the studies outlined have limitations, in particular, limited sample sizes resulting in the tendency for the analyses to be underpowered and therefore fewer statistically significant differences in cost were able to be demonstrated. In addition, there was a range of cost perspectives, approaches to gathering cost data (e.g. hospital data sets, self-report), time frames, different populations of cancer patients, countries in which studies were based and approaches to handling uncertainty and missing data. This makes direct comparison difficult and conclusions are therefore more tentative. Looking at economic evaluations published since 2012 overall, there appears to be growing evidence for the cost effectiveness of CBT based interventions for a range of cancer patients. Whilst less strong, there is also some evidence for the cost effectiveness of mindfulness based approaches. The strongest evidence appears to come from studies in which psychological interventions targeted those with the most severe psychological distress, or where a stepped model of intervention, in which more resource intensive interventions are focussed on those with the most severe levels of distress, were offered. Whilst generally supporting the cost effectiveness of structured psychological interventions delivered in either groups or individually, it seems appropriate for policy makers to support targeted psychological interventions with cancer patients according to distress level in order to achieve the maximum cost effectiveness.

## Diabetes

### Previous systematic reviews of economic evaluations of psychological interventions in diabetes

A summary of the findings on the cost effectiveness of psychological interventions in treating depression in diabetes between 2000 and 2012 were described in a systematic review by Jeeva et al. [26]. Out of 1516 papers screened only 4 economic evaluations were identified and all were based in the US. These studies evaluated collaborative care programmes which included a case manager and stepped care treatments for depression involving psychological interventions and/or antidepressants. Two studies included problem solving therapy and/or antidepressants [112, 113], one included problem solving and behavioural activation or antidepressants [114] and one offered behavioural interventions [115]. The studies involved found that the interventions reduced depression, improved health status and were cost-effective compared to usual care. They found limitations of the studies included but it is hard to determine the impact of these limitations as no analysis was done.

Simon [113] presented a cost effectiveness analysis from a payer perspective over 24mths. They reported the collaborative care intervention as dominant with net savings from the intervention and increase in depression free days. Katon et al. [114] described a cost utility analysis of collaborative care from a societal viewpoint over 24mths. This intervention also dominated usual care with greater cost savings and gains in patient free days and QALYs. Hay et al. [112] and Katon et al. [115] involved a cost utility analysis from a payer viewpoint over 18 and 24mths. Both these studies reported costs per QALY gained within the usual willingness to pay parameters.

The limitations of sample sizes in accurately determining the cost-effectiveness of the interventions or differences in costs or health benefits are noted. It is suggested that the extent to which QALYs (and measures to estimate QALYs) are relevant in patients with diabetes and mental health problems is explored and that studies evaluate the key attributes of health from the patient’s perspective. The authors conclude that the economic evidence, from a U.S. payers perspective, suggests that collaborative care (with psychological interventions) in managing depression in people with diabetes results in health gains and may be cost saving.

### Overview of diabetes studies in current review

The current search identified 3 further economic evaluations of psychological interventions in diabetes (see Tables 7 and 8). Two of the studies were conducted in the UK and one in Germany.

**Table 7:** Description of Studies: Diabetes

Authors, Year	Condition	CEA	CUA	Setting	N (participants)	Baseline Characteristics	Intervention/ comparator(s)	Effectiveness measure(s); cost measures (price year)	Perspective	Time horizon
Camacho et al. 2016 [39*]	CVD/Diabetes		✓	UK	N Participants:	387	Low intensity CBT techniques delivered by Psychological wellbeing practitioners (mean 4.4 sessions) and collaborative care with GPs or practice nurses vs standard care with GP/Practice nurse. CBT: 191 SC: 196	QALYs; EQ-5D-5L	Healthcare (social care data had too many missing items)	24mths
				NHS Primary Care (North East England)	Age in years (mean, SD):	58.5 (11.7);		GBP (2014-15)		
					Sex (% female):	38				
					Ethnicity (% white):	86				
Ismail et al. 2018 [57*]	Diabetes; type II patients	✓	✓	UK (England);	N Participants:	334	Practice nurses delivered psychological skills (six techniques -from MI/CBT- health beliefs) vs standard care of self-management education and monitoring Both interventions included 12x30min sessions. D6: 170 SC: 164	HBA1c and QALYs (SF-12);	Health and social care	18mths
				Primary Care	Age in years (mean, SD):	Diabetes-6: 59 (11.1)SC: 58.9 (11.4)		GBP (2011-12)		
					Sex (% female):	Diabetes-6: 50SC: 52.4				
					Ethnicity (% white):	Diabetes-6: 36.8 SC: 43.8				
Nobis et al. 2018 [81*]	Diabetes	✓	✓	Germany; community setting, recruitment via advert and health insurance	N Participants:	260	Internet-based guided self-help for depression in diabetes based on CBT (6 sessions with a coach responding to homework) vs an internet-based psychoeducation session (no coach) GSH: 129 SH: 131	EQ-5D-3L	Health care and societal	6mths
					Age in years (mean, range):	51 years, range 18–79),		Euros (2013)		
					Sex (% female):	63				
					Ethnicity (% white):	74

**Table 8:** Cost-effectiveness Outcomes: Diabetes

Authors, Year	Type of Analysis	Type of Costs	Costs	Type of effectiveness outcome	Effectiveness	ICER	Sensitivity Analysis Used	WTP Threshold(s) (CEAC range)	Probability Cost Effective at Threshold(s)	Authors conclude (Reviewer comments where these differ from authors)
Camacho et al. 2016 [39*]	CUA (Markov model) extrapolated from trial data	Mean (unadjusted costs)	Intervention: £1896 (95% CI 1468 to 2224); Control: £1515, (95% CI 1205 to 1826)	Mean depressions score at follow up	0.23 points lower (95% confidence interval − 0.41 to −0.05) in participants who received collaborative care compared with those who received usual care.	ICER (cost per QALY gained - model-based):	£16,123	Parameters varied in sensitivity analysis	Time horizon, excluding training costs, excluding deaths, change to waning of treatment benefit over time, discount rate.	Collaborative care may also be cost-effective in the English health service for patient groups with depression in conjunction with long-term physical health conditions, and over a long-term time horizon. However, the long-term findings were extrapolated from 4-month trial data and so associated with some uncertainty
		Healthcare usage costs (net cost for collaborative care compared with control)	£674 (95% CI −30,953 to 38,853)	Net QALY gain	0.04 (95% CI −0.46 to 0.54); No significant differences between groups for disability, self efficacy, illness perceptions, and global quality of life or for disease specific quality of life	ICER (cost per QALY gained) within-trial data:	£29,132	WTP Threshold(s)	£15,000, £20,000, £60,000	
		Intervention costs (including PWP training, clinical and admin time and supervision costs)	Intervention: £318; Control: N/A					Probability treatment is cost effective at WTP threshold(s)	Model-based: 0.53 at £15,000, 0.54 at £20,000, 0.56 at £60,000. Within-trial analysis: 0.49 at £20,000.	
Ismail et al. 2018 [57*]	CEA (point improvement in HbA1c) and CUA (SF-12)	Adjusted mean difference in total health & social care costs at 18 months (including intervention costs and discounting non-intervention costs):	£150 (95% CI = −34 to 333)	Mean difference in HbA1c	−0.79 mmol/mol (95% confidence interval CI = −5.75 to 4.18). No significant difference between intervention and standard care	ICER (cost per unit change in HbA1c):	Not reported. Cost effectiveness plane shown in supplementary files.	WTP threshold(s)	£0 to £50,000	Unlikely to be cost-effective
		Mean difference in intervention costs	£276 (95% CI = 225 to 327)	SF-12	No significant difference between intervention and standard care for any of the secondary outcomes	ICER (cost per QALY gained):	Not reported. Cost effectiveness plane shown in supplementary files.	Probability treatment is cost effective at WTP threshold(s)	5% at £0 WTP, 65% at £5000 and at £50,000 (HbA1c); Did not exceed 35% at any WTP threshold (QALYs).	
Nobis et al., 2018 [81*]	EQ-5D-3L	Total costs	Intervention: €5195; Control: €5098; Mean costs were therefore €97 higher in the intervention group than in the control group after 6 months.	% showing treatment response at six months	Intervention: 77 (60%) Control: 23 (18%)	ICER (cost per treatment response):	€233	Bootstrapping (Y/N; replications):	Y (2500 replications)	Demonstrated a high probability of being cost-effective compared with an active control group.
		Intervention costs	Intervention: €283.46; Control: €33.10	QALYs gained at six months	Intervention: 0.33 (s.d. = 0.11) Control: 0.32 (s.d. = 0.11) No significant differences were found between the groups (*p* = 0.51) at six months	ICER (cost per QALY gained):	€ 10,708	WTP threshold(s)	€5000, €14,000	
								Probability treatment is cost effective at WTP threshold(s)	97% at €5000 (treatment response), 51% at €14,000 (per QALY gained).	

Nobis et al. [81*] conducted cost and utility analysis alongside a German trial of 260 diabetes patients receiving web based treatment of depression in diabetes, using coaches to guide the intervention, or an active control of web based psychoeducation. The study was rated as acceptable in quality and took an inclusive perspective of societal costs (direct and non-direct medical care, productivity, opportunity costs and domestic assistance costs). They conclude that the intervention had a high probability of being cost and utility effective compared to an active control group from a societal perspective at a willingness-to-pay ceiling of €5000 for a treatment response. The authors noted the limited power to conduct an economic evaluation but this was balanced by comprehensive sensitivity analyses. They also note the short follow up period of 6mths and exclusion of cost of diabetes medication (due to differences in costs between treatments for Type 1 and Type 2 diabetes). They discuss the differences in findings between high rates of treatment response and non significant changes in QALYs, citing literature suggesting that the EQ-5D may not be a sensitive measure in patients with mental health problems.

Camacho et al. [39*] reviewed the long term cost effectiveness of collaborative care for people with diabetes or cardiovascular disease. This UK based study compared usual care with psychological interventions delivered by a Psychological wellbeing Practitioner as part of collaborative care. The controlled trial on which the study was based [40] was rated as acceptable quality. A Markov model was used to extrapolate the long term cost effectiveness (2 yrs) from data at 4mths. The authors concluded that collaborative care had the potential to be a cost effective intervention but that conclusions were extrapolated from a short term follow up with notable missing data and are therefore subject to some uncertainty.

The third diabetes paper was also set in primary care and involved practice nurses trained in six skills from cognitive behaviour therapy and motivational interviewing. In both arms of the trial twelve 30 minute sessions were offered over a year for patients who had suboptimal glycaemic control. The primary outcome was change in HbA1c and secondary measures included change in weight, depressive symptoms and diabetes related distress. There was no significant change in any of the outcome measures at 18mths and the intervention was unlikely to be cost effective. The authors conclude that training practice nurses in MI and basic CBT did not lead to improvements in glycaemic control and was unlikely to be cost effective. The increased contact in the control arm with standard care nurses also did not increase control.

### Summary

There are relatively few papers evaluating the cost-effectiveness of psychological interventions or therapies in diabetes. Limitations within the current papers means that it is difficult to draw conclusions about the cost-effectiveness of psychological interventions in diabetes care and further research with larger sample sizes is required.

## Multiple sclerosis

No systematic reviews of the cost effectiveness of psychological interventions in multiple sclerosis were identified.

### Overview of multiple sclerosis studies in current review

There were four primary research studies focusing on patients with MS, included (see Tables 9 and 10). All studies were conducted in the UK and used the EQ-5D to estimate QALYs. Three used the General Health Questionnaire as a disease specific outcome measure. Two studies evaluate the cost-effectiveness of group delivery in supporting adjustment to MS or MS symptoms [52, 55]. Two newer studies test other methods of delivery – Skype [36*] and nurse delivery of cognitive behavioural skills or supportive listening via meetings and telephone sessions [79*]. The interventions are clinically effective but the cost-effectiveness results are variable with some indication of better cost-effectiveness for those that are more distressed or depressed.

**Table 9:** Description of Studies: Multiple Sclerosis

Authors, Year	Condition	CEA	CUA	Setting	N (participants)	Baseline Characteristics	Intervention/ comparator(s)	Effectiveness measure(s); cost measures (price year)	Perspective	Time horizon
Bogosian et al. 2015 [36*]	MS	✓	✓	UK;	N Participants:	40	Manualised mindfulness groups based on MBCT 8x1hr sessions delivered via Skype, run by health psychologist who had completed mindfulness teacher training vs waiting list control. Mindfulness: 19 WL: 21	QALYs (EQ-5D);	Health and social care, patient (informal care)	3mths
				Primary and hospital care patients recruited via NHS and MS charity	Age in years (mean, SD):	Mindfulness: 53.4 (8.3) WL: 50.9 (9.9)		GBP (2012-13)		
					Sex (% female):	Mindfulness: 47.4 WL: 61.9				
					Ethnicity (% white):	Mindfulness: 89.5 WL: 90.5				
Humphreys et al. 2013 [52*]	MS	✓	✓	UK;	N Participants:	151	Adjustment group (6wk x3hrs) run by assistant and clinical psychologist vs standard care (no psychological interventions). Group: 72 SC: 79	HADS, QALYs (EQ-5D);	Healthcare	8mths
				Hospital based	Age in years (mean, SD):	Intervention 44.5 (11.1); Control: 47.5 (10.5)		GBP 2009		
					Sex (% female):	Intervention: 75; Control: 70				
					Ethnicity (% white):	Not given				
Mosweu et al. 2017 [79*]	MS	✓	✓	UK;	N Participants:	94	8 sessions nurse delivered CBT (2 F2F and 6 telephone) versus supportive listening (SL) CBT: 48 SL: 46	QALYs (EQ-5D-3L); GHQ-12 score;	Health social and indirect care perspectives	12mths
				Hospital based (multi-centre)	Age in years (mean):	CBT: 40; SL: 43		GBP (2008/09)		
					Sex (% female):	CBT: 73 SL: 65				
					Ethnicity (% white):	CBT: 79; SL: 72				
Thomas et al. 2013 [55*, 56]	MS	✓	✓	UK: 3 sites in southwest England, recruitment via primary, secondary care and MS Society	N Participants:	164	Manualised group programme (FACETS) based on cognitive behavioural, energy management and self efficacy theories. 6x90min sessions delivered by MDT staff vs current local practice alone (CLP) e.g. general fatigue and MS management advice. FACETS: 84 CLP: 80	QALYs (EQ-5D), SF-6D	Health and social care	4mths
					Age in years (mean, SD):	FACETS: 48 (10.2) CLP: 50.1 (9.1)		GBP (2010)		
					Sex (% female):	73				
					Ethnicity (% white):	FACETS: 94 CLP: 99

**Table 10:** Cost-effectiveness Outcomes: Multiple Sclerosis

Authors, Year	Type of Analysis/	Type of Costs	Costs	Type of effectiveness outcome	Effectiveness	ICER (definition):	ICER (result)	Sensitivity Analysis Used	Sensitivity analysis result:	Authors conclude (Reviewer comments where these differ from authors)
Bogosian et al. 2015 [36*]	CEA and CUA	Total costs at baseline	Intervention: £3080; Control: £3703	Mean GHQ score (SD) at baseline, end and follow-up	Intervention: 16.10 (6.35) at baseline, 11.43 (4.55) at end, 9.33 (5.02) at follow-up; GHQ changed 17.29 (4.89) at baseline, 14.87 (5.94) at end, 15.17 (4.42) at follow-up; Mean GHQ scores were lower (better) in the mindfulness group compared to the waiting-list group at both the post-intervention and three-month follow-up.	ICER (cost per change in GHQ score)	Intervention dominates	Bootstrapping (Y/N; replications):	Y (1000 iterations).	Skype intervention likely to be cost effective in terms service costs
		Mean difference in total costs at 20 week follow up	–£2285 (95% CI − 5003 to 579); Not statistically significant	Mean difference in QALYs (adjusted for baseline scores) as measured by EQ-5D	−0.006 (95% CI − 0.039 to 0.027). No significant differences in QALYs between the groups.	ICER (cost per QALY gained)	Unclear (intervention either dominates or is extendedly dominated).	Probability treatment is cost effective at WTP threshold(s)	87.4% probability intervention “saves money and improves outcomes” (no WTP reported).	
		Mean difference in health and social care costs at 20 week follow up	−£720 (95% confidence interval (CI) –£2636 to £1196)					Probability treatment is cost effective at WTP threshold(s)	90% chance mindfulness is most cost-effective option at a threshold of £20,000, although many iterations lie within south west quadrant.	
		Informal care costs	Higher for the waiting list group.							
Humphreys et al. 2013 [52*]	CEA (CUA not performed as no between group significant difference in EQ-5D at any time point)	Costs per patient over 8 month follow up:	Intervention: -£378 per respondent Control: £ + 297 per patient Mean reduction in costs between intervention and control: −£401	Mean (SD) BDI	Intervention: −2.38 (4.72); Control: −0.67 (3.44); Statistically significant difference (p = 0.01) in the point reduction in the BDI between the intervention and control group over eight months (mean difference − 1.70, 95% confidence interval − 3 to − 0.4 using Levene’s test for equality).	ICER (cost per additional point reduction in BDI score)	The adjustment group was associated with an incremental cost effectiveness ratio of £118 per additional point reduction in BDI score.	Bootstrapping (Y/N; replications):	Bootstrapping (1000 replications)	Cost-effective in the short term (depends on WTP for a change in BDI score)
		Intervention costs (added to medication and recourse use, components included salary of those involved in group and room costs)	Intervention: £248 per participant; Control: N/A	Mean (SD) utility score on EQ-5D	Intervention: 0.53 (0.30); Control: 0.53 (0.28); Differences between the groups were not statistically significant at any time point			Probability treatment is cost effective at WTP threshold(s)	a 93% probability that the adjustment group will be considered cost effective if purchasers are willing to pay up to £118 per point reduction in BDI score.	
Mosweu et al. 2017 [79*]	CUA	Mean costs at follow up (health and social care perspective):	Intervention: £7331; Control: £5026; Mean difference (when adjusted for baseline costs) was not statistically significant (bootstrapped 95% CI, −£187 to 3771)	Mean improvement in GHQ-12 score	Intervention: 2.69; Control: 1.97; Difference (1.9572) was statistically significant (bootstrapped 95% CI −5.41 to −1.05)	ICER (cost per improvement in GHQ-12 score)	£821 (health and social care perspective), £1242 (societal perspective).	WTP Threshold	Using a £20,000 per QALY gained threshold	Not cost-effective.
		Difference in mean costs (societal perspective)	£2871; Not statistically significant (bootstrapped 95%CI: −£2028 to £7793)	QALYs gained at 12 months	0.6627 vs. 0.6197. Difference (0.0053) was not statistically significant (bootstrapped 95% CI, −0.059 to 0.103)	ICER (cost per QALY gained)	£303,774 (health and social care perspective); £541,698 (societal perspective).	Probability treatment is cost effective at WTP threshold(s)	9% probability of being cost effective	
Thomas et al. 2013 [55*, 56]	CEA/CUA (EQ-5D basecase and SF-6D sensitivity analysis)	Cost per iteration of FACETS	£3625.00	Mean difference in Global Fatigue score	−0.36 (95% CI:−0.63 to −0.08)	ICER (cost per 1-point improvement in fatigue score using the Global Fatigue Score - GFS)	£1259			The cost-effectiveness case is equivocal
		Estimated cost per person for FACETS (assuming group size of 8)	£453	QALYs gained	No significant differences between groups	ICER (cost per additional person with a clinically significant improvement in fatigue measured on global fatigue score -GFS)	£2157			
						ICER (cost per QALY gained)	Intervention is dominated (no significant QALY gain).			

Humphreys et al. [52*] evaluated the cost effectiveness of a 6 session psychological adjustment group with usual care for people with multiple sclerosis and low mood. It was a moderately sized study of 151 patients of acceptable quality. Eight months follow-up indicated significant differences in costs between the intervention and control group and the incremental cost-effectiveness ratio (using Beck Depression inventory) indicated costs pre point reduction of £118, therefore the adjustment group programme was cost effective when compared with usual care, for people with multiple sclerosis and low mood.

Thomas et al. [55*, 56] evaluated the cost-effectiveness of a six-session group-based programme for managing MS-fatigue in a multi-centre trial of 146 patients comparing cognitive behavioural and energy management techniques (FACET) with local practice. Outcomes on self-efficacy, disease specific quality of life and fatigue severity and QALYs were calculated at 1 month and 4 month follow up. The FACETs intervention had significant differences in reducing fatigue severity and increasing self-efficacy but no significant differences in MS quality of life scales or QALYs. There was an incremental cost-effectiveness ratio of 2157 per additional person with a clinically significant improvement in fatigue. The authors conclude that it was difficult to assess the additional cost in terms of cost-effectiveness as improvements in fatigue are not reflected in the QALY outcomes, with no significant differences between FACETS and CLP.

Bogosian et al. [36*] had a primary focus on reduction in distress following mindfulness-based CBT delivered via Skype sessions. This study used economic evaluations from 2012. It is a small study with less than 20 per group but the methodology was well designed and described. The mindfulness intervention was dominant (lower costs and better General Health Questionnaire score). The group had more than a 90% chance of being the most cost-effective option (compared to waiting list control) at a willingness to pay threshold of £20,000.

Mosweu et al. [79*] conducted a UK multi-centre trial with 94 patients comparing eight sessions of nurse-led CBT or supportive listening (SL). The RCT was rated as high quality, with the economic analysis rated acceptable quality. The cost effectiveness analysis was comprehensive. The authors calculated costs from the health, social and indirect care perspectives, and these were combined with additional quality-adjusted life years (QALY) or improvement on a disease specific measure (GHQ-12). Cost-effectiveness was explored at 12 months and the conclusion was that ‘nurse delivered CBT is more effective in reducing distress among MS patients compared to SL, but is highly unlikely to be cost-effective’ using QALYs or the GHQ-12.

### Summary

The papers included indicate that psychological interventions for Multiple Sclerosis have some potential to be cost-effective, with suggestion of increased cost-effectiveness for those that are more distressed or depressed.

## Cardiac studies

No systematic reviews of the cost effectiveness of psychological interventions in cardiac populations were identified.

### Overview of cardiac studies in current review

In a study conducted by Mejia et al. [75*], heart failure nurses provided patients with six, 1 hour sessions, using the “Heart Failure Plan” (the SEMAPHFOR Trial, Cockayne et al., 2014) [76]. Patients were given the programme manual, and goals were set around exercise or relaxation, and cardiac misconceptions were corrected, alongside discussion about medication and medical care. The control group consisted of patients receiving the manual alone, and a matched amount of care from heart failure nurses (see Tables 11 and 12). The authors reported that a cognitive behavioural self-management program provided little evidence of any effects on improved mental health outcomes or any evidence of cost-effectiveness on the cost of care, when compared to usual care. Future studies might compare outcomes when nurses had received more extensive training, coaching and supervision to deliver the intervention.

**Table 11:** Description of Studies: CHD

Authors, Year	Condition	CEA	CUA	Setting	N (participants)	Baseline Characteristics	Intervention/ comparator(s)	Effectiveness measure(s); cost measures (price year)	Perspective	Time horizon
Ladapo et al. 2012 [63*]	Cardiovascular		✓	USA;	N Participants:	237	Intervention: Problem Solving Therapy and/or anti-depressants versus usual care. Intervention: 80 TAU = 77 Nondepressed: 80	QALYs (SF-12/ SF 6D);	Healthcare	6mths
				Hospital inpatient and outpatients in medical and mental health	Age in years (mean):	Intervention: 59 (10.6); TAU: 61 (10.6); Nondepressed: 63 (10.3)		USD (price year not stated)		
					Sex (% female):	53				
					Ethnicity (% white):	49% Hispanic, and 19% Afro-American; no further details				
Mejia et al. 2014 [75*]	Cardiovascular		✓	UK (England);	N Participants:	260	Treatment: 6 nurse led cognitive behaviourally informed self-management sessions consisting of a manual and further facilitated input. Control group: Usual care, the same manual, but with no further nurse led manual facilitation. Self-management: 95 Usual care: 165	QALYs (EQ-5D-3L);	Healthcare	12mths
				Hospital setting	Age in years (mean):	Intervention: 70 (12.5); Control: 71 (10.9)		GBP 2008/2009 (basecase). 2011/2012 (sensitivity analysis).		
					Sex (% female):	28				
					Ethnicity (% white):	Not given				
Tyrer et al. 2017 [95*]	Non Cardiac Chest Pain		✓	UK (England);	N Participants:	68	CBT for Chest Pain based on modification of CBT for Health Anxiety versus standard care. Patients received between 4 and 10 sessions, with capacity to extend to 15 sessions if required. CBT: 34 Standard care: 34	QALYs (EQ-5D);	Health and social care	1 year
				Multi-centre	Age in years (mean, SD):	CBT: 48.91 (14.5); Standard care: 48.71 (13.5)		GBP (price year not stated)		
					Sex (% female):	CBT: 32 Standard care: 29				
					Ethnicity (% white):	CBT: 71 Standard care: 85

**Table 12:** Cost-effectiveness Outcomes: CHD

Author, Year	Type of Analysis/	Type of Costs	Costs	Type of effectiveness outcome	Effectiveness	ICER (definition);	ICER (results)	Sensitivity Analysis (definitions)	Sensitivity analysis (results)	Authors conclude (Reviewer comments where these differ from authors)
Ladapo et al. 2012 [63*]	CUA (SF-12/ SF-6D)	Costs	Intervention: $1857; Control: $2797; Adjusted mean difference− 1229 (95%CI:− 2652 to 195), p = 0.09	Utility	Intervention: 0.60; Control: 0.56; Not statistically significant; p = 0.07	ICER (cost per QALY gained):	Intervention dominates	WTP Threshold	$30,000	Reduces costs but further research needed (unclear what proportion of iterations were less costly but less effective)
								Bootstrapping (Y/N; replications):	Y; 1000	
								Probability treatment is cost effective at WTP threshold(s)	98%.	
Mejia et al. 2014 [75*]	CUA	Difference in costs (complete case analysis)	£320.99 (95% CI: -£1524 to £2166)	Difference in effectiveness (adjusting for baseline utility):	−0.02 (95% CI 0.09 to 0.05). There were no substantial differences in the utility scores between treatment groups in all follow-up assessments	ICER (cost per QALY gained)	Control dominates as results indicate reduction on utility/quality of life.	Parameters varied in sensitivity analysis	Varying price year to 2011/12, complete case analysis, multiple imputation.	The uncertainty around both estimates of cost and effectiveness mean that it is not reasonable to make recommendations based on cost-effectiveness alone.
				Difference in QALYs	−0.004			WTP Threshold	£20,000 and 30,000 per QALY	
								Probability treatment is cost effective at WTP threshold(s)	The probability that the intervention is cost-effective for thresholds between 20,000 and 30,000 is around 45%.	
Tyrer et al. 2017 [95*]	CUA (EQ-5D)	Total costs per patient	Intervention (CBT-CP): £2235.53 Standard care: £3732.02 in standard care group Difference: -£1496.49 (not significant).	Health anxiety Inventory, HADS, LMHAQ-CP scores, A and E attendances after 6 months/1 year, SEPS scores	Difference: “greater improvement in the CBT-CP group than for standard care in at 12 months compared with 6 months” (not significant). Difference in scores at 6 and 12 months between the TAU (control) and treatment (intervention) groups was not significant.	ICER (cost per QALY gained):	Not reported. CBT-CP dominated standard care (better outcomes and lower costs).	Bootstrapping (Y/N; replications):	Bootstrapping for 95% CIs for costs but no further sensitivity analysis details reported	Potentially cost-effective (needs further research).
		Hospital service costs	Difference -£177.52, “more than covered the costs of the CBT-CP”	QALY gains (from EQ-5D) reported over the follow up	Intervention: 0.76 QALYs; Control: 0.74; Difference was not significant QALY over follow up for the CBT-CP group.					
		Community costs per participant	Intervention: £480; Control: £480							

In an American study, Ladapo et al. [63*] assessed whether treatment using problem-solving therapy, anti-depressants, or both, was more cost-effective, overall, than care as usual for patients experiencing depression following a diagnosis of acute coronary syndrome (RCT data from the COPES Trial [64]). They found that the additional costs of delivering mental health treatment and anti-depressant usage was offset by the reduction in hospitalisation costs associated with cardiac events, when compared to usual care.

Collectively, the two studies demonstrate that there appears to be cost-effectiveness evidence for problem-solving therapy and/or antidepressants in cardiac settings, and more evidence is required to determine the cost effectiveness of cognitive behaviourally informed interventions.

#### Non cardiac chest pain

In a study involving patients repeatedly attending medical services with non cardiac chest pain, Tyrer et al. [95*] allocated 68 patients to receive either usual medical care, or around 4-10 sessions of cognitive behavioural therapy. No significant treatment differences were observed for health anxiety scores, social functioning, mood or quality of life, although the treatment arm showed non-significant improvements on most outcomes at 12 months, compared with 6 months. Although patients in the treatment arm had 2-3 times less hospital admissions, bed days and A and E visits, the cost differential between the groups was not significant.

### Summary

In summary, the 2 cardiac studies demonstrate that there appears to be cost-effectiveness evidence for problem-solving therapy and/or antidepressants in cardiac settings, and more evidence is required to determine the cost effectiveness of cognitive behaviourally informed interventions. With regards to non cardiac chest pain, the Tyrer et al. study [94*] demonstrated no evidence of significant cost-effectiveness of a cognitive behavioural therapy treatment, however there were low participant numbers in both the treatment and the control conditions.

## Weight management

No systematic reviews of the cost effectiveness of psychological interventions in weight management were identified and two RCTs were found.

### Weight management study in current review

In the RCT conducted by Hersey et al. [51*], a cognitive behavioural weight loss intervention was used to improve diet and exercise (see Tables 13 and 14). The intervention consisted of an interactive web site, in addition to either brief web based telephone or internet based counselling, in three arms (1: basic web based approach and written information; 2: interactive web based approach and written information; 3: written information, interactive web based approach, and telephone/email coaching support). Outcomes were not significantly different across treatment arms. The intervention included goal setting, problem solving, self-monitoring and the development of social support for lifestyle change. Participants were also given a manual and asked to submit weekly self-monitoring records of weight, food intake, and physical activity. Weight loss was significant for all 3 groups (− 3.5, − 3.8% and − 5.1% of overall body weight for each arm respectively at 15-18 months), blood pressure was lowered and physical activity improved. The authors report that the cost of each intervention arm was cost effective when compared with projected medical costs. Retention rates were much lower than expected for this study, however the authors argued this did not affect the internal validity.

**Table 13:** Description of Studies: Weight Management

Authors, Year	Condition	CEA	CUA	Setting	N (participants)	Baseline Characteristics	Intervention/ comparator(s)	Effectiveness measure(s); cost measures (price year)	Perspective	Time horizon
Hersey et al. 2012 [51*]	Obesity/ overweight		✓	USA:	N Participants:	1755	Lifestyle coaches delivered: Group 1 (598): Manual and Internet based intervention using supervised motivation interviewing approach. Group 2 (579): Manual and Internet, with tailored computerised feedback on weekly reports submitted. 3. As per Group 2(578), but users offered alternate weekly telephone calls/personalised emails.	QALYs (derived from literature-based utility weights);	Healthcare	18mths
				4 Midwestern US states	Age in years (mean, range):	46.7 years (18-64)		USD (2007)		
					Sex (% female):	74				
					Ethnicity (% white):	84				
Perri 2014 [86*]	Obesity/ overweight	✓		USA;	N Participants:	612	The 3 group interventions were mostly group based with some telephone consultations, and behaviour modification strategies included “goal setting, self-monitoring, stimulus control, cognitive restructuring, and problem solving”. 1. 16 sessions. 2. 32 sessions. 3. 48 sessions. The control condition comprised of 16 sessions of weight loss information and group discussions. Low: 148 Medium: 134 High: 161 Control: 169	Kilogram lost per participant	Healthcare perspective	24mths
				Multi-centre	Age in years (mean, SD):	Control: 52.0 (10.8) Low: 51.5 (12.3) Medium: 52.8 (10.6) High: 53.2 (12.0)		USD (2007)		
					Sex (% female):	78.30%				
					Ethnicity (% white):	77.7

**Table 14:** Cost-effectiveness Outcomes: Weight Management

Authors, Year	Type of Analysis/	Type of Costs	Costs	Type of effectiveness outcome	Effectiveness	ICER (definition);	ICER (results)	Sensitivity Analysis (definitions)	Sensitivity analysis (results)	Authors conclude (Reviewer comments where these differ from authors)
Hersey et al. 2012 [51*]	CUA	Mean cost per participant	Group 1: $145; Group 2: $160. Group 3: $390	Mean weight loss (%)	Group 1: 4.1%, Group 2: 3.9%, Group 3: 5.3%.	ICER (cost per 1% weight loss) vs “do nothing” approach; Group 3 vs Group 1 & 2.	Group1: $30, Group 2: $40, Group 3: £70; $200.			Cost-effective “Extrapolation of savings for the entire TRICARE population would significantly reduce direct medical costs”
				Mean life year gain	Group 1: 0.17, Group 2: 0.16, Group 3: 0.21.	ICER (cost per life year gained) vs “do nothing” approach; Group 3 vs Group 1 & 2.	Group 1: $900, Group 2: $1000, Group 3: $1800; $4200–$5300.			
						ICER (cost per QALY gained) vs “do nothing approach”; Group 3 vs Group 1 & 2.	Group 1: $900, Group 2: $1100, Group 3: $1900; $4400–$5600.			
				Mean QALY gain	Group 1: 0.16; Group 2: 0.15; Group 3: 0.20.					
Perri 2014 [86*]	CEA (kg decrease in weight)	Total costs	Intervention: (Low dose): $16,351, (Moderate) $19,426, (High): $26,630; Control: $13,233;	Mean % initial body weight lost (kg) at 6 months	Intervention: Low dose: 7.2% (95%CI 6.1, 8.3), Moderate: 9.3% (95%CI 8.2, 10.3) High dose: 10.9% (9.8,11.9); Control: 4.1% (95% CI:3.1, 5.1)	ICER Cost per kg lost per participant	Low $33; Moderate: $22; High: $25; Control:$28			Low-dose treatment is less effective and less cost-efficient than moderate-dose. A moderate dose can produce clinically meaningful, two-year reductions in body weight comparable to high-dose treatment, at a lower cost. (Unclear. ICER estimate for 2 years is lower for high dose than moderate. At six months there is a dose-response relationship between the interventions compared to control).
		Cost per participant	Intervention (Low dose): $111, (Moderate): $145, (High): $165 Control: $78.	Mean % initial body weight lost (kg) at 24 months:	Intervention: Low dose: 3.5% (95%CI 2.0, 4.8), Moderate: 6.7% (95%CI 5.3, 7.9) High: 6.8% (5.5,8.1); Control: 2.9% (95% CI: 1.7, 4.3) at 24 months					

In the Perri et al. study [86*], 612 adults living in rural communities in the U.S. were assigned to low, moderate or high doses of a behavioural weight loss treatment (16, 32, or 48 sessions over 2 years), or to a control condition with nutritional information only. Mean body weight reductions at 2 years were as follows: Control Group: 2.9%, Low dose: 3.5%, Moderate Dose: 6.7%, and High Dose: 6.8%. The moderate dose treatment delivered comparable outcomes to the high dose treatment, but at a lower cost, and therefore the moderate does treatment was considered to be the most cost effective condition. As the study only included people living in rural settings with a BMI of 30-45, future studies will hopefully investigate whether these results are replicable in urban settings, and for those with a BMI over 45.

### Summary

In summary, the Hersey et al. [51*] study demonstrated that a cognitive behaviourally informed weight loss program was cost effective when compared to projected medical costs, and the Perri et al. study [86*] found that a moderate dose of a behavioural weight loss treatment was the most cost-effective.

## Other conditions

### Medically unexplained symptoms

No systematic reviews of the cost effectiveness of psychological interventions in populations with medically unexplained symptoms were identified.

#### Overview of medically unexplained symptoms studies in current review

Four primary studies [43, 91, 101, 103] examined persistent physical symptoms which were causing distress and for which no medical diagnosis had been found (see Tables 15 and 16). A range of terms were used by these studies – “functional somatic syndromes”, “multi-somatoform disorder”, “unexplained physical symptoms” and “somatic disorder”.

**Table 15:** Description of Studies: Medically Unexplained Symptoms

Authors, Year	Condition	CEA	CUA	Setting	N (participants)	Baseline Characteristics	Intervention/ comparator(s)	Effectiveness measure(s); cost measures (price year)	Perspective	Time horizon
Chernyak et al. 2014 [43*]	Medically unexplained symptoms		✓	Germany	Participants:	211	12 weekly sessions of psychodynamic interpersonal therapy (PIT), delivered by clinicians trained in psychotherapy, compared to enhanced medical care. Intervention: 107 Control: 104	SF-36;	Healthcare	1 yr
				Patients from 6 academic outpatient centres	Age in years (mean):	Intervention: 47.9; Control: 48.0		Euros (2012)		
					Sex (% female):	Intervention: 67; Control: 72				
					Ethnicity (% white):	Not given				
Schroder et al. 2017 [91*]	Medically unexplained symptoms		✓	Denmark;	N Participants:	120	9 modules of manualised group CBT, delivered by psychiatrists, versus enhanced usual care. Intervention: 54 Control: 66	SF-36 converted to SF-6D utility scores;	Healthcare and societal	40mths
				Hospital	Age in years (mean, SD):	Intervention: 35.4 (6.3) Control: 36.2 (6.5)		Euros (2010 prices)		
					Sex (% female):	Intervention: 74; Control: 83				
					Ethnicity (% white):	Not given				
van Ravesteijn et al. 2013 [101*]	Medically unexplained symptoms		✓	Netherlands;	N Participants:	96	8 × 2.5 hr. group sessions of MBCT delivered by experienced mindfulness trainers, compared with enhanced usual care. Intervention: 55 Control: 41	SF-36;	Healthcare and societal	12mths
				Frequently attending patients in primary care	Age in years (mean, SD):	Intervention: 47.0 (11.3), Control: 48.1 (12.3)		Euros (2010)		
					Sex (% female):	Intervention: 80.3; Control: 67.9				
					Ethnicity (% white):	Not given				
Visser et al. 2015 [103*]	Medically unexplained symptoms		✓	Netherlands;	N Participants:	162	cognitive-behavioural group training (2 hr. weekly sessions over 3mths) compared to a wait-list control group Intervention: 84 Control: 78	SF-36;	Healthcare and societal	3 mths (12 month uncontrolled follow up; costs, utilities & QALYs modelled over 4 years)
				Outpatient clinics at general hospitals and a secondary community mental health service	Age in years (mean, SD):	Intervention: 46, Control: 42		Euros (2011)		
					Sex (% female):	Intervention: 80; Control: 82				
					Ethnicity (% white):	Not given

**Table 16:** Cost-effectiveness Outcomes: MUS

Authors, Year	Type of Analysis/	Type of Costs	Costs	Type of effectiveness outcome	Effectiveness	ICER (definition);	ICER (results)	Sensitivity Analysis (definitions)	Sensitivity analysis (results)	Authors conclude (Reviewer comments where these differ from authors)
Chernyak et al. 2014 [43*]	CUA	Costs (direct treatment):	Intervention: €893; Control: €141	SF-36 PCS (mean improvement):	Intervention: 5.3; Control: 2.2	ICER (cost per QALY gained):	€41,840 per QALY gained	Parameters varied	Handling of missing data (exclude, LOCF and imputation)	Highly uncertain
				SF-6D (improvement)	Intervention: 0.09; Control: 0.04			Bootstrapping Y/N (iterations):	Y (5000 replications);	
								WTP Threshold(s)	€35,000 per QALY gained	
								Probability cost-effective at WTP	“exceeded 50%”	
Schroder et al. 2017 [91*]	CUA	Annual healthcare costs	STreSS at 1 year pre treatment €3544, 4 months €2369, 1 year €2250, 2 years €2560, 3 years €523; EUC at 1 year pre-treatment,€4106 at 4 months €976, at 1 year €4200, at 2 years €3937 and 3 years €1132	% achieving clinically significant improvement during 16 months, defined as 0.5 SD change (4 point increase) on SF-36 aggregate score and 0.35 points reduction on the SCL-90 R somatisation subscale.	STreSS: 45% (30–60); EUC: 17% (7–27)	ICER (cost per additional patient with clinically significant improvement, cost per QALY gained using healthcare costs only, cost per QALY gained using total costs):	€3035 to €4398 per patient improved; Intervention was dominant per QALY gained when healthcare costs used; €24,640 euros per QALY gained at 16 months when total costs used.	Bootstrapping Y/N (iterations):	Y (1000 replications)	Cost effective per QALY gained (for societal perspective the cost-effectiveness may be underestimated)
		Annual costs (direct plus indirect including tax income)	STreSS at 1 year pre-treatment €12,489, 4 months €5487, 1 year €11,118, 2 years €9353, 3 years €6334; EUC at 1 year pre-treatment €15,904, at 4 months €3888, at 1 year €14,799, at 2 years €16,109 at 3 years €15,701	QALYs accrued at 16 months (SF-36 converted to SF-6D)	STreSS: 0.80; EUC: 0.75			WTP Threshold(s)	€5000 per additional patient achieving clinically significant improvement, €25,000 to 35,000 per QALY	
								Probability cost-effective at WTP	93–95% (health-care perspective); 50-55% (societal perspective but healthcare threshold used)	
van Ravesteijn et al. 2013 [101*]	CUA	Mean societal costs over 1 year (bootstrapped):	Intervention: €6269; Control: €5617; Total costs were not significantly different, but mental health care costs were higher and hospital care costs lower in the intervention group.	Change in utility score over 1 year	Intervention: 0.06; Control: 0.04	ICER (cost per QALY gained):	Societal perspective: €56,637 per QALY gained Healthcare perspective: €66,450	Parameters varied	Scenario analysis that varied perspective, and using per protocol trial data (ICERs ranged from €41,167 to €53,198)	Uncertain whether MBCT is cost-effective.
		Mean costs of intervention	Intervention: €450; Control: N/A	QALYs gained	Intervention: 0.674; Control: 0.663; Bootstrapped difference in QALYs 0.012 (95% CI −0.019 to 0.041) was not statistically significant.			Bootstrapping Y/N (iterations):	Y, (1000 replications).	
								WTP Threshold(s)	€0 to €80,000	
								Probability cost-effective at WTP	Societal perspective: At WTP threshold of €0, the probability of MBCT being cost effective is 28%. At WTP of €40,000 this is 48%. At €80,000 it is 57%. Healthcare perspective results “did not significantly differ [from societal perspective]”, At a WTP threshold of €80,000 the probability that MBCT is cost-effective is 55%.	
Visser et al. 2015 [103*]	CUA (Markov model)	Societal costs over 4 years	Intervention: €32,929; Control: €33,757	QALYs gained	Intervention: 2.35 QALYs; Control: 2.29 QALYs	ICER (cost per QALY gained):	Intervention dominant from societal perspective; €8165 for healthcare perspective	Parameters varied	Probabilistic sensitivity analysis (10,000 iterations). Scenario analysis including only healthcare perspective, time horizon.	Cost effective
		Healthcare costs over 4 years	Intervention: €21,757; Control: €21,278					WTP Threshold(s)	€30,000 per gained QALY	
								Probability cost-effective at WTP	80% at WTP threshold.	

Two studies were conducted in The Netherlands [101, 103], one in Denmark [91*] and one in Germany [43*]. No UK studies were found.

The psychological approaches that were used were cognitive-behavioural group training [103*], group cognitive behavioural therapy (CBT) [91*], brief psychodynamic interpersonal therapy [43*], and mindfulness-based cognitive therapy [101*]. All four studies considered both healthcare and societal costs.

Visser et al. [103*] examined the cost-effectiveness of cognitive-behavioural group training (2-hr weekly sessions over 3 months) compared to a wait-list control group, for patients with unexplained physical symptoms. The authors claimed that theirs was the first study to use a state-of-the-art health economic model and Quality Adjusted Life Years (QALYs) to assess the cost-effectiveness of treatments for unexplained physical symptoms. Using a probabilistic Markov model with data from a randomised controlled trial, they estimated that cognitive-behavioural group training was dominant at 4 years. Based on the model, an Incremental Cost Effectiveness Ratio (ICER) of 30,000 euros per QALY was reached after 18 months and the group training was cost saving after 33 months.

Schroder et al. [91*] conducted an economic evaluation of 9 modules of manualised group CBT, delivered by psychiatrists, versus enhanced usual care for functional somatic syndromes. They found that in the medium term (16 months), the probability that the intervention was cost-effective at 25,000 to 35,000 euros per QALY was 93–95% from a healthcare perspective, and 50–55% from a societal perspective. They concluded that the cost of the intervention (average 1545 euros per patient) was more than offset by subsequent savings in direct and indirect costs.

Chernyak et al. [43*] compared 12 weekly sessions of psychodynamic interpersonal therapy (PIT), delivered by clinicians trained in psychotherapy, with enhanced medical care (EMC) for patients with multi-somatoform disorder. EMC was manual-based and consisted of three half-hour sessions at 6-week intervals with specifically trained physicians. The probability of PIT being cost-effective exceeded 50% for willingness to pay levels higher than 35,000 euros per QALY, with a mean ICER of 41,840 euros per QALY gained. The authors concluded that that cost-effectiveness of PIT is highly uncertain for thresholds of willingness to pay under 35 thousand Euros per QALY.

Van Ravesteijn et al. [101*] examined the cost-effectiveness of eight 2.5-hour group sessions of mindfulness-based cognitive therapy (MBCT), delivered by experienced mindfulness trainers, compared with enhanced usual care (EUC) for patients with persistent medically unexplained symptoms. MBCT was more effective but costlier than EUC, resulting in an ICER of 56,637 euros per QALY gained. At a willingness to pay of 80,000 euros per QALY, the probability that MBCT would be cost-effective was 57%. The authors conclude that MBCT had a clinically relevant effect on health-related quality of life, but that it “remains uncertain” whether MBCT is cost-effective.

Based on the four studies of medically unexplained symptoms in this review, group CBT and cognitive-behavioural group training were found to be cost-effective, while the cost-effectiveness of brief psychodynamic interpersonal therapy and MBCT was uncertain with ICERs of 41,840 Euros and 56,637 euros per QALY respectively.

## Insomnia

### Previous systematic reviews of economic evaluations of psychological interventions in insomnia

A review of the literature to 2015 on the health economics of pharmacological and non-pharmacological treatments for insomnia, by Wickwire et al. [27], included three studies that investigated psychological treatments and used RCT data. These included one pre-2012 study which fell outside the date range of the current review. In that study patients with insomnia, who were on long-term hypnotic drugs, were randomised to a 6-session CBT for Insomnia (CBT-I) or wait-list control [116]. Based on health care costs at 6 months, the mean incremental cost per QALY was $7313 (£3418 in 2003 GBP). If future costs were assumed to remain static the intervention was found to become cost effective in year four, and if future costs were assumed to decline linearly, the ICER decreased to approximately $578 (£270 in 2003 GBP) per QALY in year 10 [116]. Overall, Wickwire et al. concluded that that both pharmacological, as well as behavioural therapy, were cost-effective for insomnia [27].

### Insomnia study in current review

The current review identified one subsequent study, published after Wickwire et al’s review, giving a total of three studies on the cost-effectiveness of psychological interventions for insomnia published since 2012 (see Tables 17 and 18) [37, 93, 107]. The three studies were from Japan [107*], England [37*] and Germany [93*], and all examined CBT for Insomnia (CBT-I) delivered individually [107*], through a group workshop [37*], or via the internet [93*].

**Table 17:** Description of Studies: Insomnia

Authors, Year	Condition	CEA	CUA	Setting	N (participants)	Baseline Characteristics	Intervention/ comparator(s)	effectiveness measure(s); cost measures (price year)	Perspective	Time horizon
Bonin et al. 2014 [37*]	Insomnia	✓	✓	England	Participants:	151	one-day CBT-I group workshop, led by two psychologists with CBT expertise, compared to a wait list control group. Intervention: 75 Control: 76	EQ-5D; ISI;	Healthcare	3mths
				Participants from 5 London boroughs who self-referred	Age in years (mean, SD):	Not given		£ (2008-09)		
					Sex (% female):	72				
					Ethnicity (% white):	81				
Thiart et al. 2016 [93*]	Insomnia	✓		Germany	N Participants:	128	internet-based CBT-I for school teachers with insomnia (6 modules with email feedback by trained clinical psychologists), compared to a waitlist control. Intervention: 64 Control: 64	Insomnia Severity Index (ISI), Reliable Change Index (RCI);	Societal	6mths
				Schools (recruiting teachers)	Age in years (mean, SD):	48.0 (9.9)		Euros (2013)		
					Sex (% female):	74.2				
					Ethnicity (% white):	Not given				
Watanabe et al. 2015 [107*]	Insomnia		✓	Japan	N Participants:	37	4 weekly individual sessions of CBT-I, based on a published treatment manual, compared to treatment as usual. Intervention: 20 Control: 17	‘Depression free days’ (DFD) (constructed from Ham-D Rating Scale scores), mapped to utility scores.	Healthcare	8wks
				Psychiatric outpatients	Age in years (mean, SD):	50.5 (11.1),		US$ (2013)		
					Sex (% female):	62.2				
					Ethnicity (% white):	Not given

**Table 18:** Cost-effectiveness Outcomes: Insomnia

Authors, Year	Type of Analysis/	Type of Costs	Costs	Type of effectiveness outcome	Effectiveness	ICER (definition);	ICER (results)	Sensitivity Analysis (definitions)	Sensitivity analysis (results)	Authors conclude (Reviewer comments where these differ from authors)
Bonin et al. 2014 [37*]	CEA & CUA	Costs	*Intervention: £251.00;* *Control: £72.00*	ISI scores (mean):	Intervention: Reduced by 17.6% (P < 0.001); Control: Reduced by 3.5% (NS: p0.077)	ICER (cost per 1 point improvement in ISI, per additional person in subclinical ISI, per QALY gained):	Not reported	Bootstrapping Y/N (iterations):	Y, 10,000	Authors determined the cost-effectiveness was likely but depends on society’s WTP for ISI change.
				QALYs (mean):	Intervention 0.19; Control: 0.17 (QALY gain NS)			WTP Threshold(s)	£150 per 1 point improvement in ISI, £1800 per additional person in subclinical ISI state, £30,000 per QALY gained.	For cost per QALY gained the authors considered the cost-utility to be unclear but there is an error in the abstract (Not likely to have cost utility)
								Probability cost-effective at WTP	97% for 1 unit improvement, 80% for additional person in subclinical ISI state, 34% per QALY gained (NB: misinterpreted in abstract).	
Thiart et al. 2016 [93*]	CEA	Total employment costs per person at 6 months (absenteeism, presenteeism)	Intervention: €2527.47; Control: €2945.10; difference: €417.63	Mean (SD) improvement on the ISI scale	Intervention: 9.3 (5.0); Control: 2.6 (4.4)	ICER (cost per every additional participant with a positive treatment response i.e. < 8 points on ISI and reliable change after 6 months)	-€1512 euros (95% CI: − 4493 to 1128). Intervention dominates	Parameters varied	Intervention costs (€100 and €300)	Cost effective
		Intervention costs (per person)	Intervention: €200; Control: N/A			NMB (benefits quantified in monetary terms minus costs of the intervention)	417.63 (− 593.03 to 1488.70)	Bootstrapping Y/N (iterations):	bootstrapping method with 2500 replications	
						Benefit cost ratio (€ gains for every € invested)	3.09 (−1.97 to 8.44)	WTP Threshold(s)	€0, €761, €1115 for a treatment response to per treatment response.	
		Difference in presenteeism plus absenteeism between groups (€ - not including cost of intervention)	617.43			Return on investment (ROI i.e. [(benefit-cost)/(costs × 100)], 95% CI:	2.08.81 (− 296.52 to 744.35)	Intervention dominates (81-91% of iterations in south east cost effectiveness quadrant); 60-72%	Base case: 87% at WTP of €0, 95% at WTP of €761; Sensitivity analysis (€300 intervention costs): 81% at WTP of €0, 95% at €1115.	
Watanabe et al. 2015 [107*]	CUA	Direct costs	Intervention (CBT-I plus treatment as usual): $702 (SD 175) Control (TAU alone): $448 (SD 115) Non-significantly higher costs with an incremental value of $254 (SD: 203)	QALYs (literature derived and based on depression free days receiving a utility score of 1 otherwise major depressive disorder utility score of 0.59 was used depending on how patients had scored the 17-item HAND - less than 7 was deemed as remission from depression and severe depression was if their score was 27 or more).	Intervention: 0.139 (SD 0.004) QALY. Control: 0.120 (SD 0.004) QALY QALYs were statistically significantly higher (*P* = 0.002) in the CBT-I-plus-TAU group than in the TAU-alone group.	ICER (cost per QALY gained):	Base case: US$13,678 (95% CI: − 5691 to 71,316). Range in sensitivity analysis: US$5900 (95% CI:2485 to 14,958) to US$42929 (95% CI: 16994 to 163,146).	Parameters varied	Various different “approaches” used to test the results including and excluding hospital stays for depression, weighting utilities for severity versus looking at depression-free days and varying the costs of psychotherapy	adding CBT-I is highly likely to be cost-effective for patients with residual insomnia and concomitant depression (potentially problematic conclusion given we don’t know their methods for identifying literature values for utility with and without depression that were used to derive QALYs)
								Bootstrapping Y/N (iterations):	Y (1000 iterations).	
								WTP Threshold(s)	US$0 to US$100,000. Authors cite that one QALY is often valued at 50,000–70,000 USD	
								Probability cost-effective at WTP	95% if a decision-maker was willing to pay 60,000 USD per QALY gained, and approximately 90% at 40,000 USD	

Watanabe et al. [107*] compared four weekly individual sessions of CBT-I, based on a published treatment manual, with treatment as usual (TAU) for patients with major depressive disorder and chronic insomnia. They calculated an ICER of US$13,678 per QALY gained and estimated that adding CBT-I to TAU demonstrated an approximately 90% chance of gaining one more QALY at a willingness to pay of US$40,000. The authors regarded the results as ‘promising’ but acknowledge limitations, including the study’s small sample size (*n* = 37) and short follow up (8 weeks). They recommended more trials with larger samples and longer follow up.

Bonin et al. [37*] examined a one-day CBT-I group workshop, led by two psychologists with CBT expertise, compared to a wait list control group. They calculated that at a maximum willingness to pay of £30,000 the probability that the intervention is cost-effective was only 34%, due to a small and nonsignificant QALY gain (based on EQ-5D quality of life scores) in the intervention group relative to the control group. However, the authors argued that the intervention had a very high probability of being cost-effective in terms of improvement on the Insomnia Severity Index (ISI). They calculated that, even if running at only 53% capacity, the intervention had a 95% probability of being cost-effective at a WTP of £150 (approximately the cost of the intervention) per point improvement on the ISI. The authors cautioned that their findings should be regarded as indicative rather than definitive, but suggested such workshops are a promising low-level option to help increase access to psychological therapies.

Thiart et al. [93*] conducted an economic evaluation of internet-based CBT-I for school teachers with insomnia, involving six 1-week modules with email feedback by trained clinical psychologists, compared to a waitlist control. The study involved a cost-effectiveness analysis that looked at the cost per change in the Insomnia Severity Index (ISI); and an analysis of the costs and benefits to the employer, focusing on absenteeism and presenteeism costs. The ICER was estimated at 1512 euros for every participant with a positive treatment response after 6 months. The probability of the intervention being cost-effective was 87% at a potential willingness-to-pay of zero. A return on investment of 208% was calculated, with cost savings mainly due to the effects of the intervention on presenteeism and to a lesser degree by reduced absenteeism.

Thiart and colleagues [93*] suggested that one possible reason their findings were more positive than the two other studies was that the previous studies focused on healthcare costs, whereas Thiart and colleagues [93*] focused on non-health-related indirect costs.

### Summary

Each of three included insomnia studies examined a different approach to CBT for insomnia – group, individual and internet-based. All three concluded that CBT was likely to be cost-effective, however the studies on group CBT [37*] and individual CBT [107*] were not conclusive. Only Thiart et al. [93*], in their study of internet-based CBT, were able to conclude that the intervention was dominant, possibly because their study was the only one to include societal as well as healthcare costs.

## Stroke

No systematic reviews of the cost effectiveness of psychological interventions in populations with stroke were identified.

### Overview of stroke studies in current review

Two studies were identified (see Tables 19 and 20) that had looked at behavioural therapy interventions for stroke patients; one addressing aphasia [54*] and one focusing on depressive symptoms [99*].

**Table 19:** Description of Studies: Stroke

Authors, Year	Condition	CEA	CUA	Setting	N (participants)	Baseline Characteristics	Intervention/ comparator(s)	effectiveness measure(s); cost measures (price year)	Perspective	Time horizon
Humphreys et al. 2015 [54*]	Stroke	✓		UK	N Participants:	105	up to 20 behavioural therapy sessions over 3mths, at the participant’s place of residence, by an assistant psychologist with weekly supervision, compared to usual care. Intervention: 51 Control: 54	Stroke Aphasic Depression Questionnaire Hospital version 21 (SADQH21) score;	Health and social care	24 months
				Participants recruited from hospital and community setting	Age in years (mean, SD):	Intervention: 65.5 (13.9); Control: 68.5 (13.1)		Euros 2011 (€)		
					Sex (% female):	Intervention: 31 Control: 43				
					Ethnicity (% white):	Not given				
van Eeden et al. 2015 [99*]	Stroke	✓	✓	Netherlands;	N Participants:	61	CBT, 10–12 individual sessions with a certified psychologist, augmented by goal-setting sessions of occupational therapy or movement therapy, compared to individual, patient-tailored computerised cognitive training programme (CogniPlus). Intervention: 31 Control: 30	HADS, EQ-5D-3L (Dutch tariff);	Societal	12 months
				Hospital/ rehabilitation centres (multi-centre)	Age in years (mean, SD):	Intervention: 62.2 (8.3); Control: 60.0 (10.5)		Euros (2012)		
					Sex (% female):	Intervention: 38.7; Control: 36.7				
					Ethnicity (% white):	Not given

**Table 20:** Cost-effectiveness Outcomes: Stroke

Authors, Year	Type of Analysis/	Type of Costs	Costs	Type of effectiveness outcome	Effectiveness	ICER (definition);	ICER (results)	Sensitivity Analysis (definitions)	Sensitivity analysis (results)	Authors conclude (Reviewer comments where these differ from authors)
Humphreys et al. 2015 [54*]	CEA	Incremental cost increase per patient over 24 months (extrapolated):	Intervention: £1388.90, not including cost of the intervention (£3349.90 including intervention cost); Control: £1541.70	Change in SADQH-21 scale	Intervention: −6; Control: + 0.7 (*p* = 0.003)	ICER (cost per point reduction on the SADQH21 scale)	£263	Bootstrapping Y/N (iterations):	Y (1000 replications)	Encouraging/promising (unclear - don’t know what society’s WTP is for a reduction in SADQH21)
								WTP Threshold(s)	£263 per point reduction in the SADQH21 score	
								Probability cost-effective at WTP	100%	
van Eeden et al. 2015 [99*]	CEA and CUA	Societal costs over 12 months	Intervention: €8064; Control: €9998; Different between the control group and the augmented CBT group not significant (95% CI:− 5284, 1796).	Change in HADS score	−0.8;	Cost per one point improvement in the HADS	ICER: €2395.3 (extendedly dominated)	Parameters varied	Price for a rehabilitation day treatment to a regular rehabilitation, varied consultation price, the friction cost method to calculate productivity costs instead of human capital approach, used a healthcare perspective and different sets of tariffs for utilities (Dutch and UK). (notably that for healthcare perspective the ICER is €107.454.70 and the intervention is no longer dominant)	Not cost-effective on the HADS; Unclear cost effectiveness per QALY gained - no significant effect on costs or QALYs
		Intervention costs	Intervention: €1130; Control: €592 (CogniPlus control cost)	QALYs gained	Intervention group gained slightly more QALYs - mean 0.01	Cost per QALY gained	ICER - intervention dominant (although due to minimal difference in effects of 0.01 QALY gain) “these results should be interpreted with caution”.	Bootstrapping Y/N (iterations):	Y, (5000 replications).	
								WTP Threshold(s)	€0 to €40,000	
								Probability cost-effective at WTP	At WTP threshold of €2500, the probability of the augmented CBT intervention being cost-effective was 49%; At WTP of €40,000, the augmented CBT intervention had a 76% probability of being cost-effective.	

In a UK study, Humphreys et al. [54*] evaluated the cost effectiveness of a behavioural therapy intervention for stroke patients with aphasia, compared to usual care. The intervention provided up to 20 behavioural therapy sessions over 3 months, delivered at the participant’s place of residence by an assistant psychologist who received weekly supervision from a consultant psychologist. The study did not include a formal measure of health-related quality of life so did not calculate QALYs. It did find a significant impact of the intervention on mood, as measured by the Stroke Aphasic Depression Questionnaire Hospital version 21 (SADQH21) scale. The cost analysis, undertaken from the perspective of health and social services, found that every point reduction on the SADQH21 scale cost £263. The authors suggested that the results were promising and recommended further investigation of the approach.

Van Eeden et al. [99*] conducted an economic evaluation of an augmented CBT intervention compared to computerised cognitive training for patients in the Netherlands with post-stroke depressive symptoms. The intervention consisted of 10–12 individualised CBT sessions with a certified healthcare psychologist, augmented by three or four goal-setting sessions of occupational therapy or movement therapy. The control group received an individual, patient-tailored computerised cognitive training programme (CogniPlus), involving 13–16 sessions over 4 months under the supervision of a research assistant or psychological assistant. From a societal perspective, the intervention was less costly and slightly more effective than the control in terms of quality of life (QALYs/EQ-5D-3 L), but less effective on the Hospital Anxiety and Depression Scale (HADS). The authors concluded that the results on the cost-effectiveness of the intervention were not convincing. Based on a willingness to pay of 40,000 euros per QALY, the augmented CBT intervention had a 76% chance of being cost-effective.

## Asthma

No systematic reviews of the cost effectiveness of psychological interventions in Asthma populations were identified.

### Asthma study in current review

Only one RCT investigating the cost effectiveness of psychological therapy in asthma was identified (see Tables 21 and 22). A UK study by Parry et al. [85*] investigated individual CBT compared to usual care for adults with anxiety complications of asthma. The intervention involved a one and a half hour introductory session followed by four to six weekly, or fortnightly, 1 hour sessions, with two follow up sessions, if these were judged to be necessary, also carried out. Although there was a significantly greater reduction in asthma-specific fear for people in the CBT group, the clinical significance of the reduction was modest. The study found a small but significant reduction in EQ5D scores for the treatment group at 6 month follow up, which the authors were unable to explain, but which they speculated may have been a psychological effect of loss of support, due to the end of participation in the trial.

**Table 21:** Description of Studies: mixed conditions

Authors, Year	Condition	CEA	CUA	Setting	N (participants)	Baseline Characteristics	Intervention/ comparator(s)	effectiveness measure(s); cost measures (price year)	Perspective	Time horizon
Larsen et al. 2016 [65*]	Dermatology		✓	Norway;	N Participants:	169	telephone-based individualised motivational interviewing, as a follow-up to a 3-week climate therapy/heliotherapy (CHT) programme, compared to TAU following the CHT. Intervention: 86 Control: 83	15D instrument of health-related quality of life;	Healthcare and employment	6mths
					Age in years (mean, SD):	Intervention: 46.2 (12.7); Control: 46.5 (13.0)		Euros (2012)		
					Sex (% female):	Intervention: 40.7 Control: 47				
					Ethnicity (% white):	Not given				
Maes et al. 2014 [73*]	Tinnitus		✓	Netherlands;	N Participants:	492	stepped care, including an individual consultation with a psychologist then a programme comprising key elements of CBT for those with moderate or severe tinnitus compared to standard stepped care treatment. Intervention: 245 Control: 247	Health Utilities Index Mark III (HUI)	Healthcare and societal	12mths
				Patients referred to an audiology centre	Age in years (mean, SD):	54.21 (11.52)		Converted to US$ (from Euros) (2009)		
					Sex (% female):	37.2				
					Ethnicity (% white):	Not given				
Parry et al. 2012 [85*]	Asthma	✓	✓	England;	N Participants:	94	Individual CBT (introductory session followed by 4-6 weekly or fortnightly 1 hr. sessions, with two follow up sessions if considered necessary, compared to usual care. Intervention: 50 Control: 44	Panic-fear sub-scale of the Asthma Symptom Checklist, EQ-5D-3L;	Healthcare	6 mths
				Sheffield	Age in years (mean, range):	Intervention: 47.0 (28-65) Control: 43.8 (25-61)		£ (year not given)		
					Sex (% female):	Intervention: 60.7 Control group: 64.5				
					Ethnicity (% white):	Not given				
Rolving et al. 2016 [89*]	Surgery		✓	Denmark;	N Participants:	90	group-based CBT, six 3-hour sessions, four prior and two post-surgery (at 3 and 6mths), delivered by a multidisciplinary team, compared to usual care. Intervention: 59 Control: 31	EQ-5D scores (Danish tariff);	Societal	12mths
				Hospital	Age in years (mean, range):	mean age 50.1 (28-64),		Euros (2014)		
					Sex (% female):	57				
					Ethnicity (% white):	Not given				
Tyrer et al. 2014 [94*]	Secondary care	✓	✓	England;	N Participants: Control:	444	CBT for health anxiety (CBT-HA), 5-10 sessions with additional booster sessions allowed, compared to standard care. Intervention: 219 Control: 225	HAI, EQ-5D;	Health and social care	24 months
				Hospitals (multi-centre)	Age in years (mean, SD):	Intervention: 50·3 (13·6); Control: 47·0 (13·4)		£ (2008-09 financial year)		
					Sex (% female):	Intervention: 52%; Control: 55				
					Ethnicity (% white):	Intervention: 80; Control: 76				
van der Aa et al. 2017 [96*]	Vision impairment	✓	✓	Netherlands and Belgium;	N Participants:	265	Stepped care (step 1 watchful waiting, 2 guided self-help course based on CBT, 3 problem solving treatment, 4 referral to GP) compared to usual care. Intervention: 131 Control: 134	HADS-A, CES-D, EQ-5D-3L (Dutch tariff);	Healthcare and societal	24mths
				Outpatient clinics	Age in years (mean, SD):	Intervention: 72.4 (12.5); Control: 74.9 (11.9)		Euros (2013)		
					Sex (% female):	Intervention: 69%; Control: 70				
					Ethnicity (% white):	Not given

**Table 22:** Cost-effectiveness Outcomes: Mixed Studies

Authors, Year	Type of Analysis/	Type of Costs	Costs	Type of effectiveness outcome	Effectiveness	ICER (definition);	ICER (results)	Sensitivity Analysis (definitions)	Sensitivity analysis (results)	Authors conclude (Reviewer comments where these differ from authors)
Larsen et al. 2016 [65*]	CUA	Mean (SD) total costs (including healthcare, intervention and work absenteeism):	Intervention: €4212 (5931); Control: €5992 (7948)	General QoL	no persistent impact on general QOL	ICER (cost per QALY gained):	Intervention is dominant using DLQI	Bootstrapping Y/N (iterations):	Bootstrapping (1000 replications)	Cost effective
		Mean (SD) healthcare costs (excluding work absenteeism):	Intervention: €1606 (SD 1281); Control: €2708 (3928);	15D (utilities)	no significant impact of intervention regarding QALY			WTP Threshold(s)	€62,500 for a health gain	
		Mean cost per participant for the intervention	€243			ICER (cost per QALY gained):	Intervention is extendedly dominated using 15D	Probability cost-effective at WTP	a threshold value of zero, At a WTP threshold of zero, there was a 95% probability that MI was cost-effective. At the WTP threshold, 66.3% bootstrapped iterations were dominant.	
Maes et al. 2014 [73*]	CUA	Total costs per person	Intervention: $7392; Control: $7035	HUI	Intervention: 0.63 (baseline) to 0.65 (follow up); Control: 0.64 (baseline) to 0.61 (follow up)	ICER (cost per QALY gained):	$10,456 per QALY (health-care perspective); $24,580 per QALY (societal perspective)	Bootstrapping Y/N (iterations):	Y; (1000 replications)	Cost effective
		Healthcare costs	Intervention: $4034; Control: $3882							
		Patient/family costs	Intervention: $106; Control: $135					WTP Threshold(s)	$45,000 for a QALY gain	
		Productivity losses	Intervention: $3252; Control: $3018					Probability cost-effective at WTP	68% from the healthcare perspective; from societal perspective 58% (52% in complete case analysis).	
Parry et al. 2012 [85*]	CEA and CUA	Costs:	Resource/service use indicators used as a proxy for costs. Text reports “no statistical differences between the groups on any of the service use indicators, although there was a slight increase in GP consultations in the CBT group during the treatment period”	Change in ASC panic-fear score	Intervention: −5.04 (SD 6.20); Control: −2.43 (SD 5.54)	ICER (cost per QALY gained):	Economic evaluation not conducted. No ICERs for change in ASC score. Data suggest intervention would be dominated due to EQ-5D scores being reduced in treatment arm.			No reported conclusion on cost effectiveness (unlikely to be cost-effective).
		Average cost of intervention (per participant)	£378-£798	Difference in EQ-5D score between intervention and control group at 6 months (ANCOVA)	ITT analysis: −0.11 (95% CI: − 0.20 to − 0.03; p0.012); Complete case analysis: 0.12 (95% CI: 0.25 to 0.02); Intervention group had significantly lower scores.					
Rolving et al. 2016 [89*]	CEA and CUA	Average total costs at 12 months follow up:	Intervention: €52,492; Control: €52,580	Change in ODI score at 3 months	Intervention: −14.8 (−18.7; −10.9); Control: −4.0 (−10.3; − 2.3);	ICER (cost per QALY gained):	Not reported as costs intervention dominant (costs less and more effective than control).	Sensitivity Analysis Used	different imputation strategies	Cost effective per QALY gained (for ODI it depends on society’s WTP for 15 point gain)
		Extra costs related to the intervention:	Intervention: €610 (production loss), €630 (intervention costs) and €116 (travel expenses); Control: N/A	Change in ODI score at 6 months	Intervention: −15.2 (− 18.8; − 11.6); Control: −8.4 (− 14.6; − 2.2)			Bootstrapping Y/N (iterations):	Y; 10,000 replications	
				Change in ODI score at 9 months	Intervention: − 14.9 (− 18.4; −11.5) Control: − 10.0 (−16.6; −3.3);			WTP Threshold(s)	€40,000 for one additional QALY, €10,000 per 15 point gain in Oswerty Disability Index (ODI)	
				Change in EQ-5D utility score at 12 months from baseline [NB: may have been measured at other time points as reportedly only significant at the 3 month time point]	Intervention: 0.135; Control: 0.129			Probability cost-effective at WTP	70% per QALY gained, 90% per 15 point gain in ODI	
				Change in QALYs at 12 months from baseline	Intervention: 0.71; Control: 0.636					
Tyrer et al. 2014 [94*]	CEA and CUA	Mean total health and social care costs over 24 months	Intervention: £7314.20; Control: £7727.40	Mean (SD) improvement from baseline on the HAI	Intervention: 5·90 (7·54); Control: 3·66 (6·57)	ICER (cost per 1 point improvement in HAI scale)	£55.86	Bootstrapping Y/N (iterations):	Y; (number of iterations not reported)	Unclear
		Mean costs (range) of the intervention (mean of 6 sessions)	Intervention: £421.51 (£0-£2383); Control: N/A	Mean gain in utility score (EQ-5D) from baseline at 24 months	Intervention: 0.085; Control: 0.065					
				Mean QALY gain from baseline to 24 months	Intervention: 1.108 QALYs; Control: 1.097 QALYs 95% CI: 95% CI is −0.091 to 0.087; *p* = 0.964.	ICER (cost per QALY gained):	£14,169 per QALY gained (however as QALY 95% Cis include zero there was no evidence on cost-effectiveness plane that CBT-HA is cost-effective in terms of health-related quality of life)	WTP Threshold(s)	£20,000-£30,000 per QALY gained	
								Probability cost-effective at WTP	The probability that the intervention is cost effective exceeds 50% if society’s willingness to pay for a 1 unit change in HAI is at least £53 or more. There is a slightly higher probability of standard care being more cost-effective than CBT-HA. This finding is due to variability in the data, resulting in wide confidence intervals, and very small differences in QALYs.	
van der Aa et al. 2017 [96*]	CEA and CUA	Mean (SE) patient costs over 24 months	Intervention: €21,931 euros (€2035); Control: €22,808 euros (€2956). Mean difference not significant (−€1154; 95% CI − 7708 to 4328).	Mean change (SE) in HADS-A score; Difference between groups (95% CI)	Intervention: 1.88 (0.47); Control 0.45 (0.51); Mean difference 1.43 (95% CI 0.10 to 2.77)		Intervention dominant (ICER: − 613). Conclusions do not change with healthcare only perspective.	Sensitivity Analysis Used	Varying perspectives (healthcare only and human capital approach to include productivity losses);	Cost-effectiveness depends on willingness to pay threshold of decision makers
				Mean change in CES-D score	Intervention: 6.40 (1.05); Control: 3.67 (0.99); Mean difference 2.73, 95% CI −0.28 to 5.74, not statistically significant.		Intervention dominant (ICER: − 321). Conclusions do not change with healthcare only perspective;	Bootstrapping Y/N (iterations):	Y; (5000 iterations)	
				Incidence of depression/anxiety at 24 month follow up	Intervention: 0.29; Control: 0.46; Mean difference: 0.17 which was statistically significant (95% CI 0.06 to 0.29).		Intervention dominant (ICER negative: − 5159) indicating that to prevent one case of depression or anxiety €5159 is saved in the stepped-care group as compared to usual care. Conclusions do not change with healthcare only perspective;	WTP Threshold(s)	Change in score (HADS-A and CES-D):€0 - €4000; Per disorder prevented: €0 - €33,000; €0 - €20,000 per QALY gained	
				QALYs gained (SE); Difference between groups (95% CI)	Intervention 1.32 (0.04); Control 1.28 (0.04); Mean difference 0.03 (95% CI −0.09 to 0.15), not statistically significant		Intervention dominant (ICER of −29,233 euros per QALY). Conclusions do not change with healthcare only perspective	Probability cost-effective at WTP	For the CES-D and the HADS-A, 60% at €0 per point improvement on the CES-D/HADSA; this increased to 95% or more at a WTP of €2500 per point improvement on the CES-D and €4000 per point improvement on the HADS-A; Per disorder prevented, 59% aat €0. At a WTP of €10,000 this was 77%, and at€20,000 it was 88%, and increased to 95% or more at a WTP of €33,000 per disorder prevented; Per QALY gained, 59% at a threshold of €0, this increased to 65% or more at a willingness-to-pay of €20,000 per QALY.	

Service use costs were not reduced in the CBT group during treatment, or in the 6 months after the treatment phase and the intervention itself cost an average of £378-£798 per participant depending on the number of sessions attended. The study only considered healthcare costs and no QALYs or ICERs were calculated.

## Dermatology (psoriasis)

No systematic reviews of the cost effectiveness of psychological interventions in dermatology populations were identified.

### Dermatology study in current review

One Norwegian study conducted a cost-utility analysis of supported self-management with motivational interviewing for patients with psoriasis (see Tables 21 and 22). Larsen et al. [65*] examined telephone-based individualised motivational interviewing, as a follow-up to a 3 week climate therapy/heliotherapy (CHT) programme, compared to TAU following the CHT programme. At 6 months post-CHT, the intervention group had a lower cost than the TAU group, with a mean difference of 1780 euros and the authors concluded that as motivational interviewing provided equivalent quality of life and utility, at reduced costs, it could be considered cost-effective.

## Medical patients in secondary care settings

No systematic reviews of the cost effectiveness of psychological interventions in general secondary care settings were identified.

### Medical patients in secondary care settings study in current review

Tyrer et al. [94*] considered the cost-effectiveness of CBT for health anxiety in UK medical patients in secondary care – including cardiac, endocrine, gastroenterological, neurological, and respiratory medicine clinics (see Tables 21 and 22). The intervention involved five to ten sessions of CBT for health anxiety (CBT-HA), delivered by staff trained specifically for the intervention and supervised by researchers to ensure consistency in treatment. There was no evidence of an effect on social functioning or quality of life, and therefore no evidence of cost-effectiveness in terms of QALYs. However, the study found that CBT-HA resulted in significant improvements in health anxiety with no significant difference in health and social care costs compared to standard care. The authors suggested the findings indicate that staff trained to deliver CBT-HA in medical clinics would help to relieve substantially troubling anxiety in a more cost-effective manner than current standard approaches.

## Surgery (lumbar spinal fusion surgery)

No systematic reviews of the cost effectiveness of psychological interventions in surgical populations were identified.

### Surgery study in current review

Rolving et al. [89*] conducted a cost-effectiveness analysis of group-based CBT compared to usual care for patients undergoing lumbar spinal fusion surgery in Denmark (see Tables 21 and 22). The intervention consisted of six, three-hour sessions (four prior to surgery and two post-surgery at 3 and 6 months), delivered by a multidisciplinary team, which had received a 2 day training programme on the manual-based intervention. After 12 months, the estimated QALY (based on EQ-5D scores) was significantly higher for the CBT group and there was no difference in the overall societal costs. The authors calculated a 70% chance of CBT being cost-effective compared to usual care at a willingness-to-pay of 40,000 euros per QALY. They conclude that the findings support the implementation of such an intervention for patients undergoing lumbar spinal fusion surgery in Denmark.

## Tinnitus

No systematic reviews of the cost effectiveness of psychological interventions in tinnitus populations were identified.

### Tinnitus study in current review

A single primary RCT conducted by Maes et al. [73*] considered the cost-effectiveness of CBT-based treatment versus usual care for tinnitus (see Tables 21 and 22). The stepped care intervention included an individual consultation with a psychologist and then a stepped programme comprising key elements of CBT for those with moderate tinnitus (12 weekly group sessions) or severe tinnitus (24 biweekly group sessions). The authors found an ICER of $10,456 per QALY from a health-care perspective, and $24,580 per QALY from a societal perspective. The probability that the intervention is cost-effective from a societal perspective was 58% for a willingness to pay of $45,000 per QALY.

## Vision impairment in older adults

No systematic reviews of the cost effectiveness of psychological interventions in visually impaired populations were identified.

### Vision impairment in older adults study in current review

A Dutch study by Van der Aa et al. [96*] evaluated a stepped care intervention for depression and anxiety in older adults with vision impairment, including macular degeneration, glaucoma, cataract, diabetic retinopathy, and cerebral haemorrhage (see Tables 21 and 22). Depending on persistence of symptoms, care could involve a guided self-help course based on CBT followed problem solving treatment with trained social workers and psychologists if symptoms continued. In the study, 56% of the intervention group received the CBT-based course and 22% went on to receive problem solving treatment. The economic evaluation found that the stepped-care intervention was dominant to usual care, with a probability of around 60%, in treating mental health problems in visually impaired older adults. The probability of cost-effectiveness was 95% or more at a willingness-to-pay of 33,000 euros per depressive and/or anxiety disorder prevented. In terms of QALYs, the probability that stepped-care was cost-effective compared to usual care was 65% or more at a willingness to pay of 20,000 euros per QALY.

## Discussion

### Summary of findings

A diverse range of studies published since 2012 provides considerable support for the cost effectiveness of psychological interventions for patients experiencing different physical health conditions in varied contexts. The most prevalent type of psychological interventions included in this review are those based on a cognitive behaviour therapy approach. Studies cover a wide range of settings, populations, time- horizons, medical conditions and methods. A major challenge therefore is establishing the appropriate degree of confidence when extrapolating from these results.

Overall, evidence published in the field of chronic pain appears to be some of the most clear-cut in demonstrating cost effectiveness, including interventions for non-specific chronic pain [45, 62], chronic low back pain [46, 83] and fibromyalgia [17, 65, 71, 72]. Interventions include cognitive behavioural based approaches delivered through internet or groups, ACT, exposure and psycho-education, with clinical outcomes often as good as control conditions such as recommended drug therapy, but with cost savings.

In cost effectiveness studies of psychological interventions for patients with cancer, cognitive behavioural approaches in various modalities of delivery again stand out as having the strongest evidence for being cost effective, although there is evidence for the cost effectiveness of other types of psychological interventions including mindfulness based groups and meaning centred group psychotherapy. Where psychological interventions target individuals experiencing more severe distress, offering a stepped model of intervention, there is stronger indication of cost effectiveness [41, 58, 97].

In the field of diabetes, there have been only three cost-effectiveness or cost utility studies over the past 9 years [39, 57, 81]. Two studies concluded that psychological interventions delivered as part of collaborative care had a high probability of being cost effective [39, 81], whereas a third, nurse delivered cognitive behavioural and motivational interviewing based brief intervention, did not change glycaemic control and was unlikely to be cost effective [57*]. A mixed picture also emerges in cardiac studies. A cognitive behavioural based self-management programme provided little evidence of cost effectiveness on the cost of care when compared to usual care [75*], whereas, other authors have reported more convincing evidence for the cost effectiveness of problem solving therapy and / or anti-depressants in cardiac settings [63*]. In a study of non-cardiac chest pain, patients who received a cognitive behavioural based intervention had fewer hospital admissions or A & E visits, however the cost differential compared with usual medical care was not found to be significant [94*].

Two studies of behavioural therapy interventions for patients who have experienced stroke report mixed evidence and conclusions [54, 99]. A study of behavioural therapy addressing post-stroke aphasia concluded that in terms of cost effectiveness results were promising but recommended further investigation [54*], while a cognitive behavioural based intervention for post stroke depressive symptoms, although promising, was not convincingly cost effective in terms of quality of life [99*].

Of the five studies included in this review focussing on MS, all were found to be clinically effective but cost effectiveness results were variable. A group based intervention focusing on psychological adjustment was found to be cost effective when compared with usual care for people with MS and low mood [52*], however, a cognitive behavioural based group focussing on MS fatigue [55*] did not result in clear cost effectiveness, although there was clinically significant improvement in fatigue. The results of another cognitive behaviour based intervention, which was delivered by Skype [36*], was highly likely to be cost effective. An internet based self-management programme supported by telephone follow-up also showed promise in terms of cost-effectiveness but had a small sample size [80]. In contrast a nurse-led cognitive behaviour intervention [79*] was effective in reducing distress but unlikely to be cost effective in comparison to supportive listening.

Three studies of cognitive behaviour based approaches for insomnia reported in this review also present a mixed picture of cost effectiveness. Two suggest that results were promising but highlight study limitations and call for further research [37, 107], while the third, which included non-health related indirect costs was much more conclusive in supporting the cost effectiveness of this intervention for insomnia [93*].

Four studies were identified in the field of medically unexplained physical symptoms category in this review (a range of terms were used by the different study authors to describe this population of patients). Two of these reached positive conclusions about the cost effectiveness of group based cognitive behaviour based approach [91, 103]. Two further studies explored the cost effectiveness of Psychodynamic interpersonal therapy [43*] and Mindfulness based cognitive therapy [101*], both concluding that the cost effectiveness of these interventions was uncertain.

Other studies have considered the cost effectiveness of individual or group cognitive behavioural approaches for a wide range of patient groups including medical patients in secondary care with health anxiety [94*], patients undergoing lumbar spinal fusion [89*], patients with asthma [85*], those with tinnitus [73*] and older adults with visual impairment [96*]. In all of the above studies, the authors concluded that cognitive behavioural based interventions had a high probability of being cost effective, supporting the implementation of these interventions in the settings in which the studies took place. In two of these five studies, a cognitive behaviour based approach was offered in the context of a stepped care intervention. A cognitive behaviour based weight loss intervention [51*] was found to be both clinically effective and cost effective when compared with projected medical costs when trialled across a range of treatment modalities (non-interactive or interactive web-based, with and without coaching support). For dermatology patients with psoriasis, motivational interviewing was also considered to be cost effective [65*].

### Methodological issues and comparability

The studies reported in this review span a wide range of settings and methodological approaches which makes it impossible to produce a valid quantitative synthesis. The results are therefore presented in a narrative format.

26% of the studies were undertaken in the UK, with 46% from other European countries, with the remainder from US, Australia and Japan. While it might be assumed that populations will be similar in these developed countries and interventions equally effective, the structure of health care systems are very different [117]. Healthcare costs will vary greatly and there may also be significant variation in what constitutes usual care. Readers, and service commissioners in particular, are invited to consider how comparable the health care systems are in these studies to the ones that operate in their own context.

Meaningful comparisons are further limited by the fact that different methodological approaches have been employed across studies including cost effectiveness and cost utility analyses. There is therefore wide variation in the breadth of health or societal costs and benefits considered in different studies.

In cost effectiveness analyses which explore the cost of producing a clinically meaningful change, agreed “willingness to pay” thresholds vary in different settings and funding systems for health care. QALYs are used to measure generic aspects of health (regardless of condition) in a single unit, so in theory this allows comparison of different health conditions and programmes in the same terms. Where economic evaluation is undertaken alongside randomised controlled trials, QALYs are seen as the preferred outcome measure for many health system funders [118], however, a number of studies, deemed by the authors to be of a good or acceptable standard, did not capture outcomes in terms of QALYs, limiting comparability of these studies.

Economic evaluation typically employs incremental cost effectiveness ratios or ICERs to compare the costs and effects of two different approaches expressing them as a ratio. Most studies of this kind were trial based evaluations that used bootstrapping to estimate uncertainty in the ratio to generate confidence intervals, however, some used different types of sensitivity analysis in addition or as standalone alternatives e.g., scenario analyses. The extent to which some types of sensitivity analysis are possible can be dependent on uncertainty in the clinical evidence base, which can be considerable when RCTs are being undertaken (as without equipoise there would be no need to conduct a trial), so the widespread use of bootstrapping is not surprising under the circumstances, given that it makes no assumptions about underlying population distributions and instead uses the sample data to explore uncertainty around the results.

Further methodological differences between reported studies which hinder comparisons include the perspective adopted, the time horizon and the resources included. Most studies focus on some costs in addition to direct healthcare. The vast majority of studies focused on health and social care costs although often in addition to wider societal perspectives as a secondary analysis e.g., costs of lost productivity through absenteeism. Others included some consideration of the patient and family or carer costs. While wider perspectives are valid, healthcare costs are obviously of most relevance to policymakers in NHS settings. Regardless of perspective, it is another factor that limits comparison between studies.

Most studies employed a time horizon of up to 12 months, that is, the duration over which health outcomes and costs were calculated in the clinical trial. Studies ranged from 8 weeks to 10 years (based on modelling for longest time horizons). Longer time horizons may affect the magnitude of the findings, particularly for long term conditions and it may be unfeasible to populate longer term models. Conversely, a longer time horizon of 12 months or less may be too short to the capture the full extent of long-term costs or cost savings and again where this varies across studies, direct comparison is not possible. There was significant variation in the time horizons employed and the extent and nature of economic modelling undertaken. The majority of studies (28) report incremental cost effectiveness ratios (ICERs). A similar proportion of studies used bootstrapping, a statistical technique for estimating confidence intervals for cost effectiveness ratios, as a sensitivity analysis.

In terms of the target of the interventions evaluated in this review, these included psychological distress, severity of mental health symptoms, severity of physical symptoms and quality of life in general. Many studies used quality of life rating scales as the main indicator of clinical effectiveness, such as the EQ5D or the SF36. For most physical health conditions and symptoms, these measures have been found to have good sensitivity and validity, however, their sensitivity for patients with significant mental health difficulties, including anxiety, has been questioned [119]. Where interventions target mental health symptoms, generic quality of life measures may not be the most reliable measure of effectiveness. A wide range of other effectiveness measures are used in different studies, reflecting different psychological treatment targets, including conditions specific measures, measures of symptoms such as pain or fatigue severity, weight loss and measures of psychological distress, such as the Hospital Anxiety and Depression Scale [120].

### Strengths and limitations of this review

The search strategy identified a very wide range of studies which were reviewed by the team as a whole before final decisions were reached about whether or not to include each of these in the final 46 which were deemed to meet the inclusion criteria. As this review built on a previous study, the date range covered was relatively narrow from 2012 to 2018. Only studies published in English were included and only a small number of these were UK based studies. As outlined above, caution is needed in extrapolating from the results.

The search strategy was devised and completed by one member of the team and was not peer reviewed or discussed by the review team as a whole. The search strategy was however similar to others in the published literature. The team also checked the papers included in other systematic reviews and literature reviews to ensure that no studies that met the inclusion criteria had been missed.

A strength of this review is its comprehensiveness, particularly in terms of assessing the quality of the papers that were included. The review team systematically evaluated the quality of both the original RCT and the economic evaluation using the SIGN methodology checklists for RCTs and economic evaluations.. In most cases this required the review team to go back to the original RCT publications as well as the health economic study, in order to be able to assess its quality. All studies included in the review were deemed to be either acceptable or high quality. Inter-rated reliability was checked through double rating 50 % of papers, however, ideally all papers would be double rated.

Quantitative meta analysis was not possible because of the wide variation in methods and range of interventions, conditions and settings in the studies that were included. A thorough qualitative meta synthesis has been undertaken, however, as well as a narrative account of the review and RCT based studies. The breadth of studies included allowed the authors to include all relevant information and evidence of cost effectiveness which may be of value to clinicians and policy makers in reaching decisions about psychological interventions in physical health settings.

The validity of our conclusions is dependent on the validity of the descriptions / definitions used by the authors of the papers included in the study. Caution is needed however in reaching conclusions about therapeutic interventions such as “cognitive behaviour therapy”. In many instances, studies have evaluated a “cognitive behaviourally based” group or individual intervention.

The literature search and synthesis of these papers was started before the COVID-19 pandemic and the subsequent implications of this delayed further work on this process. This is a rapidly expanding field. Inevitably other work will have been published during the delay in finalising the review.

### Comparison with other studies and future developments

The findings of this review are broadly in line with integrating psychological interventions into the overall treatment for a range of long term conditions and for medically unexplained symptoms can have significant economic benefits for both threshold and sub threshold psychological problems and in the management of a range of debilitating physical symptoms. The cost effectiveness evidence is strongest in relation to interventions for patients with chronic pain and cancer. As outlined above, caution is required in interpreting conclusions and extrapolating to a UK context due to both methodological limitations and lack of comparability of studies. This review highlights the need for further economic evaluations based in the UK, particularly in fields where none have been undertaken in recent years, such as cancer.

Throughout 2020 as a result of the COVID-19 pandemic, delivery of psychological interventions in clinical practice shifted significantly to remote delivery by phone or video rather than face to face, and interactive web based packages of intervention have developed rapidly. A number of studies reported in this review indicate that technology enabled delivery of psychological interventions can be clinically effective and cost-effective in a range of physical conditions, however, evidence is limited, particularly for the delivery of group interventions. With increased availability of technology and acceptability to both clients and health professionals, technology enabled psychological therapy and interventions looks set to remain a very significant part of the delivery of psycho-social interventions across health care settings and further research into clinical and cost effectiveness is much needed. Studies need to be more explicit about the type of delivery of interventions being studied, without combining different modes of delivery within one condition. A wide range of relevant issues may be relevant for cost effectiveness studies, for example, whether remote delivery reduces costs such as travel time, time off work or clinical accommodation costs. Careful consideration should also be given to participant attrition; health inequalities may make internet-based interventions harder to access for some of those at risk of higher health costs.

Having developed the methodology for this review, there is scope to update the search and synthesis as the literature and clinical practice evolve.

## Conclusion

Three quarters of the studies included in this review conclude that interventions applying psychological approaches in physical health settings are clearly cost effective or likely to be cost effective compared to usual care. Of the wide range of approaches included in this review, the strongest evidence for cost effectiveness overall is for studies offering cognitive behavioural approaches and those which employ a stepped care approach which targets those with most severe difficulties. The health economic case is very strong for cost effectiveness of interventions in chronic pain. A strong case is also presented for many interventions in the field of cancer and a number of other specific health conditions, although none of cancer studies were undertaken in the UK. The picture is more mixed for studies based in cardiac, diabetes and stroke services all of which are common and costly long term conditions.

Given the prevalence of mental health problems in those living with long term conditions, and the impact of mental health and health behaviours on the course and costs of long term conditions, there have been relatively few robust studies published. Caution is needed as the number of published studies for each area is relatively small and of these, only a small percentage have been undertaken in the UK so may not generalise to a British or more specifically, Scottish context. Confidence in these findings is likely to increase when more studies are undertaken. Clearly we would argue that it would be most helpful for these to be conducted in a UK healthcare context. This review has not therefore provided certainty about the cost effectiveness of psychological approaches in all areas of physical health, however, it presents a strong case for continuing to develop psychological services for patients presenting in physical health settings, and a clear need for more economic evaluations of widely delivered psychological interventions to be undertaken in context of the NHS and partnership settings.

## Supplementary Information

**Additional file 1.**

## Data Availability

The data and materials on which this review is based are available in the included articles with details of the synthesis reproduced in the paper’s results tables. All data generated or analysed during this study are included in this published article [and its supplementary information files].

## Abbreviations

15D: Health-related quality of life (HRQoL) instrument, developed in Finland
ACT: Acceptance and Commitment Therapy. Developed by Steven Hayes in 1982
BDI: Beck Depression Inventory
Bootstrapping: A statistical technique for estimating confidence intervals for cost effectiveness ratios
CBT: Cognitive Behavioural Therapy. This type of therapy helps people to improve their psychological wellbeing by focusing on more helpful thoughts and behavioural patterns
CEA: Cost Effectiveness Analysis, “Focuses on the best way of meeting a stated objective given that some means of pursuing it is going ahead. The objective of the programme is not being, and cannot be, questioned.” (BMJ Best Practice glossary of health economics terms)
CUA: Cost Utility Analysis, “A form of cost-effectiveness analysis where benefits are measured in terms of a utility measure such as the quality-adjusted life year (QALY)” (BMJ Best Practice glossary of health economics terms)
CPAQ: Chronic Pain Acceptance Questionnaire
DFD: Depression Free Days
Dominant: A ‘dominant’ intervention is one that is both less costly and more effective than a comparator intervention
Economic evaluation: “The comparison of alternative courses of action in terms of their costs and consequences, with a view to making a choice” (BMJ Best Practice glossary of health economics terms)
EORTC-QLC-C30: Questionnaire developed to assess the quality of life of cancer patients (European Organisation for Research and Treatment of Cancer)
EQ-5D: Questionnaire to measure quality of life on 5 dimensions, developed by EuroQol
EQ-5D-3L: Three level version of EQ-5D
EQ-5D-5L: Five level version of EQ-5D
EUC: Enhanced usual care
FSS: Fatigue Severity Scale
FIQ: Fibromyalgia Impact Questionnaire
GHQ: General Health Questionnaire, a screening tool for mental health that can be used in primary care or outpatient settings. By Goldberg.
HADS: Hospital Anxiety and Depression Scale by Zigmond and Snaith (1983)
HUI: Health Utilities Index, a rating scale used to measure general health status and health-related quality of life
ICER: Incremental Cost Effectiveness Ratio, “Obtained by dividing the difference between the costs of the two interventions by the difference in the outcomes (i.e., the extra cost per extra unit of effect).” (BMJ Best Practice glossary of health economics terms)
ISI: Insomnia Severity Index
LMHAQ-CP: Lucock and Morley Health Anxiety Questionnaire – Chest Pain
MBCT: Mindfulness Based Cognitive Therapy. Combines mindfulness and cognitive behavioural therapy principles, and was originally devised to prevent depressive relapse
MBSR: Mindfulness Based stress reduction. Developed by Jon Kabat-Zinn, this treatment involves a course of 8 mindfulness sessions to treat stress, anxiety and difficulties associated with physical health
MCID: Minimal clinically important difference
MSIS-29: Multiple Sclerosis Impact Scale
NRS: Numeric Rating Scale, to assess pain intensity for persons able to self-report
Psychoeducation: A term to describe information and educational interventions that incorporate psychological teaching elements
Psychological Intervention: Non pharmacological interventions (for example teaching relaxation skills) which are based on psychological principles to improve quality of life and alleviate mental distress
Psychological Therapy: Non pharmacological talking therapies (for example Cognitive Behavioural Therapy) are based on psychological principles to treat mental health conditions, and further specialist training is required prior to delivery. Therapeutic delivery may be manualised and supervised
QALYs: Quality-Adjusted Life Years, “Calculated by adjusting the estimated number of life-years an individual is expected to gain from an intervention for the expected quality of life in those years. The quality of life score will range between 0 for death, to 1 for perfect health…”. (BMJ Best Practice glossary of health economics terms)
RCI: Reliable Change Index, used to evaluate whether a change in a score between two points in time (e.g. pre-treatment to post-treatment) is considered statistically significant
RCT: Randomised Controlled Trial
SADQH21: Stroke Aphasic Depression Questionnaire Hospital (21 items)
SIGN: Scottish Intercollegiate Guidelines Network. https://www.sign.ac.uk/
Sensitivity analysis: “A process through which the robustness of an economic model is assessed by examining the changes in results of the analysis when key variables are varied over a specified range.” (BMJ Best Practice glossary of health economics terms)
SEPS score: Schedule for Evaluating Persistent Symptoms, a method of recording medically unexplained symptoms
SF-12: Short-Form Health Survey 12, a shorter version of the SF-36 measure of health-related quality of life
SF-36: Short-Form Health Survey SF-36, measure of health-related quality of life
SF-6D: A classification for describing health derived from a selection of SF-36 items; allows the analyst to obtain quality adjusted life years (QALYs) from the SF-36 for use in cost utility analysis.
TAU: Treatment as usual
Utility score: A measure of quality of life used to calculate quality adjusted life years (QALYs)
VAS: Visual Analogue Scale, a pain measure in which pain is shown spatially as distance along a straight line
Willingness to pay: “This technique asks people to state explicitly the maximum amount they would be willing to pay to receive a particular benefit…” (BMJ Best Practice glossary of health economics terms)
