# Supplementary material for: A systematic review of economic analyses of psychological interventions and therapies in health-related settings
Source: BMC Health Serv Res. 2022 Sep 7;22:1131. doi: 10.1186/s12913-022-08158-0 (PMC9450839; doi:10.1186/s12913-022-08158-0)
Supplement: Supplementary file 1 — Additional file 1. [file 12913_2022_8158_MOESM1_ESM.docx]

Supplementary Information

Search Strategy

Details of the search strategy is outlined in the text box below.

| Medline:  1 psychology, clinical/ or exp psychology, medical/ or psychological techniques/ or interview, psychological/ or exp psychotherapy/  2 (psycholog* adj2 (intervention* or therap* or treatment* or service* or approach* or screen*)).mp.  3 motivational interview*.mp.  4 Cognitive Behavioral Therapy/ or (cognitive adj1 therap*).mp.  5 (psychotherap* or psycho therap*).mp.  6 1 or 2 or 3 or 4 or 5  7 (physical health or physical conditions or medical condition*).mp.  8 exp "diseases (non mesh)"/  9 (Asthma or Cancer or Cardiovascular Disease or Chronic Fatigue Syndrome or Chronic Kidney Disease or Chronic Obstructive Pulmonary Disease or Chronic Pain or Diabetes or Irritable Bowel Syndrome or Multiple Sclerosis or Osteoarthritis or Rheumatoid Arthritis or Overweight or Obesity or Neuropsychology or stroke or persistent physical symptoms or cystic fibrosis or chronic fatigue).mp.  10 7 or 8 or 9  11 6 and 10  12 Cost-Benefit Analysis/  13 Economics, Medical/  14 (cost* or sav* or efficien* or finan* or economic*).ti.  15 12 or 13 or 14  16 11 and 15  17 limit 16 to (english language and yr="2012 -Current")  18 study protocol.ti.  19 17 not 18  EMBASE (Ovid)  1 (physical health or physical conditions or medical condition*).mp.  2 (Asthma or Cancer or Cardiovascular Disease or Chronic Fatigue Syndrome or Chronic Kidney Disease or Chronic Obstructive Pulmonary Disease or Chronic Pain or Diabetes or Irritable Bowel Syndrome or Multiple Sclerosis or Osteoarthritis or Rheumatoid Arthritis or Overweight or Obesity or Neuropsychology or stroke or persistent physical symptoms or cystic fibrosis or chronic fatigue).mp.  3 exp physical disease/  4 1 or 2 or 3  5 exp "cost benefit analysis"/  6 exp "cost effectiveness analysis"/  7 (cost* or sav* or efficien* or finan* or economic*).ti.  8 5 or 6 or 7  9 clinical psychology/ or medical psychology/ or exp psychotherapy/  10 (psycholog* adj2 (intervention* or therap* or treatment* or service* or approach* or screen*)).mp.  11 motivational interview*.mp.  12 (((cognitive behavio* adj1 therap*) or cognitive) adj1 therap*).mp.  13 (psychotherap* or psycho therap*).mp.  14 9 or 10 or 11 or 12 or 13  15 4 and 8 and 14  16 study protocol.ti.  17 15 not 16  18 limit 17 to (english language and yr="2012 -Current")  S23 s19 not s20 Limiters - Publication Year: 2012-2018  Narrow by Language: - english  S22 s19 not s20 Limiters - Publication Year: 2012-2018  S21 s19 not s20  S20 TI study protocol  S19 S9 AND S12 AND S18  S18 S13 OR S14 OR S15 OR S16 OR S17  S17 psychotherap* or psycho therap*  S16 cognitive behavio* N1 therap*  S15 motivational interview*  S14 (psycholog* N2 (intervention* or therap* or treatment* or service* or approach* or screen*))  S13 DE "Psychotherapy" OR DE "Adlerian Psychotherapy" OR DE "Adolescent Psychotherapy" OR DE "Affirmative Therapy" OR DE "Analytical Psychotherapy" OR DE "Autogenic Training" OR DE "Behavior Therapy" OR DE "Brief Psychotherapy" OR DE "Brief Relational Therapy" OR DE "Child Psychotherapy" OR DE "Client Centered Therapy" OR DE "Cognitive Behavior Therapy" OR DE "Conversion Therapy" OR DE "Eclectic Psychotherapy" OR DE "Emotion Focused Therapy" OR DE "Existential Therapy" OR DE "Experiential Psychotherapy" OR DE "Expressive Psychotherapy" OR DE "Eye Movement Desensitization Therapy" OR DE "Feminist Therapy" OR DE "Geriatric Psychotherapy" OR DE "Gestalt Therapy" OR DE "Group Psychotherapy" OR DE "Guided Imagery" OR DE "Humanistic Psychotherapy" OR DE "Hypnotherapy" OR DE "Individual Psychotherapy" OR DE "Insight Therapy" OR DE "Integrative Psychotherapy" OR DE "Interpersonal Psychotherapy" OR DE "Logotherapy" OR DE "Narrative Therapy" OR DE "Network Therapy" OR DE "Persuasion Therapy" OR DE "Primal Therapy" OR DE "Psychoanalysis" OR DE "Psychodrama" OR DE "Psychodynamic Psychotherapy" OR DE "Psychotherapeutic Counseling" OR DE "Rational Emotive Behavior Therapy" OR DE "Reality Therapy" OR DE "Relationship Therapy" OR DE "Solution Focused Therapy" OR DE "Supportive Psychotherapy" OR DE "Transactional Analysis" OR DE "Adolescent Psychotherapy" OR DE "Multisystemic Therapy" OR DE "Behavior Therapy" OR DE "Aversion Therapy" OR DE "Conversion Therapy" OR DE "Dialectical Behavior Therapy" OR DE "Exposure Therapy" OR DE "Implosive Therapy" OR DE "Reciprocal Inhibition Therapy" OR DE "Response Cost" OR DE "Systematic Desensitization Therapy" OR DE "Child Psychotherapy" OR DE "Play Therapy" OR DE "Cognitive Behavior Therapy" OR DE "Acceptance and Commitment Therapy" OR DE "Gestalt Therapy" OR DE "Empty Chair Technique" OR DE "Group Psychotherapy" OR DE "Encounter Group Therapy" OR DE "Therapeutic Community" OR DE "Humanistic Psychotherapy" OR DE "Client Centered Therapy" OR DE "Hypnotherapy" OR DE "Age Regression (Hypnotic)" OR DE "Ericksonian Psychotherapy" OR DE "Integrative Psychotherapy" OR DE "Schema Therapy" OR DE "Psychoanalysis" OR DE "Adlerian Psychotherapy" OR DE "Brief Relational Therapy" OR DE "Dream Analysis" OR DE "Self-Analysis" OR DE "Psychotherapeutic Counseling" OR DE "Family Therapy"  S12 S10 OR S11  S11 TI (cost* or sav* or efficien* or finan* or economic*)  S10 DE "Costs and Cost Analysis" OR DE "Health Care Costs" OR DE "Cost Containment" OR DE "Health Care Economics"  S9 S1 OR S2 OR S3 OR S4 OR S5 OR S6 OR S7 OR S8  S8 DE "Pain" OR DE "Aphagia" OR DE "Back Pain" OR DE "Chronic Pain" OR DE "Headache" OR DE "Myofascial Pain" OR DE "Neuralgia" OR DE "Neuropathic Pain" OR DE "Somatoform Pain Disorder" OR DE "Headache" OR DE "Migraine Headache" OR DE "Muscle Contraction Headache" OR DE "Neuralgia" OR DE "Trigeminal Neuralgia"  S7 DE "Physical Disorders" OR DE "Benign Neoplasms" OR DE "Breast Neoplasms" OR DE "Endocrine Neoplasms" OR DE "Leukemias" OR DE "Melanoma" OR DE "Metastasis" OR DE "Nervous System Neoplasms" OR DE "Brain Neoplasms" OR DE "Glioma" OR DE "Terminal Cancer"  S6 DE "Apnea" OR DE "Sleep Apnea" OR DE "Bronchial Disorders" OR DE "Dyspnea" OR DE "Asthma" OR DE "Hay Fever" OR DE "Hyperventilation" OR DE "Laryngeal Disorders" OR DE "Lung Disorders" OR DE "Chronic Obstructive Pulmonary Disease" OR DE "Cystic Fibrosis" OR DE "Pneumonia" OR DE "Pulmonary Emphysema" OR DE "Pulmonary Tuberculosis" OR DE "Pharyngeal Disorders"  S5 DE "Aneurysms" OR DE "Arteriosclerosis" OR DE "Atherosclerosis" OR DE "Cerebral Arteriosclerosis" OR DE "Blood Pressure Disorders" OR DE "Hypertension" OR DE "Hypotension" OR DE "Syncope" OR DE "Cerebrovascular Disorders" OR DE "Cerebral Arteriosclerosis" OR DE "Cerebral Hemorrhage" OR DE "Cerebral Ischemia" OR DE "Cerebral Small Vessel Disease" OR DE "Cerebrovascular Accidents" OR DE "Subarachnoid Hemorrhage" OR DE "Heart Disorders" OR DE "Angina Pectoris" OR DE "Arrhythmias (Heart)" OR DE "Coronary Thromboses" OR DE "Myocardial Infarctions" OR DE "Hemorrhage" OR DE "Cerebral Hemorrhage" OR DE "Hematoma" OR DE "Subarachnoid Hemorrhage" OR DE "Hypertension" OR DE "Essential Hypertension" OR DE "Ischemia" OR DE "Cerebral Ischemia" OR DE "Thromboses" OR DE "Coronary Thromboses"  S4 DE "Bone Disorders" OR DE "Osteoporosis" OR DE "Joint Disorders" OR DE "Arthritis" OR DE "Muscular Disorders" OR DE "Cataplexy" OR DE "Fibromyalgia" OR DE "Muscular Atrophy" OR DE "Muscular Dystrophy" OR DE "Myasthenia Gravis" OR DE "Myofascial Pain" OR DE "Myotonia" OR DE "Torticollis"  S3 DE "Physical Disorders" OR DE "Blood and Lymphatic Disorders" OR DE "Cachexia" OR DE "Cardiovascular Disorders" OR DE "Chronically Ill Children" OR DE "Digestive System Disorders" OR DE "Endocrine Disorders" OR DE "Genetic Disorders" OR DE "Health Impairments" OR DE "Immunologic Disorders" OR DE "Infectious Disorders" OR DE "Metabolism Disorders" OR DE "Musculoskeletal Disorders" OR DE "Neonatal Disorders" OR DE "Neoplasms" OR DE "Nervous System Disorders" OR DE "Nutritional Deficiencies" OR DE "Respiratory Tract Disorders" OR DE "Sense Organ Disorders" OR DE "Sensory System Disorders" OR DE "Skin Disorders" OR DE "Toxic Disorders" OR DE "Urogenital Disorders" OR DE "Vision Disorders"  S2 (Asthma or Cancer or Cardiovascular Disease or Chronic Fatigue Syndrome or Chronic Kidney Disease or Chronic Obstructive Pulmonary Disease or Chronic Pain or Diabetes or Irritable Bowel Syndrome or Multiple Sclerosis or Osteoarthritis or Rheumatoid Arthritis or Overweight or Obesity or Neuropsychology or stroke or persistent physical symptoms or cystic fibrosis or chronic fatigue)  S1 physical health or physical conditions or medical condition* |
| --- |
